# Path Selective Photoinduced Energy and Electron Transfer in a Bis(acridinium‐Zn(II) Porphyrin)‐tetrapyridyl Porphyrin Host‐Guest Complex

**DOI:** 10.1002/chem.202502604

**Published:** 2025-09-19

**Authors:** Federica Ruani, Daniele Veclani, Daniel Sanchez‐Resa, Amy Edo‐Osagie, Geordie Creste, Andrea Barbieri, Valérie Heitz, Henri‐Pierre Jacquot de Rouville, Nicola Armaroli, Barbara Ventura

**Affiliations:** ^1^ Istituto per la Sintesi Organica e la Fotoreattività (ISOF) Consiglio Nazionale delle Ricerche (CNR) Via Piero Gobetti 101 Bologna 40129 Italy; ^2^ Laboratoire de Synthèse des Assemblages Moléculaires Multifonctionnels Institut de Chimie de Strasbourg, CNRS/UMR 7177 Rue Blaise Pascal, 4 Strasbourg 67000 France

**Keywords:** acridinium, charge transfer state, photoinduced electron transfer, tetrapyridyl porphyrin, Zn porphyrin

## Abstract

The 1:1 complex of a bis(acridinium‐Zn(II)porphyrin) host and a tetrapyridyl free‐base porphyrin guest shows a remarkable interplay of energy and electron transfer (eT) processes, finely tuned by the solvent polarity or the selection of the photoexcited component. A more polar environment (CH_2_Cl_2_) favors uncommon parallel intramolecular and intermolecular eT processes, while in an apolar solvent (toluene) the intermolecular eT process is hampered. Notably, the apolar environment also enables a rare energy transfer process from the porphyrin guest toward the charge‐transfer emissive state of the tweezer host. In total, no less than four different excited states were probed, which can be sketched by an inverted pyramid diagram highlighting directional path selection.

## Graphical abstract

Tuning unusual energy and eT processes in a bis(acridinium‐Zn(II) porphyrin)‐tetrapyridyl porphyrin host‐guest complex.

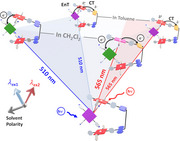

## 1 Introduction

Supramolecular architectures featuring photoinduced energy (EnT) and electron transfer (eT) processes are of fundamental importance for the development of artificial photosynthetic systems, because they mimic light harvesting and charge separation events occurring in natural photosynthetic organisms.^[^ ^1^ ^]^ In these artificial systems, according to the number of donor and acceptor units attached to the photosensitizer, complex photoinduced eT processes can take place.^[^ ^2^ ^]^ Interestingly, the direction of the eT can be switched by changing the redox states,^[^ ^2^, ^3^ ^]^ photo‐addressing a specific unit of the system,^[^ ^4^ ^]^ adding chemicals,^[^ ^5^ ^]^ stimulating photoconversion^[^ ^6^ ^]^ or changing the polarity of the solvent.^[^ ^7^ ^]^ The eT can also be triggered by an earlier EnT process (antenna effect).^[^ ^8^ ^]^ However, supramolecular systems combining several of these mechanisms are still rare,^[^ ^9^ ^]^ even though they open up new avenues for controlling directional eT processes. Such systems could prove very useful for optoelectronic and energy harvesting devices, logic gates, switches, and sensors.^[^ ^10^ ^]^

Porphyrins have been extensively used in supramolecular chemistry for the construction of highly sophisticated arrays.^[^ ^11^ ^]^ Metalated porphyrins, in particular, can axially bind selected ligands, enabling the self‐assembly of non‐covalent light‐responsive multicomponent systems.^[^ ^11^, ^12^ ^]^ Specifically, architectures embedding metalated porphyrins and pyridyl‐decorated components have been widely reported,^[^ ^11^, ^12^, ^13^ ^]^ with some of them exhibiting photoinduced EnT or eT processes between the porphyrin moieties.^[^ ^12^, ^14^ ^]^

We recently reported a bis(acridinium‐porphyrin) molecular tweezer^[^ ^15^ ^]^ (**1^2+^**) made of two Zn(II) porphyrin coordinating sites interconnected via two N‐acridinium units.^[^ ^16^ ^]^ This system responds to electrochemical and photochemical inputs, affording a sophisticated multicomponent switch.^[^ ^15^, ^16^ ^]^ It was shown that **1^2+^** undergoes ultrafast photoinduced eT from the porphyrin donor to the acridinium acceptor,^[^ ^15^ ^]^ leading to fluorescence quenching of both the porphyrin and the acridinium units. We also studied the ability of **1^2+^** to coordinate a non‐photoactive 4,4′‐bipyridine guest, revealing a twelve‐state supramolecular receptor behavior in response to light, redox, and chemical stimuli.^[^ ^15^ ^]^

Herein, we investigate the coordination capability of **1^2+^** for *meso*‐(5,10,15,20‐tetra(4′‐pyridyl))porphyrin (**TPyP**) as a photoactive guest. This porphyrin is particularly interesting since, thanks to its four pyridyl residues, it can act as a multitopic guest and has peculiar electrochemical properties with respect to other free‐base porphyrins.^[^ ^12^, ^14^ ^]^ This guest can (i) modify both its and the host's electronic properties through coordination, (ii) change the direction of eT, and (iii) open the way to additional selective eT/EnT steps. The complexation has been investigated in two solvents of different polarity, that is, CH_2_Cl_2_ and toluene, and a detailed photophysical investigation, supported by electrochemical and theoretical studies, revealed peculiar photoinduced dynamics in the 1:1 supramolecular complex. Parallel eT steps and a rare EnT mechanism that triggers a charge transfer emission have been found to be operative within the self‐assembled structure. Interestingly, these processes are addressed by playing on the solvent polarity and on the photoexcited component.

## 2 Results and Discussion

### 2.1 Binding Studies

**Figure 1:**
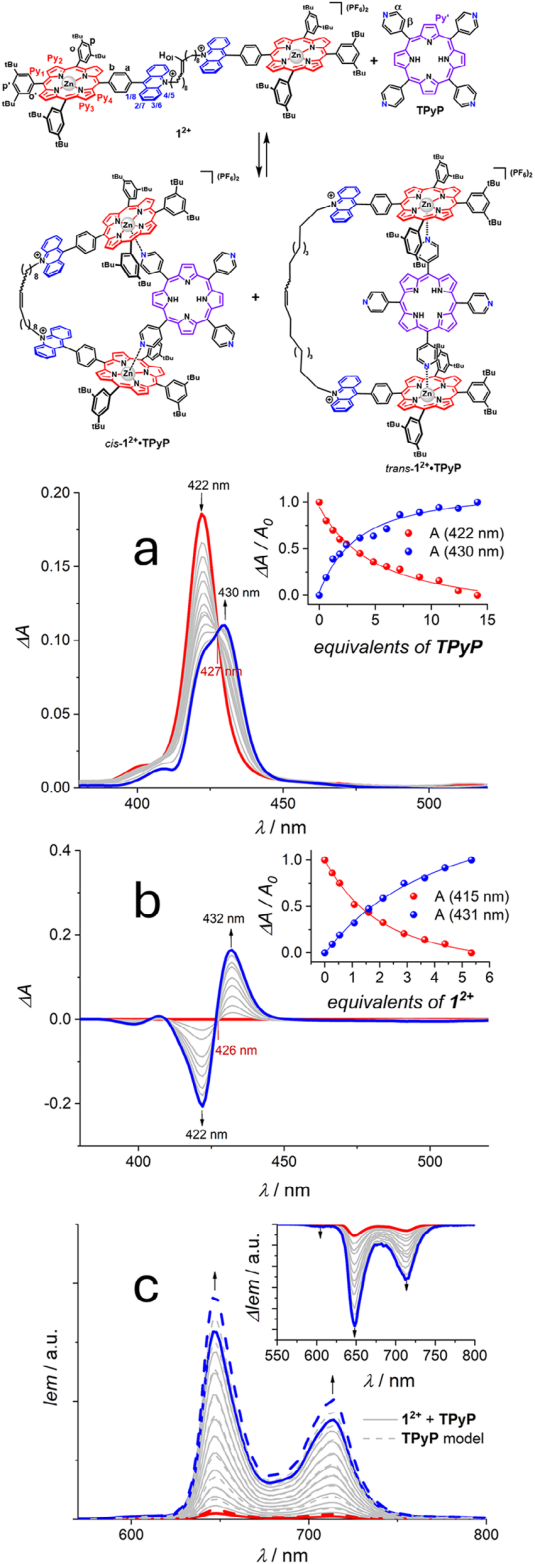
Top: schematic representation of guest complexation in which both *cis* and *trans* forms are represented (for related discussion, see the theoretical section). a) Absorption titration of **1^2+^** (2 × 10^‒7^ mol L^‒1^) with **TPyP** in CH_2_Cl_2_ from 0 (red) to 14 equivalents (blue) after subtraction of the absorption spectra of corresponding reference solutions of **TPyP**. Inset: absolute absorbance variation as a function of the added equivalents of **TPyP** with the relative fitting curves obtained with the Wilcox function. b) Absorption variation upon titration of **TPyP** (5 × 10^−7^ M) with **1^2+^** in CH_2_Cl_2_ from 0 (red) to 5 equivalents (blue), after subtraction of the absorption spectra of corresponding reference solutions of **1^2+^** and **TPyP**. Inset: absolute absorbance variation as a function of the added equivalents of **1^2+^** with the relative fitting curves obtained with the Wilcox function. c) Emission spectra of the solutions in a) (*λ*_exc_ = 427 nm, solid lines) with the spectra of the corresponding reference solutions of **TPyP** (dashed lines) for the sake of comparison. Inset: spectra of the mixtures subtracted by both the spectra of **1^2+^** and **TPyP**.

The association between **1^2+^** and **TPyP** was characterized in CH_2_Cl_2_ and toluene (Figures 1 and S11). When followed by absorption spectroscopy, the interaction between the host and the guest was confirmed by i) a red‐shift of the Soret band (from 422 to 430 nm in CH_2_Cl_2_ and from 425 to 437 nm in toluene) and ii) the presence of an isosbestic point at 427 nm in CH_2_Cl_2_ and at 432 nm in toluene. These spectral changes were observed both when titrating a solution of host with a solution of guest and vice versa (Figures 1a,b and S11a,b). Upon excitation at the isosbestic points, emission titrations revealed that both the already weak fluorescence of the Zn‐porphyrin components in **1^2+^** (bands at 600 and 652 nm) and the fluorescence of the **TPyP** guest (bands at 645 and 712 nm) are substantially quenched upon formation of the complex (Figures 1c and S11c). This unusual outcome indicates that both host and guest undergo a quenching process when involved in the complex. For both titrations, the most meaningful fits were obtained using a 1:1 association model in both solvents (see the Materials and Methods section and Figures S12‐S23). This result can be ascribed to an increased steric hindrance between the porphyrins of two hosts around one **TPyP** guest, thus limiting the formation of the 2:1 complex **[(1^2+^)_2_•TPyP]**. Binding constants (*K*_a_) of 1.2 × 10^6 ^L mol^−1^ in CH_2_Cl_2_ and 3.2 × 10^7^ L mol^−1^ in toluene were estimated by averaging the values obtained from the different elaboration methods (see Section S1 and Figures S12‐S23 for details).

**Figure 2:**
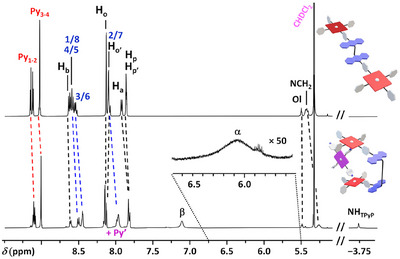
^1^H NMR spectra (500 MHz, CD_2_Cl_2_, 298 K) of **1^2+^** and **1^2+^•TPyP** complex. (For clarity reasons, only the *cis*‐**1^2+^•TPyP** was drawn; see below).

Formation of the 1:1 complex was confirmed by ^1^H NMR spectroscopy in CD_2_Cl_2_ (Figure 2). The presence of the free base was first evidenced by the signal of the tautomeric protons (*δ*_NH_ = ‒3.75 ppm). In addition, the three different aromatic protons of the **TPyP** guest appear as three broad signals (*δ*(H_α_) = 6.07, *δ*(H_β_) = 7.11 and *δ*(H_Py’_) ≈ 7.96 ppm) all integrating for eight protons. This result suggests a dynamic binding since the expected symmetry breaking in a kinetically inert complex should lead to at least two sets of signals for each proton.^[^ ^17^ ^]^ Noteworthy, the unusual chemical shifts observed for H_α_ and H_β_ reflect the influence of the cone of anisotropy of the **ZnTPP** cores on the pyridine rings. However, the protons of **1^2+^** are clearly less affected than the guest (‒0.16 < Δ*δ* < +0.04, Table S3). This observation supports a metal‐ligand interaction and excludes π‐π stacking interactions.

**Figure 3:**
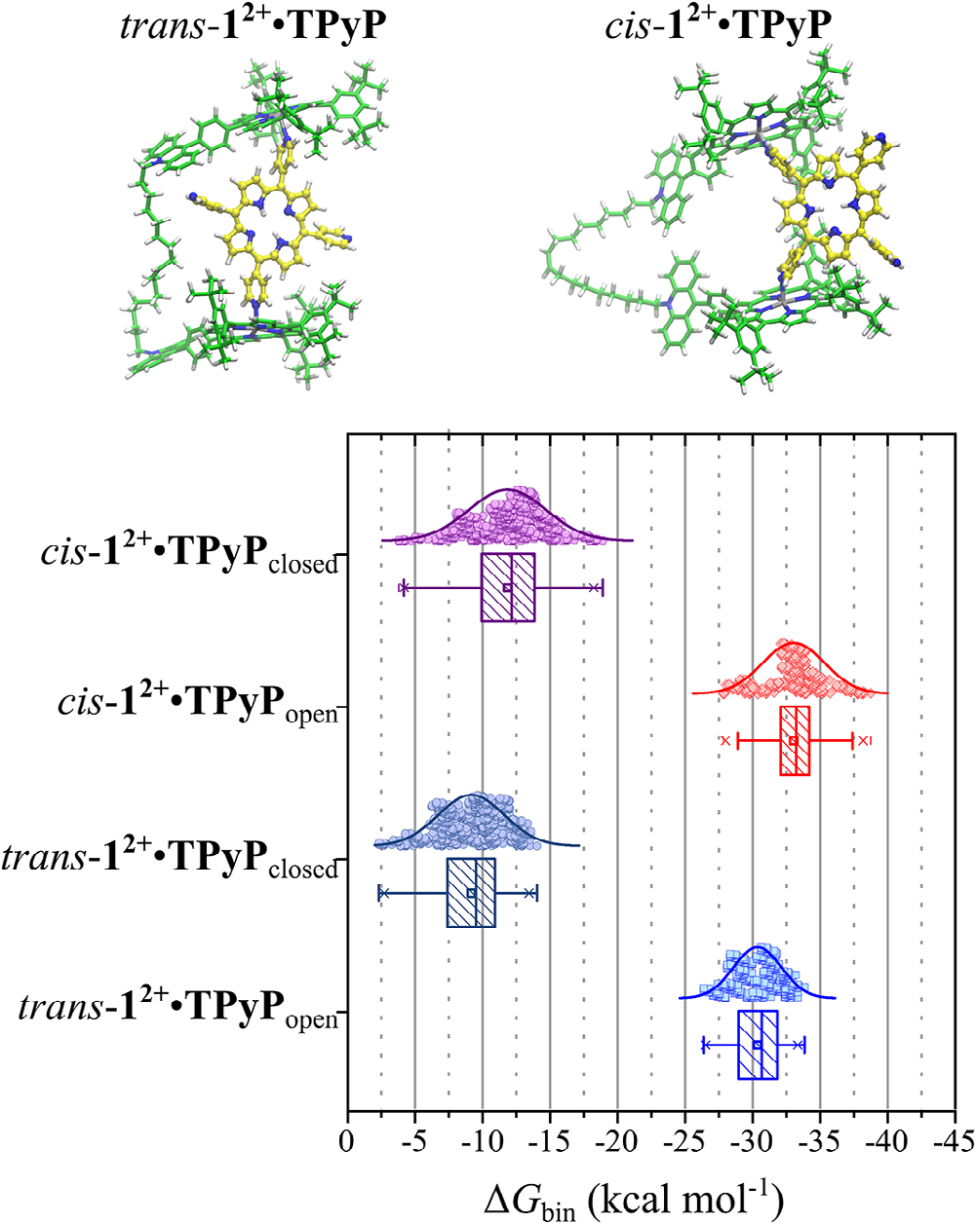
Box charts related to Δ*G*_bin_ for *trans*‐**1^2+^** •**TPyP** and *cis*‐**1^2+^** •**TPyP** calculated by averaging the Gibbs free energy of **1^2+^** in both the open (*trans*‐**1^2+^** •**TPyP**_open_ and *cis*‐**1^2+^** •**TPyP**_open_) and closed conformations (*trans*‐**1^2+^** •**TPyP**_closed_ and *cis*‐**1^2+^** •**TPyP**_closed_). "Open" stands for extended chain, where **ZnTPP** and **Acr** are separated (see Figure S47 in Supporting Information); "closed" stands for folded **1^2+^**, with π–π interaction between **ZnTPP** and **Acr** (see Figure S47 in Supporting Information). Box heights indicate the 25^th^‐75^th^ percentile range, the line inside the box is the median, the small box is the mean value, the line outside the box represents the 5^th^‐95^th^ percentile range. The normal distribution of the calculated Δ*G*_bin_ is also reported.

In order to further investigate the nature of the **1^2+^** •**TPyP** complex, molecular modeling simulations were performed in CH_2_Cl_2_. Two different geometries for the complex were considered, namely i) *cis*‐**1^2+^** •**TPyP**, where two adjacent **TPyP** pyridines are coordinated with both **ZnTPP** cores of **1^2+^** and ii) *trans*‐**1^2+^** •**TPyP**, where two opposite **TPyP** pyridines are coordinated with both **ZnTPP** cores of **1^2+^** (Figure 3). The root mean square deviation (RMSD) plot (Figure S48a, red and blue curves) shows that both **1^2+^** •**TPyP** structures exhibit reduced fluctuation (less than 10 Å) compared to isolated **1^2+^** (Figure S48a, yellow curve), resulting from an increased structural rigidity gained upon complexation. In contrast, the isolated **TPyP** displays minimal RMSD fluctuation (less than 2 Å), due to the low flexibility that characterizes its structure (Figure S48a, dark green curve). **TPyP** remains coordinated to **1^2+^** throughout the simulation, as evidenced by the distance plots between the Zn atom of **1^2+^** and the N atom of **TPyP** ($\bar{d}$ Zn‐μ^1^‐N**_TPyP _** = 2.25 Å) over time (Figure S49a). A similar trend is observed for the distance between the Zn atom of **1^2+^** and the center of mass (com) of **TPyP** ($\bar{d}$ Zn‐**TPyP**_com_ ∼ 9.0 Å) as a function of time (Figure S49b). In both investigated geometries, the combined distribution function (CDF) plot reveals that the region of highest probability is located at distances between Zn and N**_TPyP_** close to 2.3 Å, and at N‐Zn‐N**_TPyP_** angles around 80°, thus indicating the formation of square‐pyramidal coordination complexes for each **ZnTPP** moiety (Figure S49c–f). These results suggest that it might be experimentally challenging to distinguish between the *cis* and *trans* forms of **1^2+^•TPyP**.

Calculation of the binding Gibbs free energies (Δ*G*) between **1^2+^** and **TPyP** (Figure 3) was performed on a selection of the ten most representative conformations (see Materials and Methods in Supporting Information). To achieve a better distribution of Δ*G*, the latter has been calculated by considering the free energies of both conformations of **1^2+^**, open (Δ*G*_O_) and closed (Δ*G*_C_, see Supporting Information). Indeed, RMSD plot versus time of **1^2+^** showed the evolution of the initial open conformation, by folding of the flexible alkene spacer, to a closed conformation stabilized by π−π interactions between one ZnP moiety and an acridinium unit of the tweezer (Figure S47b). The Δ*G*_C_ values were calculated to be −9.2 kcal mol^−1^ (*K*_a_ = 5.2 × 10^6^ L mol^−1^) for the *trans*‐**1^2+^** •**TPyP** complex and −11.8 kcal mol^−1^ (*K*_a_ = 4.8 × 10^8 ^L mol^−1^) for the *cis*‐**1^2+^** •**TPyP** complex. These calculated *K*_a_ values are in good agreement with the experimental one of 1.2 × 10^6^ L mol^−1^. In comparison, the Δ*G*_O_ values obtained using the open conformation of the host are largely overestimated (−30.4 kcal mol^−1^ for *trans*‐**1^2+^** •**TPyP**, *K*_a_ = 1.8 × 10^22^ L mol^−1^ and −33.0 kcal mol^−1^ for *cis*‐**1^2+^** •**TPyP**, *K*_a_ = 1.6 × 10^24^ L mol^−1^). These results suggest that complexation occurs between **TPyP** and the predominant closed conformation of **1^2+^**, representing approximately 85% of the simulation time (Figure S47a). It is worthwhile to note that the Δ*G*_C_ values (2.6 kcal mol^−1^) for the *cis* and *trans* complexes are very similar, thus indicating the possibility of an equilibrium between both geometries in solution.

### 2.2 Electrochemistry

**Figure 4:**
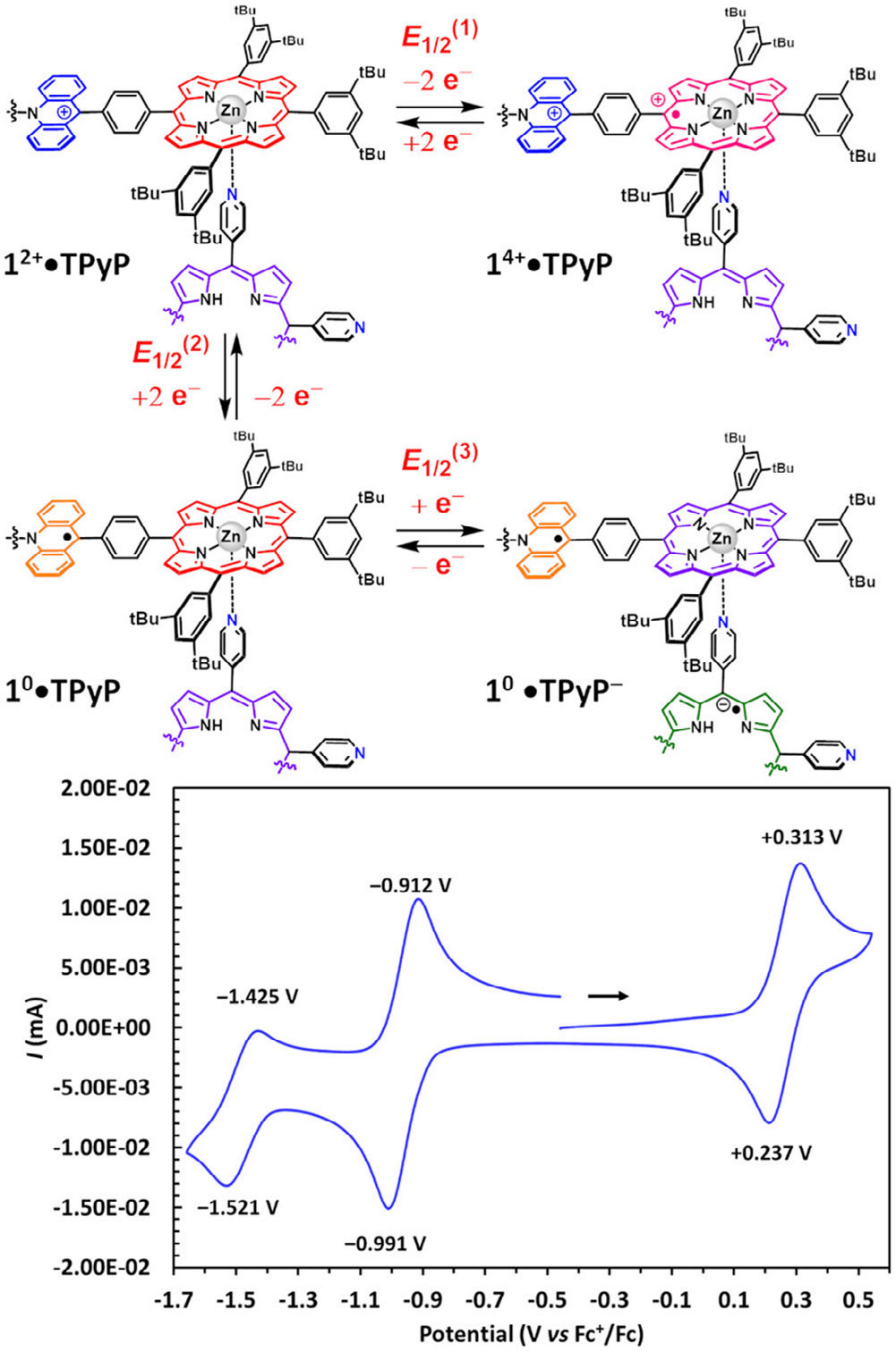
Cyclic voltammograms (C_2_H_4_Cl_2_, WE: Pt, CE: Pt, RE: SCE, 100 mV s^−1^, TBAPF_6_ 0.1 mol L^−1^) of a solution of **1^2+^** •**TPyP** (*c* = 1 × 10^−3^ mol L^−1^). Potentials are referenced against the Fc^+^/Fc couple.

Cyclic voltammetry experiments were made on a preformed 1:1 mixture of **TPyP** and **1^2+^** at a concentration of 1 × 10^−3^ mol L^−1^ (91% complex formation in solution). Three quasi‐reversible redox processes (75 < Δ*E* < 100 mV, Figure 4 and Table S4), namely one in the anodic and two in the cathodic regime, were monitored for **1^2+^** •**TPyP**.^[^ ^18^ ^]^ The first oxidation (*E*_1/2_^(1)^ = +0.275 V vs. Fc^+^/Fc) and reduction waves (*E*_1/2_^(2)^ = ‒0.952 V vs. Fc^+^/Fc) were respectively assigned to the oxidation of both Zn(II) porphyrin cores and the reduction of both acridinium cores of the receptor **1^2+^** in the complex. The third event (*E*_1/2_^(3)^ = −1.473 V vs. Fc^+^/Fc) was associated with the reduction of the complexed **TPyP** ligand.

**Figure 5:**
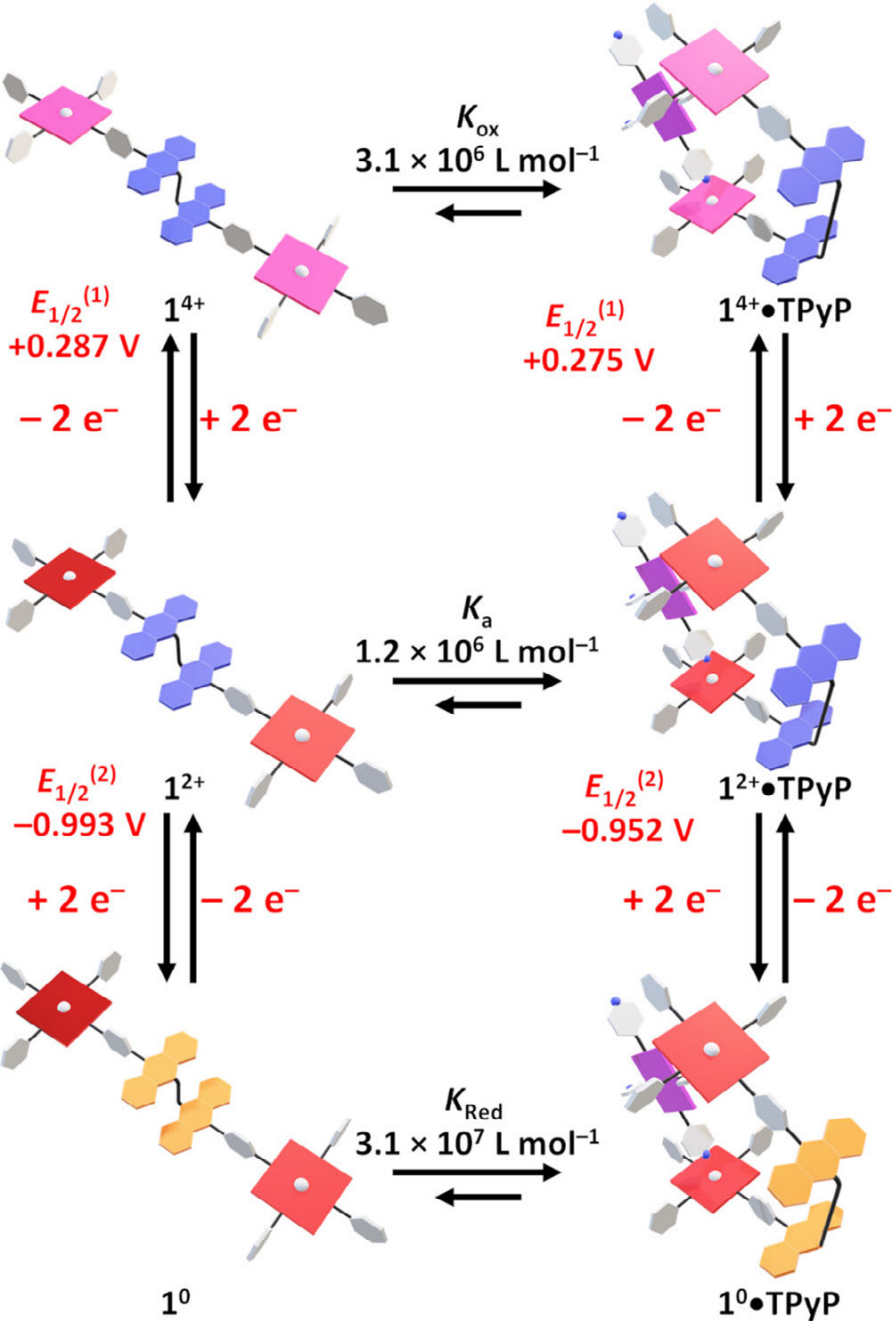
Square scheme analysis leading to the estimation of a binding constant for **1^4+^** •**TPyP** and **1^0^** •**TPyP** complexes, *K*_Ox_ and *K*_Red_, respectively. Potentials are referenced against Fc^+^/Fc. (For clarity reasons, only the *cis*‐**1^2+^** •**TPyP** was drawn).

When compared to the isolated tweezer **1^2+^**, the first oxidation of the Zn(II) porphyrin cores is cathodically shifted (Δ*E*_1/2_ = −12 mV) upon coordination of **TPyP**. This shift allows the estimation of a binding constant (*K*_ox_) of 3.1 × 10^6^ L mol^−1^ in the **1^4+^** •**TPyP** complex according to a square scheme analysis (Figure 5).^[^ ^19^ ^]^ This good binding constant reflects the stability of the assembly upon oxidation (98% complex present in solution) on account of i) the ditopic coordination of the **TPyP** guest to the Zn(II)‐porphyrin moieties and ii) the easy possible rearrangement of the complex provided by the four pyridyl groups of the **TPyP** ligand. Interestingly, an enhanced binding constant (*K*_Red_) of 3.1 × 10^7^ L mol^−1^ and an anodic shift (Δ*E*_1/2_ = +41 mV) were assessed for the **1^0^** •**TPyP** complex by a second square scheme analysis. These values indicate that the reduction/oxidation of the receptor does not affect much the 1:1 complex speciation at the studied concentrations.

### 2.3 Photophysics of Complex 1^2+^•TPyP

In order to get a deeper insight into the mechanisms of quenching of both host and guest in complex **1^2+^** •**TPyP**, Vis‐NIR transient absorption measurements with femtosecond resolution were performed in the two solvents and with two excitation wavelengths: 565, where the Zn porphyrins of **1^2+^** predominantly absorb (> 70%), and 510 nm, where almost selective excitation of **TPyP** is achieved (Figure S24). Comparison with relevant models (**1^2+^**, **ZnTPP,** and **TPyP**) was also made (see the Supporting Information for details).

### 2.4 Studies in CH_2_Cl_2_

**Figure 6:**
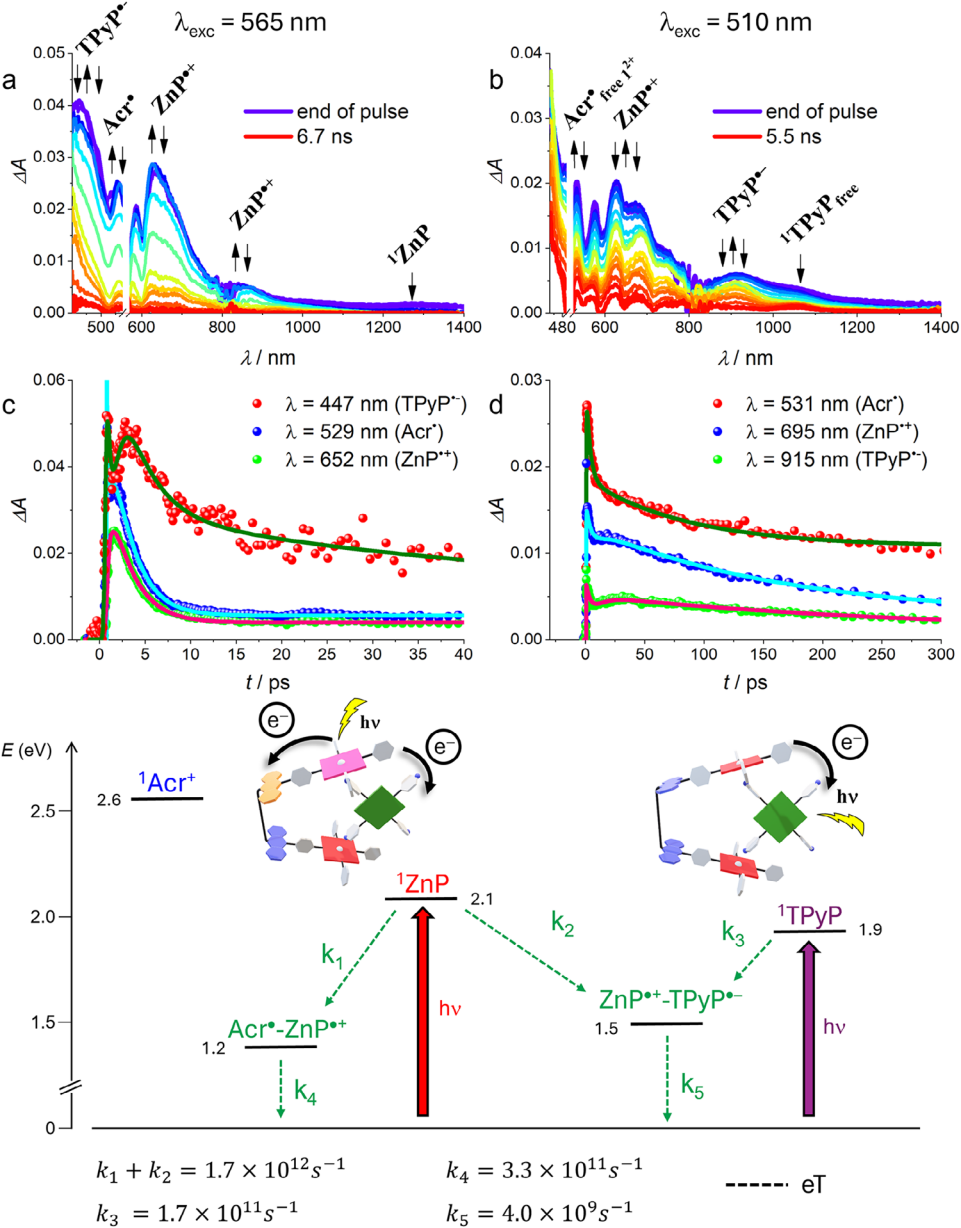
Top: TA spectra of complex **1^2+^•TPyP** in CH_2_Cl_2_ (*E* = 8 µJ/pulse) upon excitation at a) 565 nm and b) 510 nm. Middle: TA kinetics at selected wavelengths with the relative fittings (lines) of **1^2+^•TPyP** upon excitation at c) 565 nm and d) 510 nm. An amplification factor of ca. 1.5 has been applied to some of the kinetic traces for clarity. Bottom: energy level diagram and photoinduced processes of complex **1^2+^•TPyP** in CH_2_Cl_2_.

Upon excitation at 565 nm, formation of the charged species **Acr** ^•^ and **ZnP^•+^** is observed from the typical features of **Acr^•^** (bands at 480 and 520 nm)^[^ ^15^, ^16^, ^20^ ^]^ and **ZnP^•+^** (bands between 600 and 700 nm and at ca. 900 nm) ^[^ ^15^, ^20^, ^21^ ^]^ that appear and decay in 0.6 and 3 ps, respectively (Figure 6a,c). The charge‐separated (CS) state **Acr^•^‐ZnP^•+^** is thus forming and recombining in the complex with fast kinetics, identical to those observed in the uncomplexed **1^2+^** (Figure S52).^[^ ^15^ ^]^ Interestingly, two other bands at around 450 and 930 nm are formed within the same time range, 0.6 ps, and decay with a lifetime of about 250 ps (Figures 6a,c, and S53). They are attributed to the guest radical anion (**TPyP^•−^**), based on spectroelectrochemical data (Figure S50), theoretical simulations (see Supporting Information) and literature reports.^[^ ^22^ ^]^ The observation of the radical anion **TPyP^•−^** supports the coexistence of another (intercomponent) CS state formed by eT from the host to the guest, that is, **ZnP^•+^‐TPyP^•−^**, in addition to the intramolecular one **Acr^•^‐ZnP^•+^**. Notably, this second CS state shows a much slower recombination rate (250 ps).^[^ ^23^, ^24^ ^]^

Following excitation of the complex at 510 nm (almost selective on **TPyP**), the formation and decay of the band of **TPyP^•−^** at 930 nm is clearly evidenced (Figure 6b), even if partially overlapped with the ^1^ **TPyP** signal at 1050 nm (Figure S54a). This band is formed within 6 ps (matching the quenched lifetime of ^1^ **TPyP** inside the complex, Figure S55) and decays in about 250 ps (Figure 6d). The features of the zinc porphyrin radical cation **ZnP^•+^** are also clearly observed, showing a rise of 5–6 ps followed by a decay of ca. 250 ps (Figure 6b, d), that is, the same kinetics of the guest anion **TPyP^•−^**. This indicates that the intercomponent **ZnP^•+^‐TPyP^•−^** CS state forms with a longer time constant (6 ps) upon excitation of the **TPyP** guest, while it decays with the same lifetime.^[^ ^25^ ^].^

A global analysis of the TA maps of **1^2+^•TPyP** for both excitation wavelengths (Figure S56) helps in extracting the profiles of the various species. It is evident that, upon excitation of the Zn‐porphyrin of **1^2+^** (Figure S56a), two CS states are formed, that is, **Acr^•^‐ZnP^•+^** and **ZnP^•+^‐TPyP^•−^**, living 3 and 250 ps, respectively. On the other hand, upon excitation of **TPyP** at 510 nm (Figure S56b) only the formation of one CS species is observed, that is, **ZnP^•+^‐TPyP^•−^**, which lives about 250 ps. The spectrum of the species with a long lifetime is the singlet of the unbound **TPyP**.

Based on the above results, we can rationalize the light‐induced processes of **1^2+^•TPyP** in CH_2_Cl_2_ by means of the energy diagram reported in Figure 6 (bottom). Upon excitation of the porphyrin moieties of **1^2+^**, two parallel eT processes occur from the Zn porphyrin singlet, which generate **Acr^•^‐ZnP^•+^** and **ZnP^•+^‐TPyP^•−^**. The lifetime of the ^1^ **ZnP** is quenched to 0.6 ps and both CS states form with the same kinetics. Eventually, they recombine to the ground state with different lifetimes, the longer‐lived one being **ZnP^•+^‐TPyP^•−^**, likely due to the supramolecular versus covalent binding. Notably, parallel eT processes are only rarely found in the literature and are always related to systems where porphyrins are coupled to strong electron acceptors of different nature.^[^ ^2^, ^4^, ^26^ ^]^ On the other hand, when excitation is addressed to **TPyP**, only the **ZnP^•+^‐TPyP^•−^** species is formed in about 6 ps, then recombining with the same lifetime of ca. 250 ps. It can be noticed that we found no experimental evidence of the generation of **Acr^•^‐ZnP^•+^** from **ZnP^•+^‐TPyP^•−^**, via **TPyP^•−^** → **Acr^+^** charge shift, although the process appears to be thermodynamically allowed.

### 2.5 Studies in Toluene

**Figure 7:**
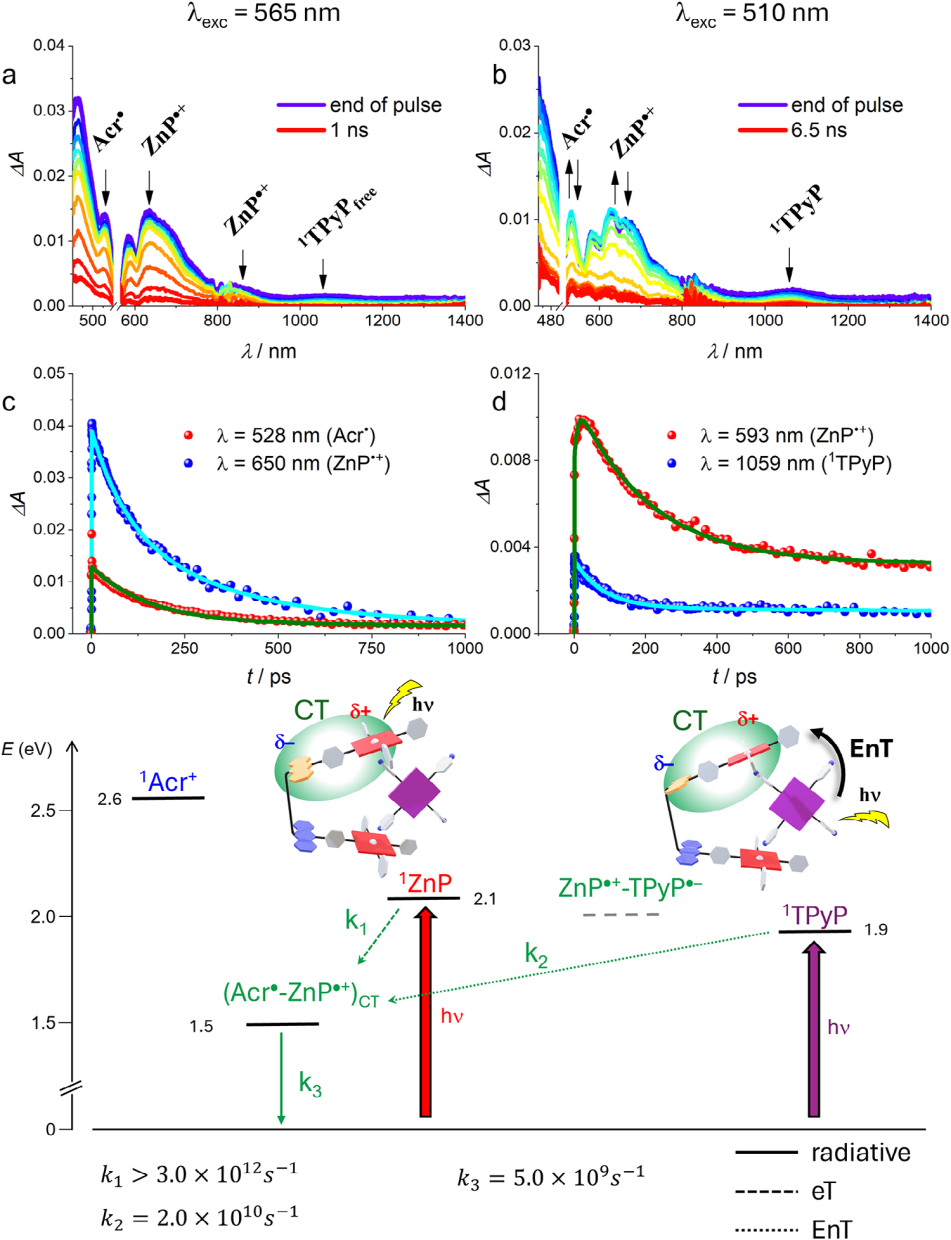
Top: TA spectra of complex **1^2+^•TPyP** in toluene (*E* = 8 µJ/pulse) upon excitation at a) 565 nm and b) 510 nm. Middle: TA kinetics at selected wavelengths with the relative fittings (lines) of **1^2+^•TPyP** upon excitation at c) 565 nm and d) 510 nm. An amplification of ca. 2.5 has been applied to some of the kinetic traces for clarity. Bottom: energy level diagram and photoinduced processes of complex **1^2+^•TPyP** in toluene.

Upon prevalent excitation of **1^2+^** in **1^2+^•TPyP** at 565 nm, only the features of **Acr^•^** and **ZnP^•+^** are detected, while the bands of the **TPyP^•−^** radical anion at 450 and 930 nm were not observed (Figure 7a). Accordingly, no eT between the host and the guest was found to take place. The **Acr^•^** / **ZnP^•+^** bands decay with a lifetime of 200 ps and no rise was detected (Figures 7c and S59), indicating that the **ZnP** → **Acr^+^** eT within **1^2+^** in the adduct becomes ultrafast, while charge recombination remains in the order of 200 ps as in free **1^2+^** (Figure S58b).^[^ ^15^ ^]^ This is compatible with the weak CT emission detected in the NIR region (Figure S63a). It can be noticed that a small fraction (8%) of free components **1^2+^** and **TPyP** accounts for the bands observed at 430 and 1300 nm (^1^ **ZnP**) and 1070 nm (^1^ **TPyP**).

Under selective excitation of **TPyP** at 510 nm in **1^2+^•TPyP** (Figure 7b), the ^1^ **TPyP** band at 1070 nm has a double exponential decay, with lifetimes of ca. 60 ps and more than 7 ns (Figures 7d and S62). The shorter component, which is also coherent with the rise (ca. 50 ps) of the **ZnP^•+^** radical cation bands (Figure 7d), suggests an EnT from ^1^ **TPyP** to the intramolecular charge transfer (CT) state of **1^2+^**, typical of this solvent (8% of free **TPyP** occurring in solution is responsible for the longer component). This (**Acr^•^**‐**ZnP^•+^**)_CT_ state exhibits the same transient spectral features of the CS state **Acr^•^**‐**ZnP^•+^** (lifetime ca. 200 ps), despite the likely lower extent of charge separation with respect to more polar media, including CH_2_Cl_2_ (see above). Further evidence of the occurrence of this EnT process lies in the excitation spectrum of the complex recorded at 1000 nm, where only the CT emission is present (Figure S63b). In this spectrum, both the absorption features of **1^2+^** (acridinium and Zn‐porphyrin) and the first Q band of **TPyP** guest at 510 nm are detected. Remarkably, an EnT process from a singlet to a CT state is a very rarely observed phenomenon.^[^ ^8c^ ^].^

An energy level diagram (Figure 7, bottom) summarizes the processes occurring in toluene upon light excitation. Addressing light to the individual components of **1^2+^** brings to the population of the emissive intramolecular CT state (**Acr^•^**‐**ZnP^•+^**)_CT_. Likewise, also the excitation of the singlet state of the **TPyP** guest populates this level via EnT.^[^ ^27^ ^]^ Since the value taken for the radius of the porphyrins strongly affects the calculated energy of **ZnP^•+^‐TPyP^•−^** state with the Weller equation, the experimental uncertainty is large. It is thus difficult to conclude whether this state lies above or below the **TPyP** singlet. Since no evidence of **ZnP** → **TPyP** eT is found when exciting ^1^ **TPyP**, the relatively high energy gap between ^1^ **TPyP** and (**Acr^•^**‐**ZnP^•+^**)_CT_ might favor the EnT between those two states.

## 3 Conclusion

It has been demonstrated that a bis(acridinium‐Zn(II)porphyrin) host efficiently encapsulates a photoactive guest, namely tetrapyridylporphyrin, leading to a peculiar photophysical behavior of the formed adduct. The photophysics of the system is strongly affected by the polarity of the solvent and the moiety selectively addressed, making possible a fine tuning of the photoinduced processes. A polar environment, such as CH_2_Cl_2_, favors the occurrence of two eT within the supramolecular assembly: an intercomponent eT between the porphyrin moiety and the **TPyP** guest in parallel with an intramolecular one between **ZnP** and **Acr^+^**. On the other hand, a less polar solvent, such as toluene, hinders the intercomponent eT process while providing a route for EnT from the singlet state of the **TPyP** guest to the charge transfer ^1^(**Acr^•^**‐**ZnP^•+^**)_CT_ state of the host.

Unusual photoinduced processes that are rarely observed separately, such as parallel eTs and a singlet‐to‐CT‐state energy transfer, are recorded here in a single system by the combination of multi‐responsive building blocks, that is, Zn(II) porphyrins, acridinium moieties, and a free‐base porphyrin. The presence of many orthogonal stimuli that can trigger different photophysical behaviors makes such complex supramolecular assemblies of potential interest in the domains of information processing and storage networks,^[^ ^15^ ^]^ molecular wires and switches, photoactive components for transistors, chemical sensors, and photodiodes.^[^ ^20c^ ^]^

## Supporting Information

Additional experimental details, materials and methods, absorption, emission and transient absorption spectra, electrochemical and photophysical data, DFT calculations. The authors have cited additional references within the Supporting Information.^[^ ^15^, ^20^, ^21^, ^28^ ^]^

## Conflict of Interest

The authors declare no conflict of interest.

## Supporting information



Supporting Information

## Data Availability

The data that support the findings of this study are available from the corresponding author upon reasonable request.
