# Supplementary material for: Path Selective Photoinduced Energy and Electron Transfer in a Bis(acridinium‐Zn(II) Porphyrin)‐tetrapyridyl Porphyrin Host‐Guest Complex
Source: Chemistry. 2025 Sep 19;32(4):e02604. doi: 10.1002/chem.202502604 (PMC12840847; doi:10.1002/chem.202502604)
Supplement: Supplementary file 1 — Supporting Information [file CHEM-32-e02604-s001.pdf]

Supporting Information

for

Path selective photoinduced energy and electron transfer in a bis(acridinium-Zn(II) porphyrin)-tetrapyridyl porphyrin host-guest complex

Federica Ruani, Daniele Veclani, Daniel Sanchez-Resa, Amy Edo-Osagie, Geordie Creste, Andrea Barbieri, Valérie Heitz,* Henri-Pierre Jacquot de Rouville,* Nicola Armaroli,* Barbara Ventura*

Table of contents

Section S1. Materials and Methods 2
- Absorption and emission spectroscopy, photophysics 2
- Binding studies 3
- Free energy (ΔG_{CS}) calculations for electron transfer 3
- Computational studies 4
  - Reduction of **TPyP** and analysis of absorption properties. 4
  - Dynamic properties and binding Gibbs free energies of **1**$^{2+}$, **TPyP**, and **1**$^{2+}$**•TPyP** models 5
- Electrochemistry and spectroelectrochemistry 6

Section S2. Supplementary Data and Results 7
- Additional photophysical data in CH_2Cl_2 7
- Photophysical characterization of 1^{2+} in toluene 8
- Binding studies 14
- Electrochemistry of complex **1**$^{2+}$**·TPyP** in toluene 21
  - Formation of the **1**$^{2+}$**·TPyP** complex 21
- Modelling 31
  - Reduction of **TPyP** and analysis of the UV-VIS absorption properties 31
  - Dynamic properties 36
- Spectroelectrochemical measurements 40
- Additional photophysical data for complex **1**$^{2+}$**·TPyP** in CH_2Cl_2 41
- Additional photophysical data for complex **1**$^{2+}$**·TPyP** in toluene 44

References 48

Section S1. Materials and Methods

Absorption and emission spectroscopy, photophysics

Spectroscopic grade Uvasol® CH_2Cl_2 from Merck was treated with dry $CaCO_3$ before use, to eliminate traces of acid. Spectroscopic grade Uvasol® methanol and toluene from Merck were used without further purification. All standards used were acquired from commercial suppliers and used without further purification: *meso*-tetraphenylporphyrin from Porphychem and Coumarin 153 from Sigma Aldrich. Zinc-tetraphenylporphyrin (**ZnTPP**) was purchased from Porphychem and further purified with column chromatography. 5,10,15,20-Tetra(4-pyridyl)porphyrin (**TPyP**) guest was purchased from Sigma Aldrich and used without further purification.

Absorption spectra were recorded with Perkin–Elmer Lambda 650 UV-vis or Lambda 950 UV-vis-NIR spectrophotometers in 1 cm or 2 mm quartz cuvettes. Emission spectra were collected with an Edinburgh FLS920 spectrofluorometer, equipped with a Peltier-cooled Hamamatsu R928 PMT (280–850 nm) and with an Edinburgh FLS920 fluorimeter equipped with a Hamamatsu R5509-72 InP/InGaAs photomultiplier tube supercooled at 193 K in a liquid nitrogen cooled housing and a TM300 emission monochromator with a NIR grating blazed at 1000 nm (400-1700 nm).

Fluorescence quantum yields (Φ_{fl}) were evaluated with the comparative method developed by Demas and Crosby,[1] upon correction of the spectra for the wavelength-dependent photomultiplier response.

Air-equilibrated references used were *meso*-tetraphenylporphyrin (TPP) in toluene (Φ_{fl} = 0.11)[2] for the porphyrin components and Coumarin 153 in ethanol (Φ_{fl} = 0.544)[3] for the acridinium moieties. Measurements at 77K were performed using Pyrex tubes dipped in liquid nitrogen in a quartz Dewar. Excitation spectra were corrected for the wavelength-dependent lamp intensity.

Emission lifetimes in the nanosecond range were determined by using an IBH time-correlated single-photon counting apparatus with nanoLED excitation sources at 560 nm, 465 nm and 368 nm.

Pump-probe transient absorption measurements were performed with an Ultrafast Systems HELIOS (HE-VIS-NIR) femtosecond transient absorption spectrometer by using, as excitation source, a Newport Spectra Physics Solstice-F-1K-230 V laser system, combined with a TOPAS Prime (TPR-TOPAS-F) optical parametric amplifier (pulse width: 100 fs, 1 kHz repetition rate) tuned at 510 nm and 565 nm. Two sapphire crystals for continuum generation in the visible range (450-800 nm) and in the NIR (800-1600 nm) have been employed. The overall time resolution of the system is 300 fs. Air-equilibrated solutions in 0.2 cm optical path cells were analyzed under continuous stirring. The pump energy on the samples was 8 μJ/pulse. For **TPyP** model the pump energy at 510 nm was reduced to 2.8 μJ/pulse to reduce photodegradation. Surface Xplorer V4 software from Ultrafast Systems was used for data acquisition and analysis. The 3D data surfaces were corrected for the chirp of the probe pulse prior to analysis. The solutions of the complexes were prepared by mixing $\mathbf{1}^{2+}$ and **TPyP** at equal concentrations (1×10^{-5} in CH_2Cl_2 and 5×10^{-6} in toluene), ensuring a high percentage of formed complex: 75% in CH_2Cl_2 and 92% in toluene. Model solutions of $\mathbf{1}^{2+}$ and **TPyP** were prepared with concentrations as in the mixtures while **ZnTPP** solutions were prepared to have A_{565} ca. the A of tweezer (7.6×10^{-6} M in CH_2Cl_2 and 3.2×10^{-6} M in toluene).

Estimated errors are 10% on transient absorbance lifetimes, 10% for luminescence lifetimes, 10% for molar absorption coefficients and 10% on quantum yields.

Binding studies

Several absorption and emission titration experiments were performed for each solvent, including titrations performed with the host (**1^{2+}**) as the titrate and the guest (**TPyP**) as the titrant, and vice versa. Stock solutions of titrate were about 10^{-7} M, while those of the titrant were of the highest concentrations allowed by solubility limitations (toluene: 6×10^{-6} M for **TPyP**, 3×10^{-5} M for **1^{2+}**; CH_2Cl_2: 6×10^{-5} M for **TPyP**, 4×10^{-5} for **1^{2+}**). The titrations were performed by incremental addition of micro aliquots of the stock solution of the titrant to a solution of the titrate, keeping the final added volume below 10% of the total volume, to avoid significant dilution of the titrate.

Titration data were elaborated with ReactLab equilibria and Supramolecular.org software or fitted with equation [1], valid for 1:1 stoichiometry:[4]

$$y = y_0 + \left\{\frac{\Delta y}{2 * S_0} \cdot \left[k_d + x + S_0 - \sqrt[2]{(k_d + x + S_0)^2 - 4x \cdot S_0}\right]\right\} \quad (1)$$

where y is any measurable signal (absorbance or emission intensity); y_0 is the onset (signal from the free component); Δy is the maximum spectral difference with respect to the free component; S_0 is the initial concentration of the titrate; x is the variable concentration of the titrant; k_d is the dissociation constant (i.e., the inverse of the association constant).

In the case of fitting with equation [1] or Supramolecular.org, absorption spectra of the mixtures were priorly elaborated by subtracting the absorption contribution of **TPyP**, which is not expected to change upon complexation with **1^{2+}**, at each titration point. Moreover, when **TPyP** is titrated with **1^{2+}**, also the absorption spectrum of the latter has been subtracted from that of the mixture at each titration point.[4]

A comparison between the output data obtained from the three different methods allowed the estimation of the binding constants.

Free energy (ΔG_{CS}) calculations for electron transfer

The free energy for charge-separation (ΔG_{CS}) leading to $ZnP^{\bullet+}$-$TPyP^{\bullet-}$ has been calculated in CH_2Cl_2 and toluene through the semi-empirical Rehm-Weller equation [2]:[5]

$$\Delta G_{CS} = e[E^{ox} - E^{red}] - E_{00}(ZnP) - \frac{e^2}{4\pi\varepsilon_0\varepsilon_S d} - \frac{e^2}{8\pi\varepsilon_0}\left(\frac{1}{r^+} + \frac{1}{r^-}\right)\left(\frac{1}{\varepsilon_r} - \frac{1}{\varepsilon_S}\right) \quad (2)$$

where E_{ox} and E_{red} are, respectively, the oxidation potential of the porphyrin unit (+0.75 V *vs* SCE, measured in $C_2H_4Cl_2$)[6] and the reduction potential of the **TPyP** unit (-0.92 V *vs* SCE, measured in CH_2Cl_2); E_{00} *(ZnP)* is the energy of the singlet excited state of the porphyrin (2.1 eV)[6]; e is the elementary charge (1.602×10^{-19} C); ε_0 is the vacuum permittivity (8.854×10^{-12} Fm^{-1}); ε_s is the permittivity of the solvent in which the free energy must be calculated (in our case $\varepsilon_s = 2.38$ for toluene and $\varepsilon_s = 8.93$ for CH_2Cl_2); ε_r is the permittivity of the solvent in which the electrochemical experiments have been performed; d is the distance between the donor (D) and the acceptor (A); r^+ and r^- are the radii of the oxidized and reduced species. The redox potential of **ZnTPP** and **TPyP** has been measured in different solvents ($C_2H_4Cl_2$ and CH_2Cl_2, respectively), so there is not a unique ε_r. Accordingly, we have taken $\varepsilon_r = \varepsilon_s$ for the calculation of ΔG_{CS} in CH_2Cl_2 (thus eliminating the last term of the equation), since the dielectric constants are similar ($\varepsilon = 8.93$ for CH_2Cl_2 and $\varepsilon = 10.36$ for $C_2H_4Cl_2$), and ε_r as an average of the two constants ($\varepsilon_r = 9.65$) for the calculation in toluene.

The values for d, r^+ and r^- (9 Å, 5 Å and 5 Å,[7] respectively) have been obtained by approximating the donor and acceptor units as spheres, taking the radii as a distance between the center and the periphery of the molecular units and the (D-A) separation as a distance between the two centroids. The structures for these calculations have been obtained with GFN2-xTB.

As an estimation of the total reorganization energy ($\lambda = \lambda_i + \lambda_s$) for the charge-separation process, the solvent contribution (λ_s) has been calculated according to the Born–Hush equation (3), while the internal reorganization term (λ_i) has been considered negligible.

$$\lambda_s = \frac{e^2}{4\pi\varepsilon_0}\left(\frac{1}{2r_+} + \frac{1}{2r_+} - \frac{1}{d}\right)\left(\frac{1}{n^2} - \frac{1}{\varepsilon_s}\right) \quad (3)$$

n is the refractive index of the solvent in which the reorganization energy is calculated.

Computational studies

Different computational approaches have been used to:

- Obtain the reduction properties and the absorption features of **TPyP** and **TPyP$^-$**;
- Study the dynamic properties of **1^{2+}**, **TPyP**, and the **1^{2+}•TPyP** complex in order to determine the binding Gibbs free energies (ΔG).

*Reduction of **TPyP** and analysis of absorption properties.*

Density functional theory (DFT) calculations were performed to investigate both the reduction behavior and the photophysics of **TPyP**. All simulations were carried out using the 5.0 version of the Gaussian16 program package,[8] in combination with the WB97X-D range-separated functional functional.[9] Valence triple-zeta basis set with polarization functions, def-TZVP, was used for all atoms.[10]

The one-electron reduction proprieties were obtained using the thermodynamic cycle for the Gibbs free energy reported in scheme S1.[11]

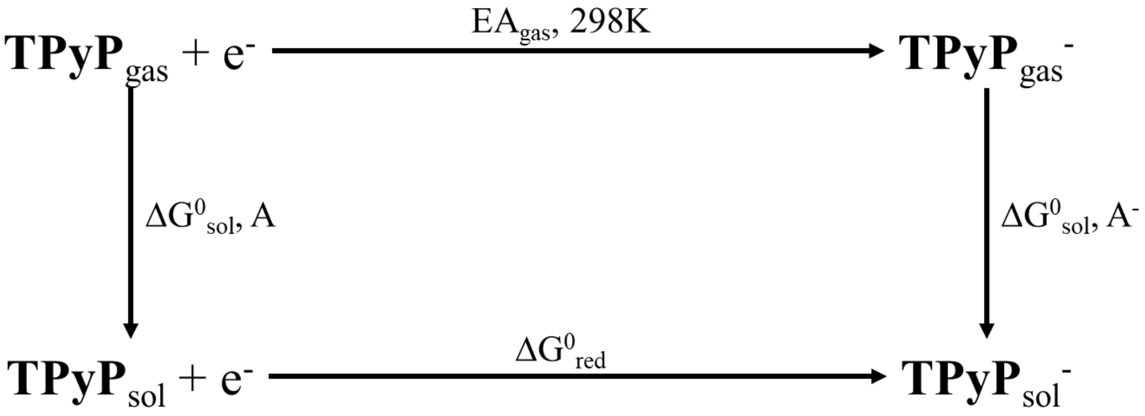

*Scheme S1: Thermodynamic cycle for the Gibbs free energy of the reduction reaction of **TPyP**.*

The electron affinity EA_{gas} is defined as the enthalpy change between the neutral and anionic molecule in the gas phase at 298 K and 1 atm, where the enthalpy is calculated using the total electronic energy (E_{Elec}), the zero-point vibrational energy (E_{ZPVE}) and thermal corrections (E_{therm} 0→298 K). The free energy of the reduction reactions (ΔG^0_{red}) can be calculated through:

$\Delta G^0_{red} = EA_{gas} + \Delta G_{sol,A^-} - \Delta G_{sol,A}$

where $\Delta G_{sol,A}$ and $\Delta G_{sol,A^-}$ denote the free energy of solvation of the neutral and anionic states of the molecule, respectively. Then, the E_{red} can be evaluated by Faraday's law:

$$E_{red} = \frac{-\Delta G^0_{red}}{nF}$$

where n is the number of electrons transferred, and F is the Faraday constant.

To obtain the aforementioned thermodynamic cycle, geometric optimizations were initially performed in the gas phase, frequency calculations were always used to confirm the nature of stationary point found by geometry optimizations. To account for the solvent effect, single-point calculations were executed on the minimum gas-phase energy structures using the implicit solvation polarizable continuum model (PCM).[12] The one-electron reduction potentials were obtained in CH_2Cl_2 and toluene environment.

The ground-state properties and absorption behavior of the neutral form of **TPyP** were investigated using spin-restricted calculations while reduced anionic species (doublets state) were optimized using a spin-unrestricted approach. Geometry optimizations were carried out at WB97X-D/def-TZVP level directly using the implicit PCM solvent model (CH_2Cl_2). To investigate the nature of the absorption transitions, time dependent DFT formalism (TD-DFT)[13] was employed at the same level of theory used for geometry optimizations. The first 20 singlet excitations were calculated and their nature was assessed with the support of Natural Transition Orbital (NTO) analysis.[14]

Dynamic properties and binding Gibbs free energies of ***1***$^{2+}$*,* ***TPyP****, and* ***1***$^{2+}$***•TPyP*** *models*

The dynamic properties and ΔG of **1**$^{2+}$, **TPyP**, and *cis*-**1**$^{2+}$**•TPyP** and *trans*-**1**$^{2+}$**•TPyP** were investigated using the extended tight-binding GFN2-xTB method,[15] implemented in the xTB software version 6.6.1.[16] GFN2-xTB is a semi-empirical method that utilizes empirical parameters to reduce computational costs. It is a variant of the Density Functional Tight Binding (DFTB)[17] approach, where the Kohn–Sham energy is expanded in terms of density fluctuations relative to a superposition of atomic reference densities. Key features of GFN2-xTB include the use of a minimal valence basis set consisting of atom-centered Gaussian functions. The GFN2-xTB Hamiltonian accounts for electrostatic interactions and exchange-correlation effects up to second order in the multipole expansion. Notably, in this method, parameterization relies solely on global and element-specific parameters, with no pairwise parameters employed, in contrast to the traditional DFTB approach.[15] This method has been applied to the dynamics of various types of systems, delivering excellent results.[18]

Molecular dynamics (MD) simulations were performed on the **1**$^{2+}$, **TPyP**, *cis*-**1**$^{2+}$**•TPyP**, and *trans*-**1**$^{2+}$**•TPyP** models to capture a range of binding Gibbs free energies (ΔG) from different snapshots, using the implicit solvent Analytical Linearized Poisson-Boltzmann (ALPB) method in CH_2Cl_2[19] without periodic boundary conditions. Initially, geometry optimization was conducted with extreme convergence criteria for energies and gradients to generate the starting structures for the MD simulations. After optimization, the systems were pre-equilibrated using MD simulations in the NVT ensemble with a Berendsen thermostat[20] at 298.15 K for 50 ps, then thermalized at 500 K for 50 ps, and finally equilibrated again at 298.15 K for 100 ps, using a timestep of 4 fs. C-H bonds, with

fictitious hydrogen masses of 4 amu, were constrained at their optimized lengths using the SHAKE algorithm.[21] The simulation was then run in the NVT ensemble at 298.15 K for 2.5 ns with a timestep of 4 fs. Trajectory analyses were performed using Visual Molecular Dynamics (VMD) software version 1.9.3[22], combined distribution functions were prepared using TRAVIS software,[23] and non-covalent interactions were analyzed using IGMPlot software version 3.8.[24]

Given the complexity of the conformational energy landscape of the host-guest complex, ΔG_b values were obtained from the 10 most representative geometries (based on root mean square displacement) of **1**$^{2+}$ (both open and closer structures), **TPyP**, and *cis*-**1**$^{2+}$**•TPyP** and *trans*-**1**$^{2+}$**•TPyP**, extrapolated from the MD simulations. These structures were further optimized at the GFN2-xTB level, followed by Hessian calculations to confirm the final energy was a true minimum (i.e., all vibrational frequencies were positive). The ΔG was calculated as the difference between the free energies of *cis*-**1**$^{2+}$**•TPyP** or *trans*-**1**$^{2+}$**•TPyP** and the individual **1**$^{2+}$ (both open and closer structures) and **TPyP** molecules, each at their respective conformational minimum:

$$\Delta G_O = G_{\mathbf{1\bullet TPyP}} - (G_{\mathbf{1\_open}} + G_{\mathbf{TPyP}})$$

$$\Delta G_C = G_{\mathbf{1\bullet TPyP}} - (G_{\mathbf{1\_closed}} + G_{\mathbf{TPyP}})$$

Where ΔG_O is the binding Gibbs free energy concerning the average of the Gibbs free energies for the open conformation of **1**$^{2+}$ ($G_{\mathbf{1\_open}}$), ΔG_C is the binding Gibbs free energy regarding the average of the Gibbs free energies for the closed conformation of **1**$^{2+}$ ($G_{\mathbf{1\_closed}}$). The Gibbs free energies (G) of the optimized geometries were computed as the sum of the electronic energy (E), including D4 dispersion correction, thermochemical corrections (G_{RRHOT}) following the rigid-rotor-harmonic-oscillator model, and the solvation contribution (G_{solv}) calculated using the ALPB implicit solvation model (CH_2Cl_2).

Electrochemistry and spectroelectrochemistry

Electrochemical experiments were acquired on a Biologic SP-150 potentiostat. Cyclic voltammetry experiments were performed using a Pt working electrode (0.071 cm^2). The electrode surface was polished routinely with 0.05 μm alumina-water slurry on a felt surface immediately before use. The counter electrode was a Pt coil and the reference electrode was calomel (Hg/Hg_2Cl_2/saturated KCl). Samples were prepared in CH_3CN (Aldrich, anhydrous, 99.8%) using 0.1 mol L^{-1} tetrabutylammonium hexafluorophosphate ($TBAPF_6$, Sigma Aldrich, +99%) as supporting electrolyte.

Spectroelectrochemical measurements were performed on a CH_2Cl_2 solution with concentration 7×10^{-5} M of **TPyP**. The in-situ UV-vis-NIR spectroelectrochemical measurements were carried out with a *Metrohm Autolab* PGSTAT204 potentiostat/galvanostat and an Avantes AvaSpec-ULS2048CL-EVO-RS CCD detector with a 50 μm slit, equipped with an Avantes AvaLight-DHc light source in a thin layer quartz glass spectroelectrochemical cell (*ALS*, model SEC-C). A platinum gauze was used as the working electrode, a platinum wire was the counterelectrode while the reference electrode was made by Ag/Ag^+ in $AgNO_3$ 0.01 M in acetonitrile (*ALS*, model RE-7). Tetrabutylammonium hexafluophosphate ($TBAPF_6$) 0.1 M in CH_2Cl_2 was used as the electrolyte. All the solutions were deaerated for 15 min in argon and the cuvette was sealed for maintaining the argon atmosphere during the experiments. The spectroelectrochemical tests were carried out at room temperature.

Section S2. Supplementary Data and Results

Additional photophysical data in CH_2Cl_2

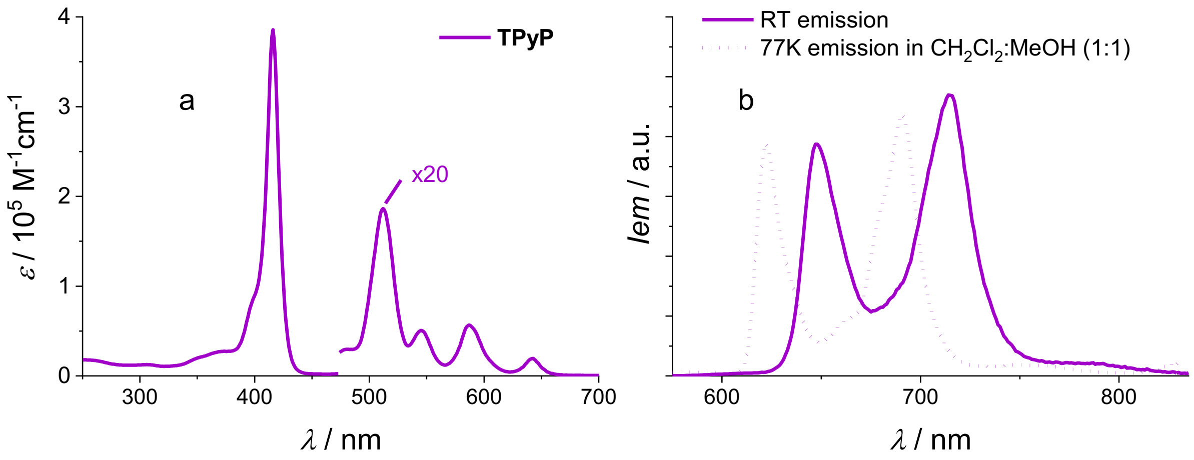

*Fig. S1: Absorption (a) and emission (λ_{exc} = 513 nm) (b) spectra of **TPyP** at RT in CH_2Cl_2 (continuous) and at 77K in CH_2Cl_2:MeOH (1:1) (dotted).*

*Table S1: Emission data for reference compounds **ZnTPP**, **Acr**, **TPyP** and tweezer $\mathbf{1}^{2+}$ at RT in CH_2Cl_2 and at 77K in CH_2Cl_2:MeOH (1:1).*

		RT, CH_2Cl_2			77K, CH_2Cl_2:MeOH (1:1)		
		λ_{max} (nm)	$\Phi_{em}{}^{a}$	τ^{b} (ns)	λ_{max} (nm)	τ^{b} (ns)	E^{c} (eV)
**ZnTPP**[6]	^{1}ZnTPP	600; 650	0.039	1.8	598; 657	2.3	2.1
	^{3}ZnTPP	-	-	-	787	19.6×10^6	1.6
**Acr**[6]	^{1}Acr	510	0.11	2.2	473; 506; 536	18.1	2.6
**1**$^{2+}$[6]	Acr-^{1}ZnP	600; 650	<10^{-4}	-	606; 664	1.8	2.0
	Acr-^{3}ZnP	-	-	-	801	16.9×10^6	1.5
	^{1}Acr-ZnP	500	<10^{-4}	-	-	-	-
**TPyP**	^{1}TPyP	648; 714	0.055	7.6	642; 711	11.4	1.9

^{a}Fluorescence quantum yields, measured with reference to **TPP** (*meso*-tetraphenylporphyrin) in aerated toluene as a standard for the porphyrin units and with reference to Coumarin 153 in ethanol for the acridinium units. ^{b} Fluorescence and phosphorescence lifetimes, excitation at 560 and 465 nm for porphyrin units and at 368 nm for acridinium moieties. ^{c} Energy of the excited state determined as the energy of the 0–0 emission band collected at 77 K.

Photophysical characterization of 1^{2+} in toluene

The full photophysical characterization of $\mathbf{1}^{2+}$ in CH_2Cl_2 has been presented in a previous work.[6] The key steady-state absorption and emission properties in toluene (mostly reported here for the first time) are collected in Fig. S2-S10 and Table S1. In both CH_2Cl_2[6] and toluene, the ground state absorption spectrum of $\mathbf{1}^{2+}$ evidences charge-transfer (CT) absorption features (Fig. S2 and Fig. S3), due to the presence of a donor (Zn porphyrin) and an acceptor (acridinium) unit. In the excited state, transient absorption features indicating the presence of the **Acr$^{\bullet}$-ZnP$^{\bullet+}$** species are evidenced in both solvents (see ref [6] and the discussion in the main text). On the other hand, a broad luminescence band is also observed in the NIR region, but only in toluene. This observation may underpin a less extensive charge separation (i.e., CT *vs.* CS character) of $\mathbf{1}^{2+}$ in the less polar solvent, leading to a peculiar emission feature of CT character.[6] Alternatively, in CH_2Cl_2, the lowest-lying level, possibly exhibiting a larger degree of intramolecular charge separation compared to toluene, might be non-emissive, due to the energy-gap law.

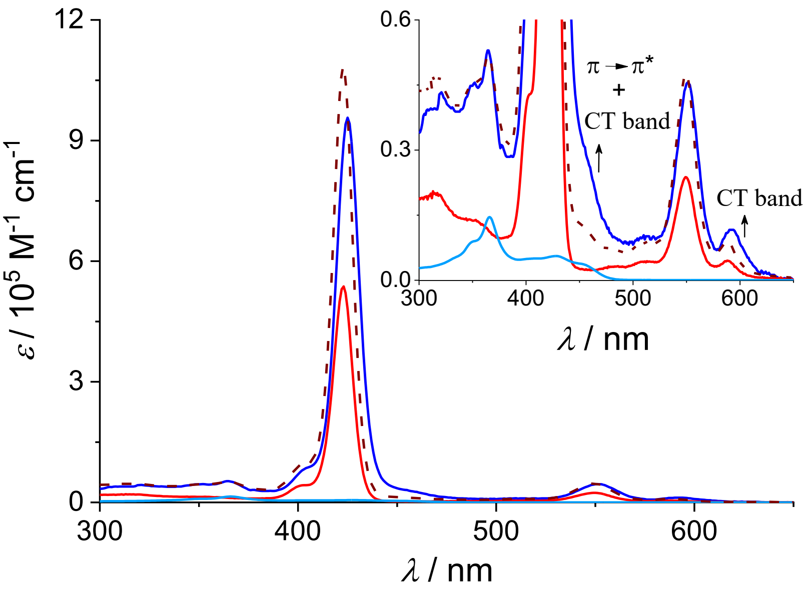

*Fig. S2: Absorption spectra in toluene of **1**$^{2+}$ (blue), **ZnTPP** (red) and **Acr** (cyan), along with a simulated spectrum (brown dashed) given by the weighted sum of the individual components. Inset: amplification of the spectra in the 300-650 nm range.*

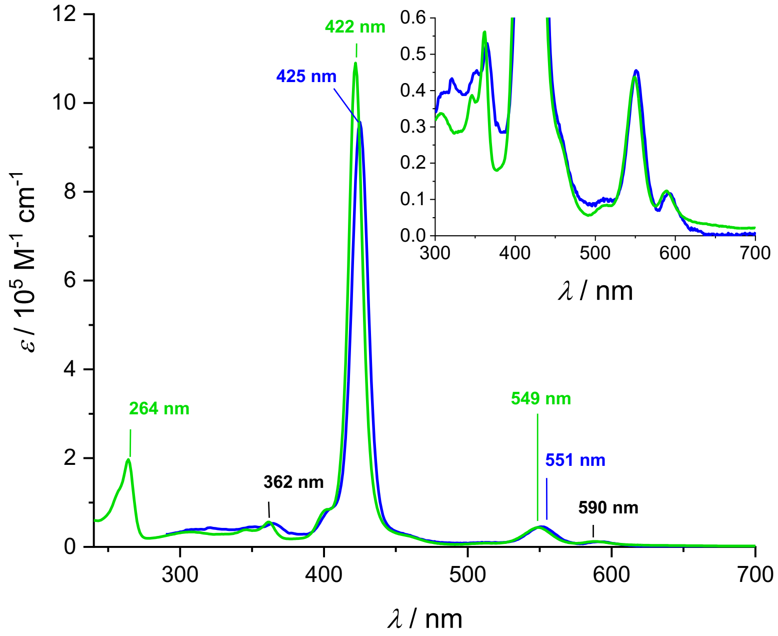

Fig. S3: Comparison between the molar absorption coefficient of ***1***$^{2+}$ *in* CH_2Cl_2 *(green) and in toluene (blue).*

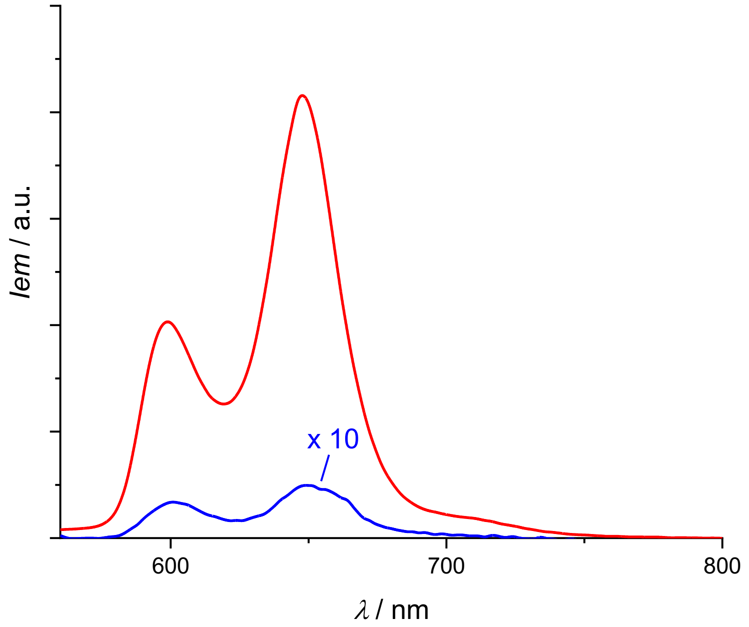

Fig. S4: Corrected luminescence spectra of isoabsorbing solutions of ***ZnTPP*** *(red) and* ***1***$^{2+}$ *(blue) in toluene;* λ_{exc} *= 552 nm (selective on ZnP). The figure shows the > 99% quenching of the Zn-porphyrin emission in* ***1***$^{2+}$*.*

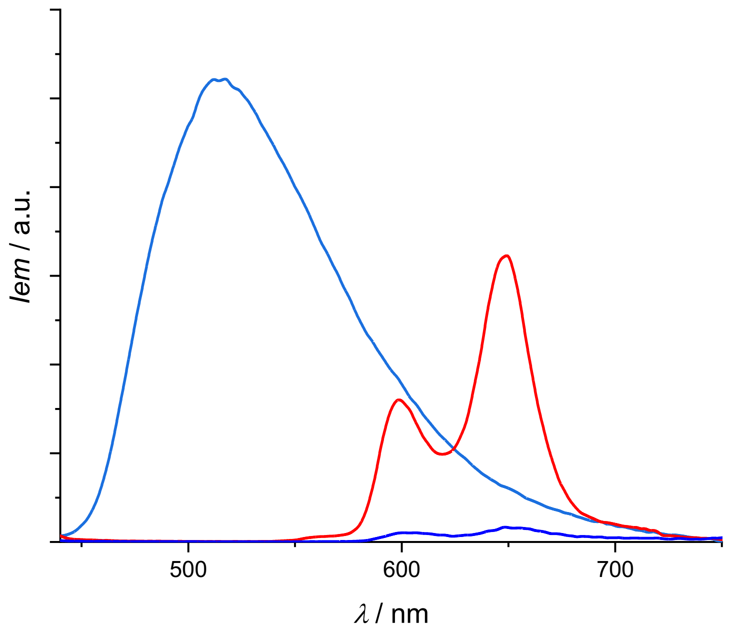

*Fig. S5: Corrected luminescence spectra in toluene of **Acr** (cyan), **ZnTPP** (red) and **1**$^{2+}$ (blue), upon predominant excitation of the acridinium moiety (λ_{exc} = 365 nm, in which acridinium absorption accounts for 57% of the total). Solutions of the models were prepared in order to have the same number of photons absorbed by the corresponding tweezer moieties. From these data it can be concluded that both the acridinium and the Zn-porphyrin units are almost completely quenched in **1**$^{2+}$.*

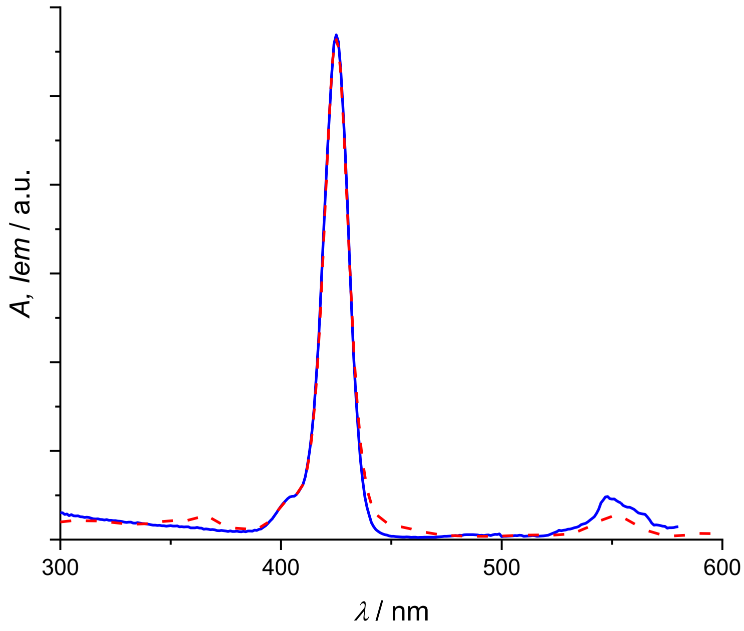

*Fig. S6: Corrected excitation spectrum of **1**$^{2+}$ (blue) in toluene recorded at λ_{em} = 600 nm (Zn-porphyrin emission). The arbitrarily scaled absorption spectrum is also reported for the sake of comparison (red dashed). It can be observed that the bands of the acridinium units (350-360 nm) are not present in the excitation spectrum, indicating the absence of an energy transfer process from the acridinium to the Zn-porphyrin in **1**$^{2+}$.*

In a previous paper[6] we showed that, in CH_2Cl_2 at 77 K, the steady-state emission bands of ZnP and Acr moieties of **1**$^{2+}$ are completely recovered, indicating that intramolecular electron transfer is prevented in frozen matrix. Here we show that in toluene at low temperature the CT emission is still detected, but strongly blue-shifted (λ_{max} = 689 nm at 77 K compared to λ_{max} = 856 nm at RT, Fig. S7 and Fig. S8), due to the hampered solvent reorganization. The excitation spectrum of this band (λ_{em} = 674 nm, Fig. S9) matches the absorption features of both ZnP and Acr moieties, suggesting an energy transfer from both ^{1}Acr and ^{1}ZnP to the emissive CT state, in toluene at 77 K. Notably, in toluene, **1**$^{2+}$ exhibits also the phosphorescence band of **ZnTPP**, but red-shifted compared to **ZnTPP** alone (Fig. S10), as it was found also in CH_2Cl_2.[6]

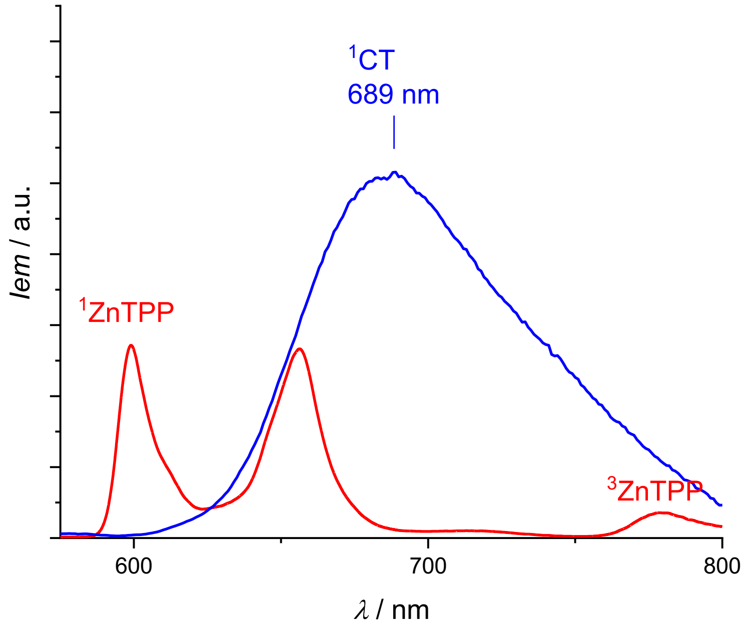

*Fig. S7: Corrected luminescence spectra at 77 K in toluene of isoabsorbing solutions of **ZnTPP** (red) and **1**$^{2+}$ (blue), upon selective excitation of the ZnP moiety (λ_{exc} = 551 nm).*

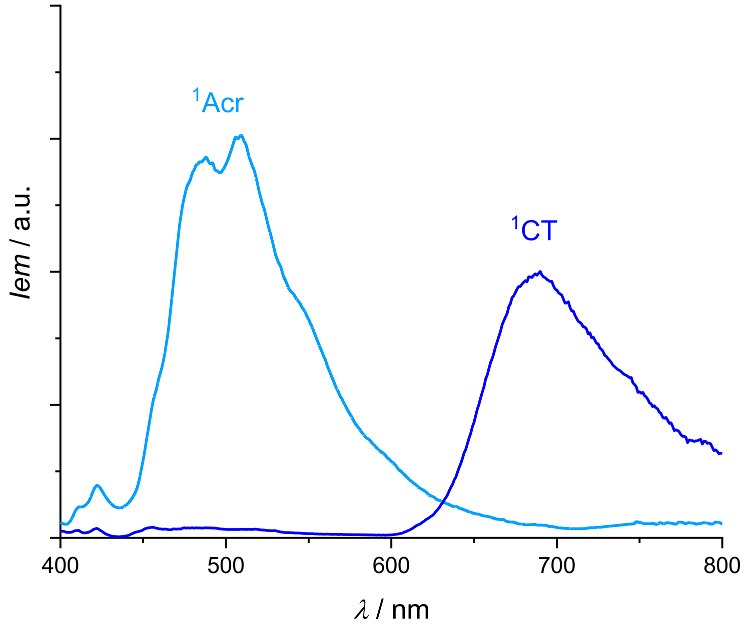

*Fig. S8: Corrected luminescence spectra at 77 K in toluene of isoabsorbing solutions of **Acr** (cyan) and **1**$^{2+}$ (blue) upon prevalent excitation of the **Acr** moiety (λ_{exc} = 365 nm).*

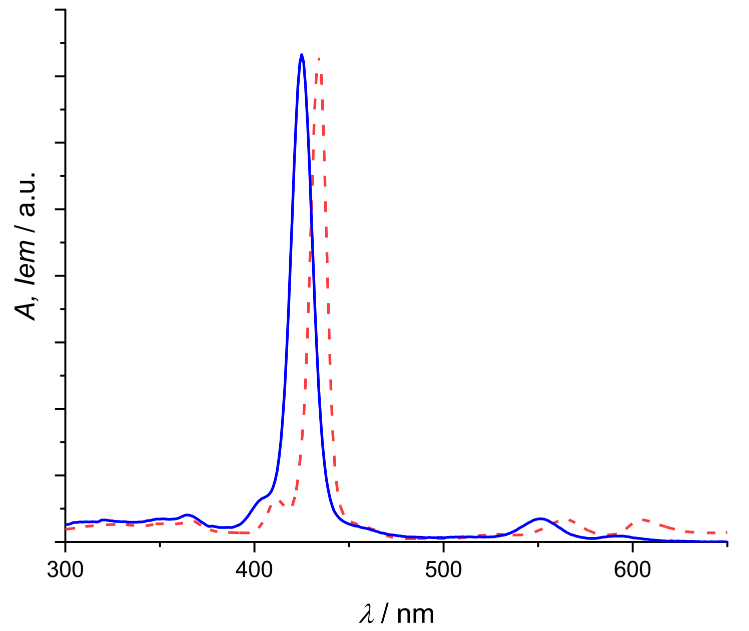

Fig. S9: Corrected excitation spectrum of ***1***$^{2+}$ *at 77 K in toluene (red dashed line,* λ_{em} *= 674 nm). Blue line: RT arbitrarily scaled absorption spectrum for the sake of comparison.*

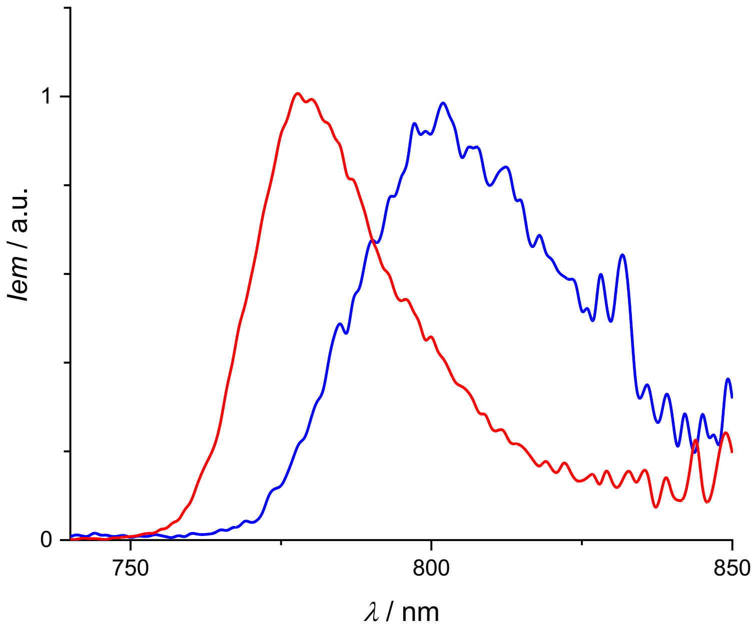

Fig. S10: Normalized corrected time-gated phosphorescence spectra at 77 K in toluene of ***ZnTPP*** *(red) and* ***1***$^{2+}$ *(blue) upon selective excitation of the ZnP moiety (*λ_{exc} *= 551 nm).*

*Table S2: Emission data for reference compounds **ZnTPP**, **Acr**, **TPyP** and tweezer **1**$^{2+}$ at RT and at 77 K in toluene.*

		RT			77 K		
		λ_{max} (nm)	$\Phi_{em}{}^{a}$	τ^{b} (ns)	$\lambda_{max}{}^{em}$ (nm)	τ^{b} (ns)	E^{c} (eV)
**ZnTPP**[25]	^{1}ZnTPP	594, 642	0.047	1.9	598, 654	2.7	2.07
	^{3}ZnTPP	-	-	-	780	28.3×10^{6}	1.59
**Acr**	^{1}Acr	517	0.070	2.2 (88%); 4.6 (12%)	488; 509	19.0	2.54
**1**$^{2+}$	Acr-^{1}ZnP	600, 652	9×10^{-4}	-			
	^{1}(Acr$^{\bullet}$-ZnP$^{\bullet+}$)$_{CT}$	856	-	-	689	-	1.80^{d}
	Acr-^{3}ZnP	-	-	-	802	21.4×10^{6}	1.55
	^{1}Acr-ZnP	518	4×10^{-4}	-	488; 518	-	2.54
**TPyP**[7]	^{1}TPyP	645; 712	0.085	9.4	636; 697; 706	10.6	1.95

^{a} Fluorescence quantum yields, measured *vs*. **ZnTPP** (zinc-*meso*-tetraphenylporphyrin) in aerated toluene as standard for the porphyrin units, and *vs*. Coumarin 153 in ethanol, as standard for acridinium moieties. ^{b} Fluorescence and phosphorescence lifetimes, excitation at 465 nm for porphyrin units and at 368 nm for acridinium moieties. ^{c} Energy of the excited states determined as the energy of the 0–0 emission band collected at 77 K. ^{d} The reported value is calculated from the 77 K emission maximum (λ_{max} = 689 nm); it can be noticed that at room temperature the emission maximum is strongly red shifted (λ_{max} = 856 nm, which corresponds to E = 1.45 eV[6]).

Binding studies

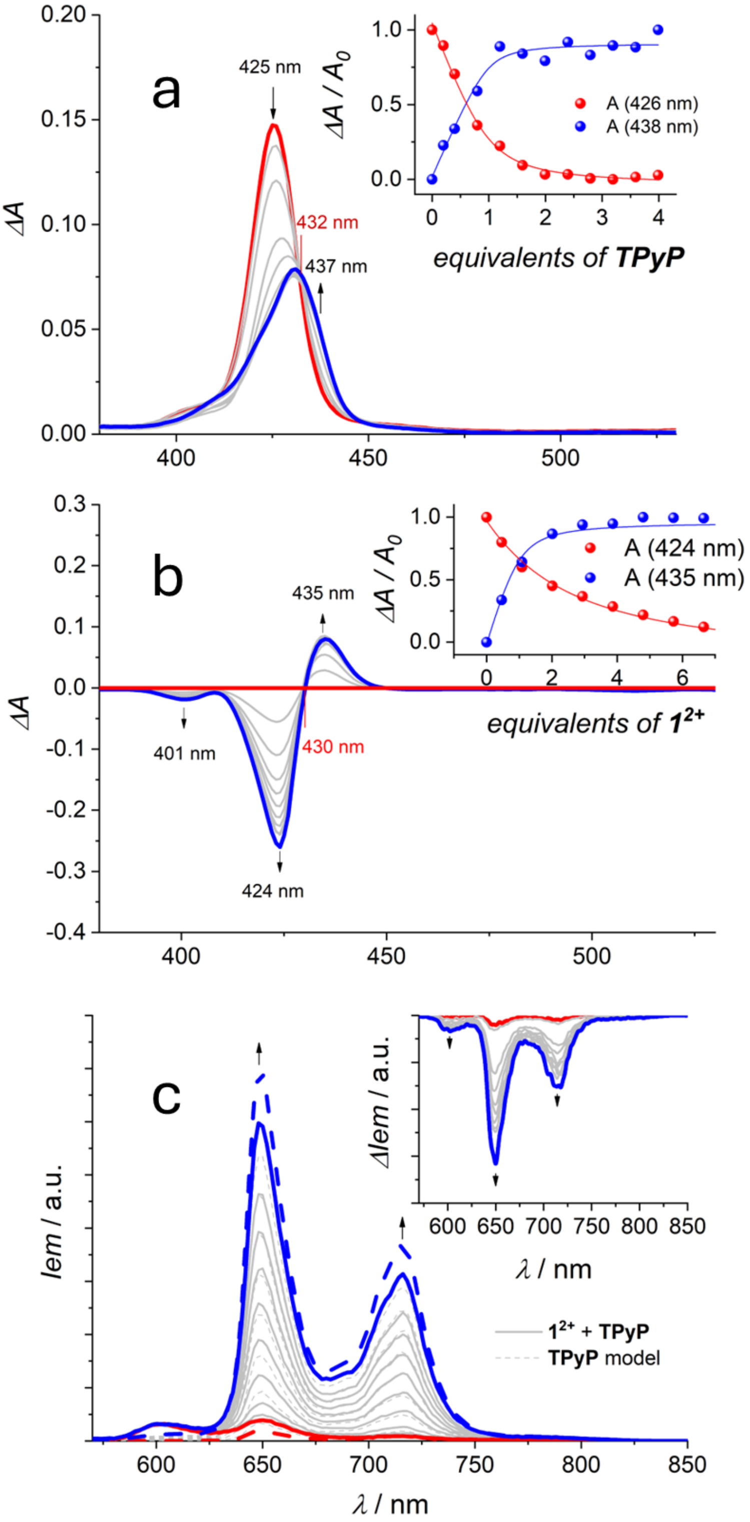

Fig. S11: a) Absorption titration of $\mathbf{1}^{2+}$ *(2×10⁻⁷ M) with* ***TPyP*** *in toluene from 0 (red) to 4 equivalents (blue) after subtraction of the absorption spectra of corresponding reference solutions of* ***TPyP****. Inset: absolute absorbance variation as a function of the added equivalents of* ***TPyP*** *with the relative fitting curves obtained with the Wilcox function. b) Absorption variation upon titration of* ***TPyP*** *(4×10⁻⁷ M) with* $\mathbf{1}^{2+}$ *in toluene from 0 (red) to 6 equivalents (blue) after subtraction of the absorption spectra of corresponding reference solutions of* $\mathbf{1}^{2+}$ *and* ***TPyP****. Inset: absolute absorbance variation as a function of the added equivalents of* $\mathbf{1}^{2+}$ *with the relative fitting curves obtained with the Wilcox function. c) Emission spectra of the solutions in a) (*λ_{exc} *= 427 nm, solid lines) with the spectra of the corresponding reference*

*solutions of **TPyP** (dashed lines) for the sake of comparison. Inset: spectra of the mixtures subtracted by both the spectra of **1**$^{2+}$ and **TPyP**.*

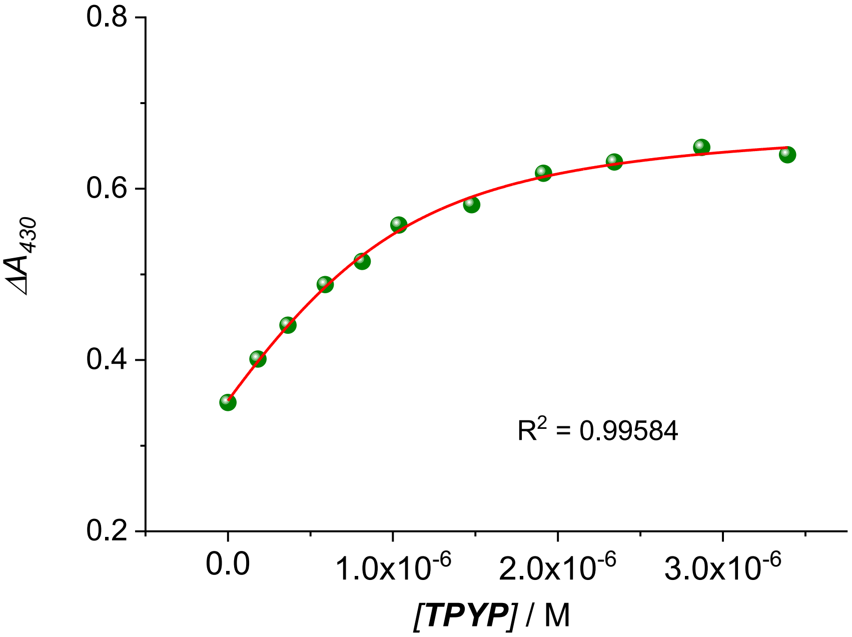

*Fig. S12: Changes in ΔA at 430 nm for titration of **1**$^{2+}$ with **TPyP** in CH_2Cl_2. The fitting according to equation [1] is also reported.*

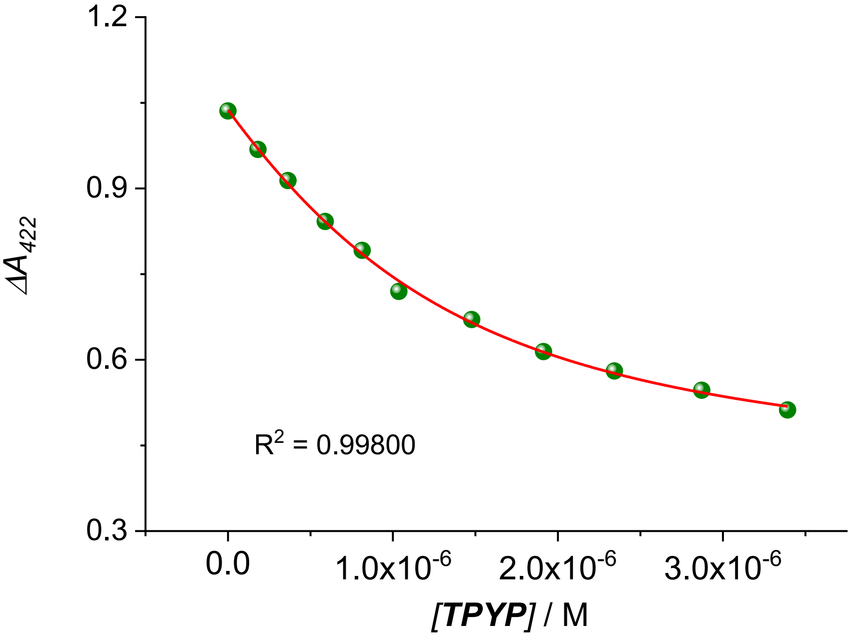

*Fig. S13: Changes in ΔA at 422 nm for titration of **1**$^{2+}$ with **TPyP** in CH_2Cl_2. The fitting according to equation [1] is also reported.*

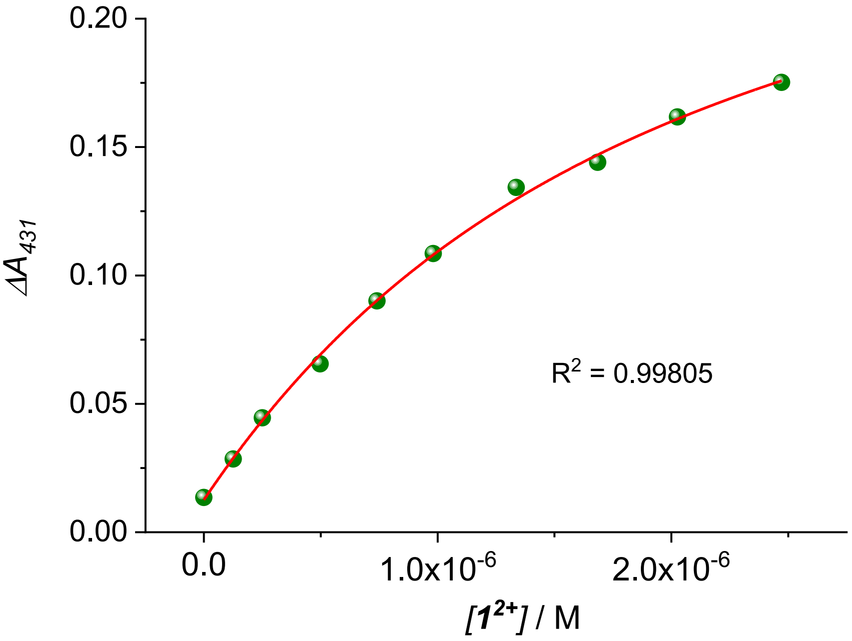

Fig. S14: Changes in ΔA at 431 nm for titration of ***TPyP*** *with* ***1***$^{2+}$ *in* CH_2Cl_2*. The fitting according to equation [1] is also reported.*

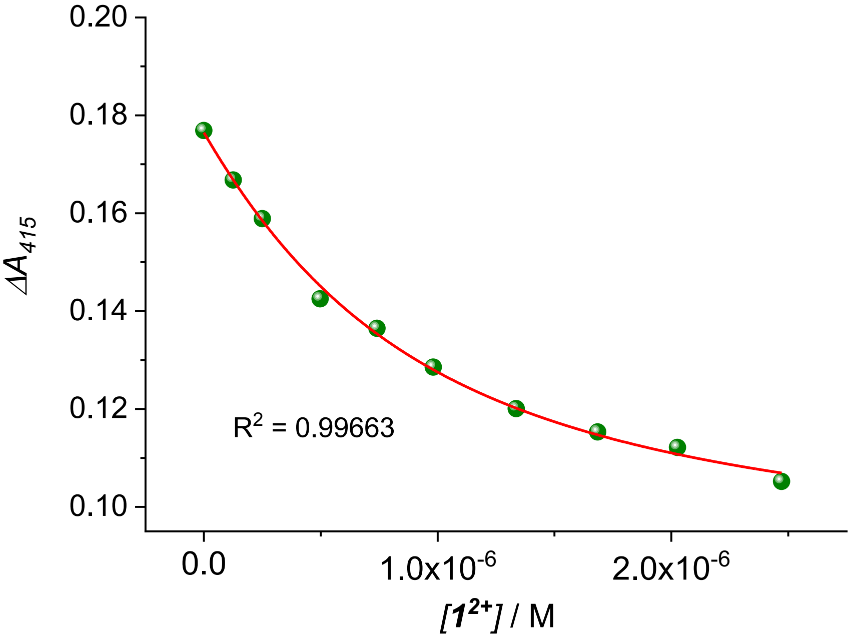

Fig. S15: Changes in ΔA at 415 nm for titration of ***TPyP*** *with* ***1***$^{2+}$ *in* CH_2Cl_2*. The fitting according to equation [1] is also reported.*

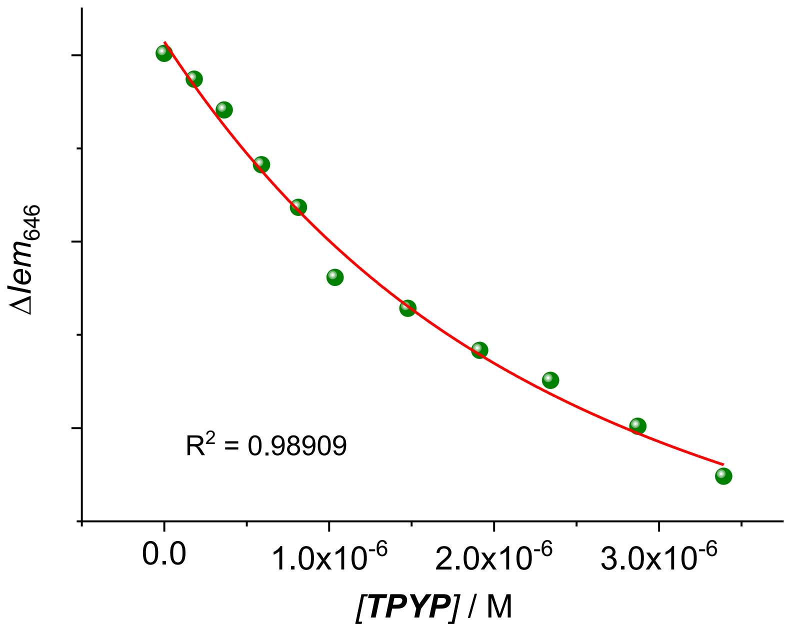

Fig. S16: Changes in ΔIem at 646 nm for titration of ***1***$^{2+}$ *with* ***TPyP*** *in* CH_2Cl_2*. The fitting according to equation [1] is also reported.*

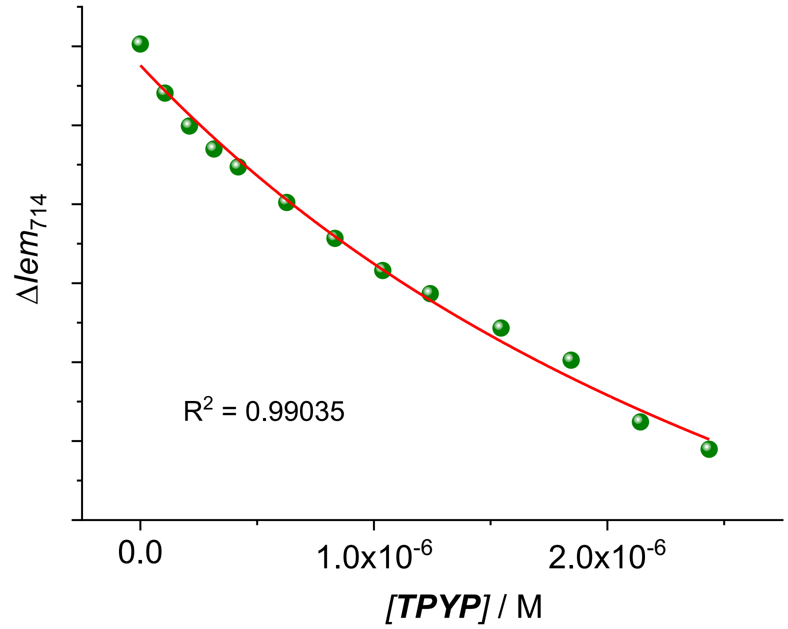

Fig. S17: Changes in ΔIem at 714 nm for titration of ***1***$^{2+}$ *with* ***TPyP*** *in* CH_2Cl_2*. The fitting according to equation [1] is also reported.*

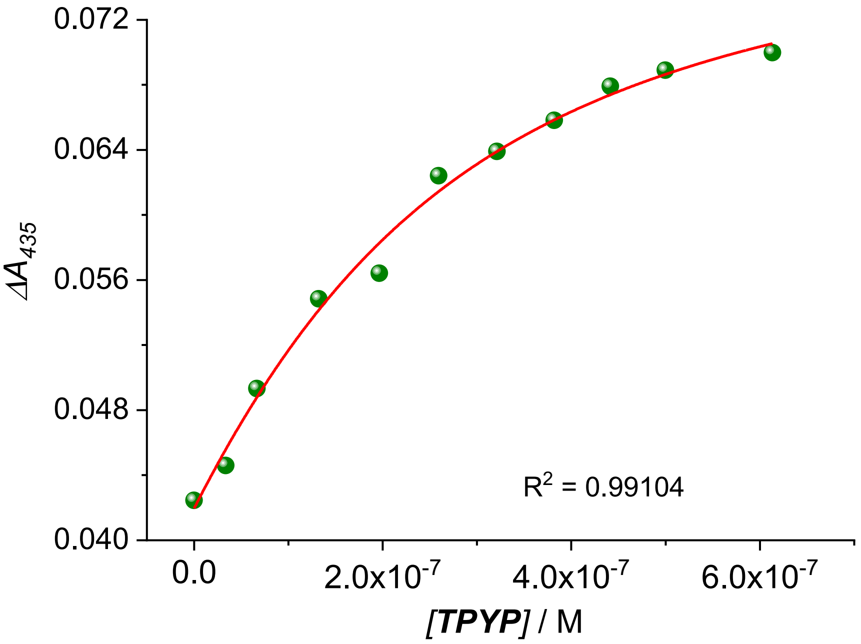

Fig. S18: Changes in ΔA at 435 nm for titration of ***1***$^{2+}$ *with* ***TPyP*** *in toluene. The fitting according to equation [1] is also reported.*

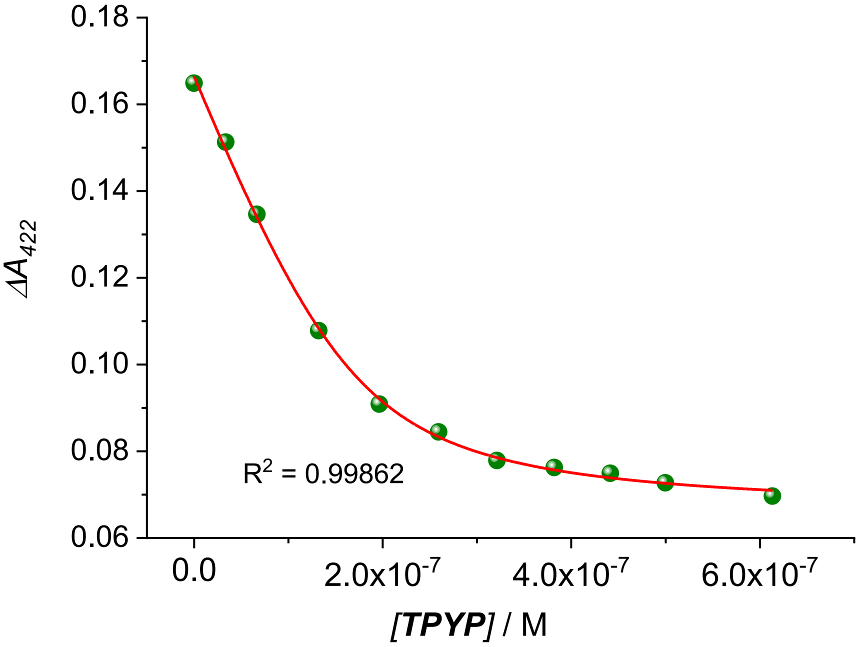

Fig. S19: Changes in ΔA at 422 nm for titration of ***1***$^{2+}$ *with* ***TPyP*** *in toluene. The fitting according to equation [1] is also reported.*

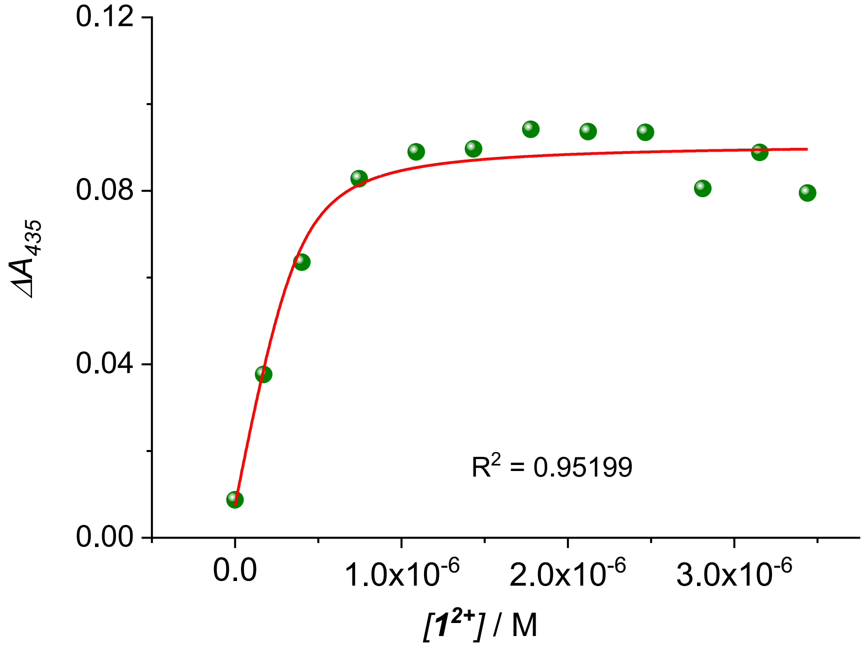

Fig. S20: Changes in ΔA at 435 nm for titration of ***TPyP*** *with* $\mathbf{1}^{2+}$ *in toluene. The fitting according to equation [1] is also reported.*

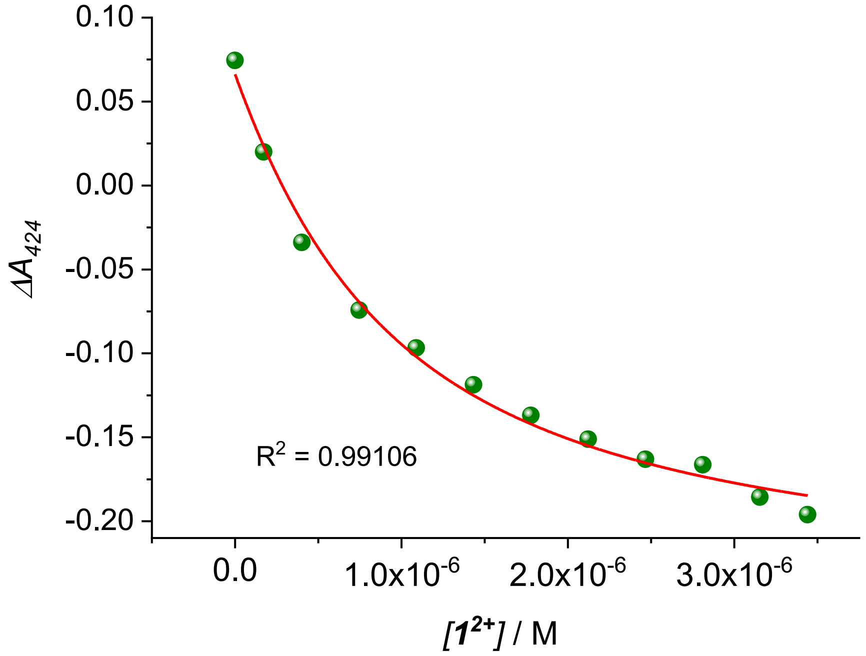

Fig. S21: Changes in ΔA at 424 nm for titration of ***TPyP*** *with* $\mathbf{1}^{2+}$ *in toluene. The fitting according to equation [1] is also reported.*

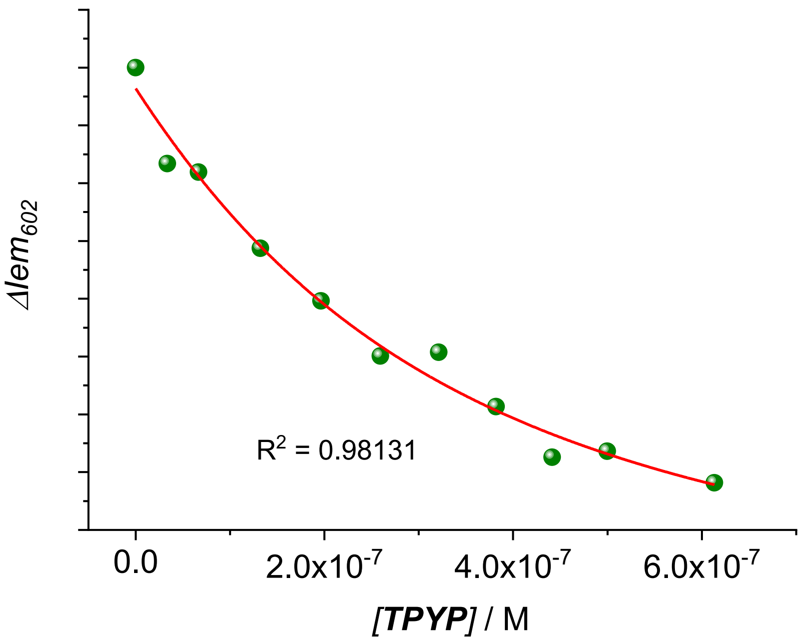

*Fig. S22: Changes in ΔIem at 602 nm for titration of **1**$^{2+}$ with **TPyP** in toluene. The fitting according to equation [1] is also reported.*

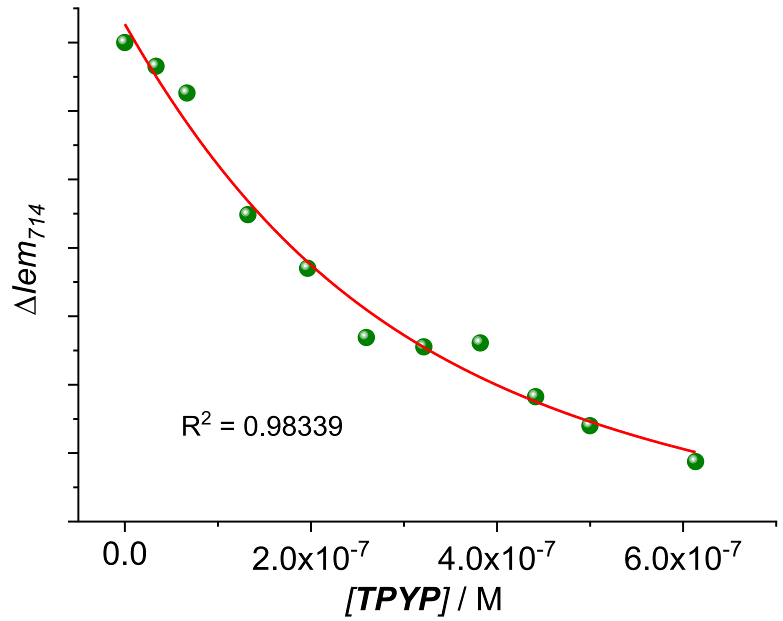

*Fig. S23: Changes in ΔIem at 714 nm for titration of **1**$^{2+}$ with **TPyP** in CH_2Cl_2. The fitting according to equation [1] is also reported.*

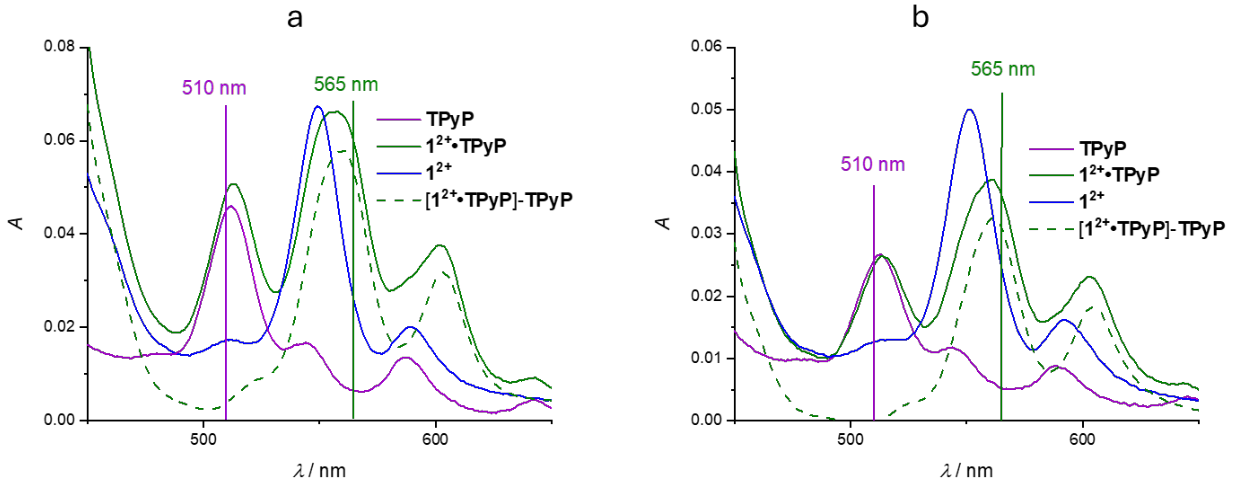

*Fig. S24: Absorption spectra of solutions 10^{-5} M of **1^{2+}** (blue), **TPyP** (purple), and a mixture of **1^{2+}** and **TPyP** (green) in a) CH_2Cl_2 and b) toluene. The spectrum of the complex (green dash) is calculated by subtracting the contribution of **TPyP** from the spectrum of the mixture. The comparison evidences that almost selective excitation of the host (Zn-porphyrin) or prevalent excitation of the guest can be achieved at 565 nm and 510 nm, respectively.*

Electrochemistry of complex 1^{2+}·TPyP in toluene

*Formation of the **1^{2+}·TPyP** complex*

A solution of bis(acridinium-Zn(II)porphyrin)tweezer **1^{2+}** (50 mg, 17.1 µmol, 1 eq.) and 5,10,15,20-Tetra(4-pyridyl)porphyrin (10.57 mg, 17.1 µmol, 1eq.) in $CHCl_3$ (50 mL) was refluxed for 4 h. After evaporation of the solvents, the **1^{2+}·TPyP** complex was used without further purification. ^{1}H NMR (500 MHz, CD_2Cl_2, 298 K) δ (ppm) = 9.12 (d, ^3J = 5.0 Hz, 4H, Py_1), 9.09 (d, ^3J = 5.0 Hz, 4H, Py_2), 9.01 (s, 8H, Py_{3-4}), 8.61 (d, ^3J = 8.0 Hz, 4H, H_b), 8.51 (d, ^3J = 8.5 Hz, 4H, $H_{1/8}$), 8.48 – 8.40 (m, 12H, $H_{4/5-3/6}$), 8.15 (d, ^4J = 2.0 Hz, 8H, H_o), 8.13 (*d*, ^{4}J = 2.0 Hz, 4H, $H_{o'}$), 7.99 – 7.96 (m, 12H, $H_{2/7-Py'}$), 7.83 (t, ^3J = 2.0 Hz, 4H, H_p), 7.83 – 7.82 (m, 4H, H_a), 7.81 (t, ^3J = 2.0 Hz, 4H, $H_{p'}$), 7.11 (br s, 8H, H_β), 6.07 (br s, 8H, H_α), 5.48 (d, ^4J = 1.0 Hz, 1.25 H, $H_{Ol-trans}$), 5.44 (d, ^4J = 1.0 Hz, 0.75 H, H_{Ol-cis}), 5.25 (t, J = 8.0 Hz, 4H, $NC\mathit{H}_2$), 2.33 – 2.29 (m, 4H, CH_2), 2.08 – 2.05 (m, 4H, CH_2), 1.86 – 1.77 (m 4H, CH_2), 1.57 – 1.58 (m, 4H, CH_2), 1.56 – 1.54 (m, 108H, ^{t}Bu), 1.46 (s, 12H, CH_2), −3.75 (s, 2H, NH). ^{13}C{^{1}H} NMR (126 MHz, CD_2Cl_2, 298K) δ (ppm) = 160.8 (s, C_9), 149.2 (s), 149.1 (s), 148.9 (s), 148.1 (s), 147.9 (s), 147.2 (s), 147.1 (s), 144.8 (s), 143.7 (s), 140.9 (s), 139.3 (s), 137.9 (s, $C_{3/6}$), 133.4 (s, C_b), 131.0 (s, C_{Py4}), 130.8 (s, C_{Py3}), 130.7 (s, C_{Py2}), 130.2 (s, C_{Py1}), 129.6 (s, $C_{1/8}$), 129.0 (s, C_a), 128.5 (s, C_o), 128.3 (s, $C_{o'}$), 126.8 (s, C_β), 126.8(s), 126.6 (s, $C_{2/7}$), 125.0 (s), 121.3 (s), 120.1 (s), 119.4 (s, $C_{p-p'}$), 116.7 (s), 116.3 (s, $C_{4/5}$), 115.3 (s), 49.8 (s, NCH_2), 33.5 (s), 33.5 (s), 33.4 (s), 30.0 (s, ^{t}Bu), 30.0 (s, ^{t}Bu), 28.0 (s), 27.3 (s).

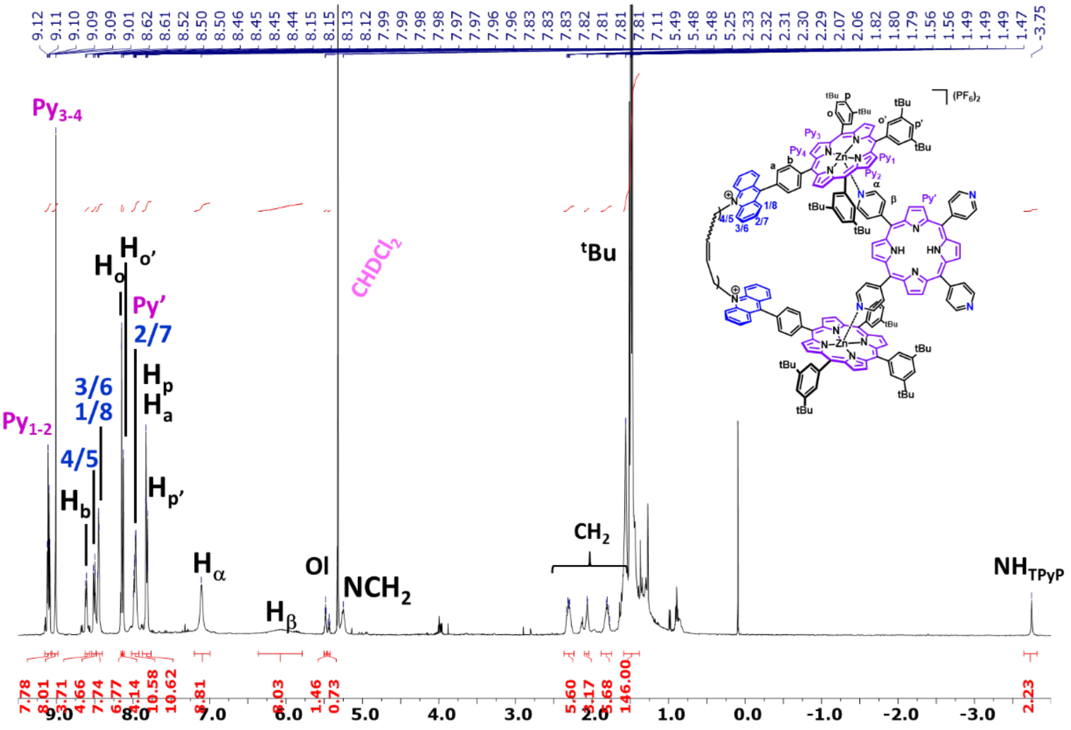

Fig. S25: ^{1}H NMR (500 MHz, CD_2Cl_2, 298 K) spectrum of $\mathbf{1^{2+}{\cdot}TPyP}$.

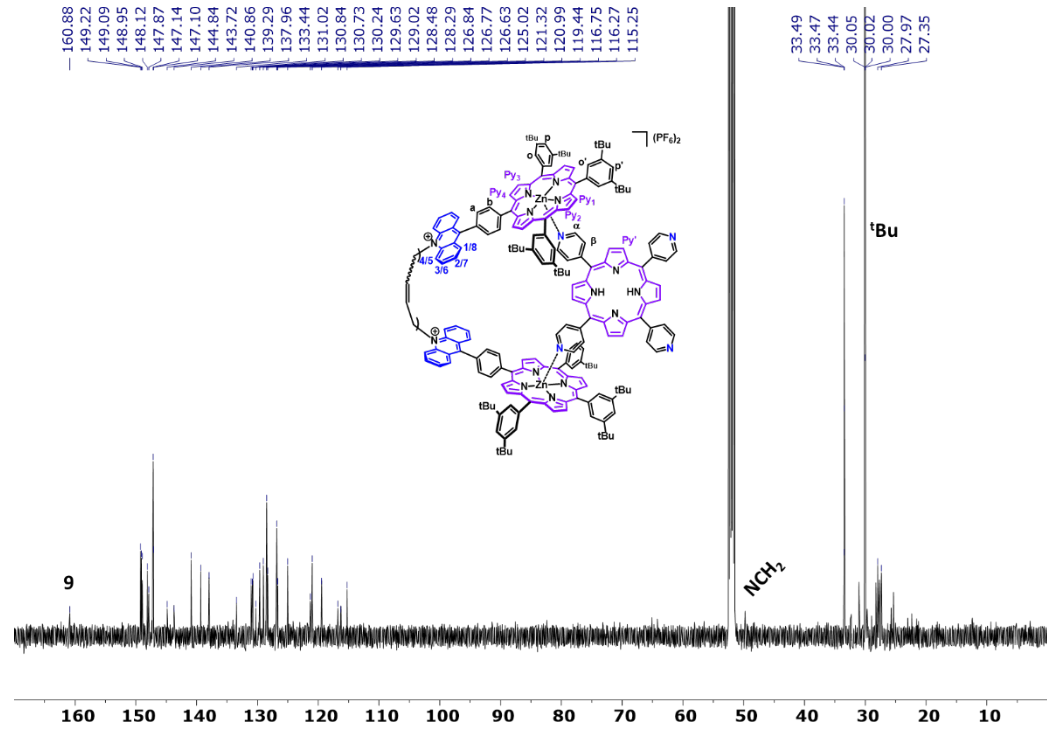

Fig. S26: $^{13}C\{^{1}H\}$ NMR (500 MHz, CD_2Cl_2, 298 K) spectrum of $\mathbf{1^{2+}{\cdot}TPyP}$.

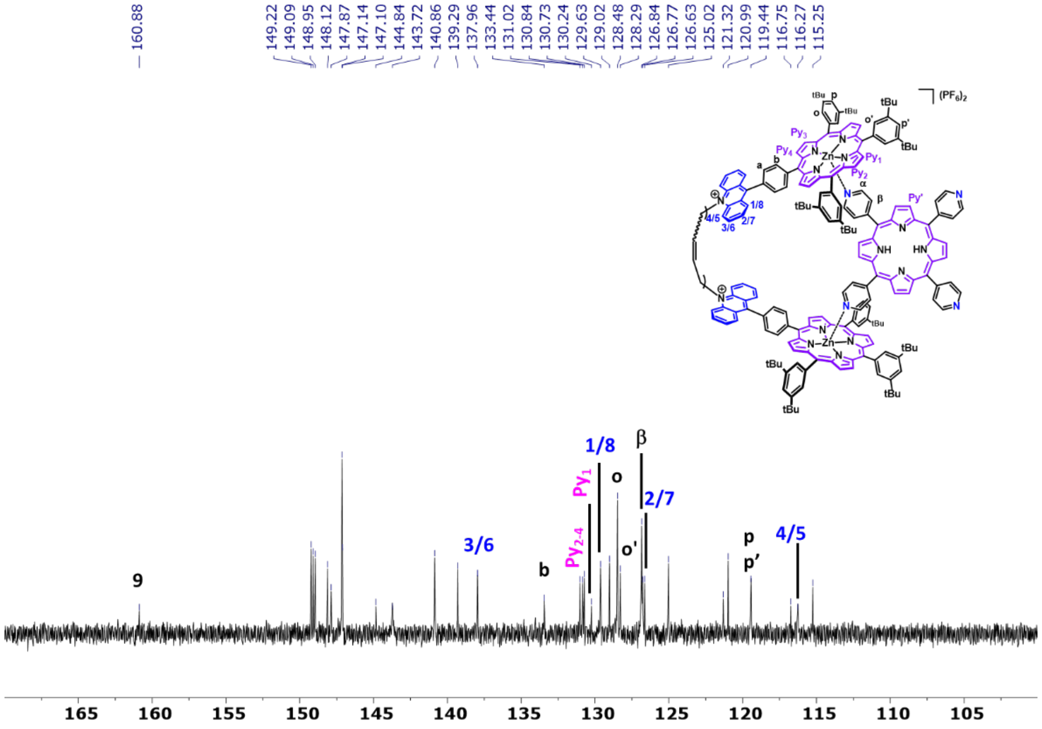

*Fig. S27: $^{13}C\{^{1}H\}$ NMR (500 MHz, CD_2Cl_2, 298 K) spectrum of **1^{2+}·TPyP** (zoom aromatic region).*

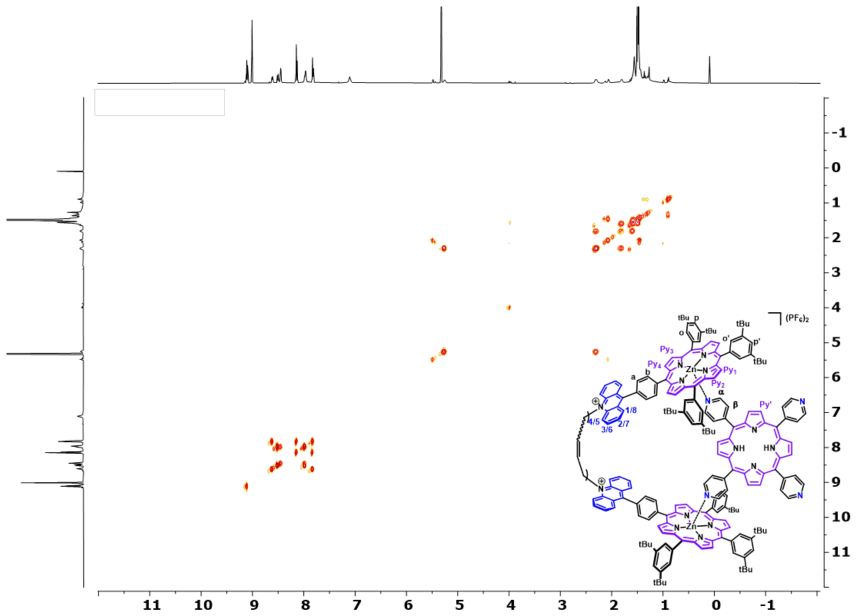

*Fig. S28: ^{1}H–^{1}H gCOSY 2D-spectrum (500 MHz, CD_2Cl_2, 298 K) of **1^{2+}·TPyP**.*

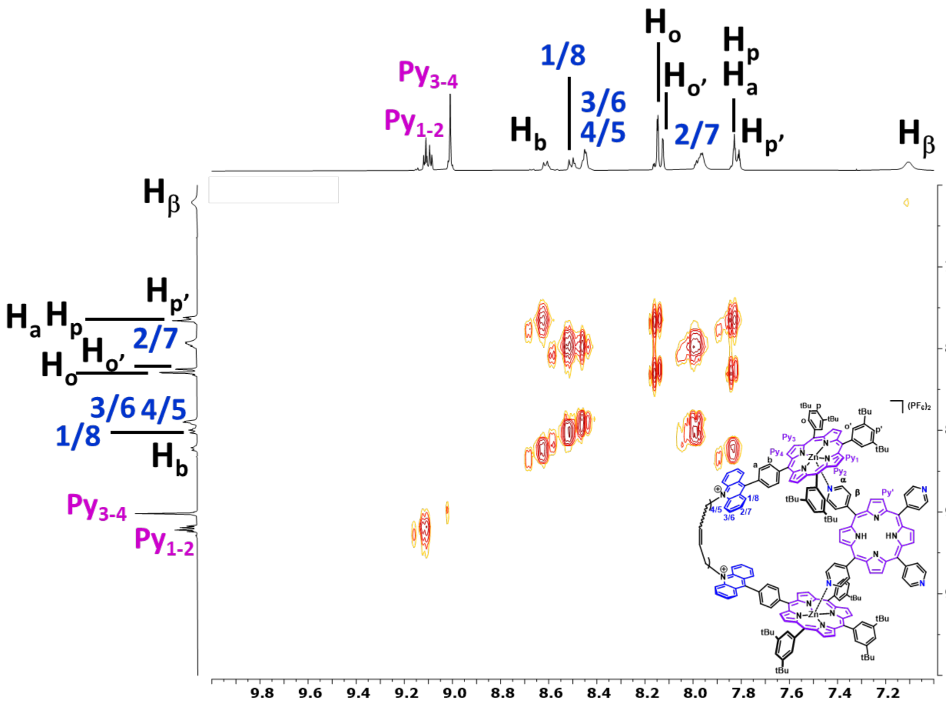

Fig. S29: ^{1}H*–*^{1}H *gCOSY 2D-spectrum (500 MHz,* CD_2Cl_2*, 298 K) of* $\mathbf{1^{2+}\cdot TPyP}$ *(zoom aromatic region).*

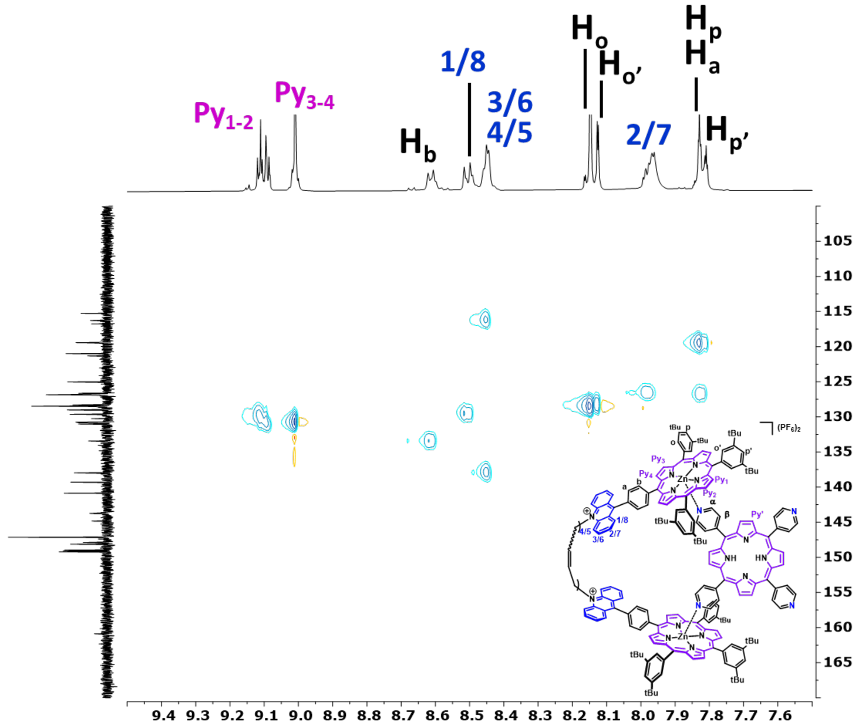

Fig. S30: ^{1}H*–*^{13}C *gHSQC 2D-spectrum (500 MHz,* CD_2Cl_2*, 298 K) of* $\mathbf{1^{2+}\cdot TPyP}$ *(zoom aromatic region).*

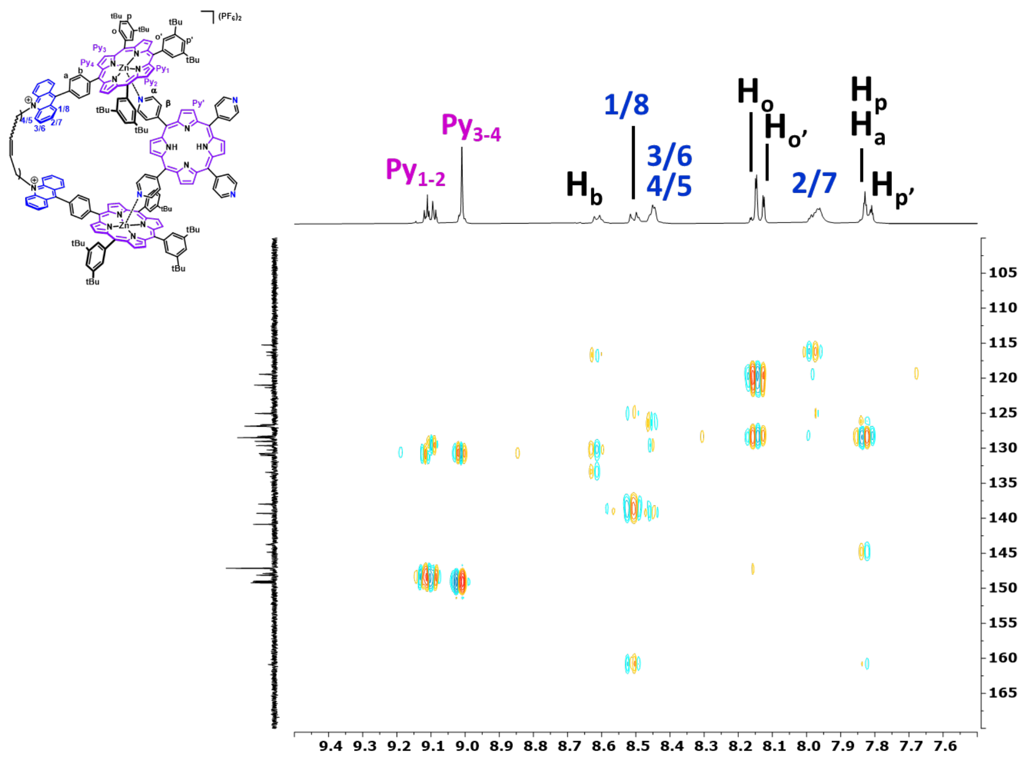

Fig. S31: ^{1}H–^{13}C gHMBC 2D-spectrum (500 MHz, CD_2Cl_2, 298 K) of $\mathbf{1^{2+}{\cdot}TPyP}$ (zoom aromatic region).

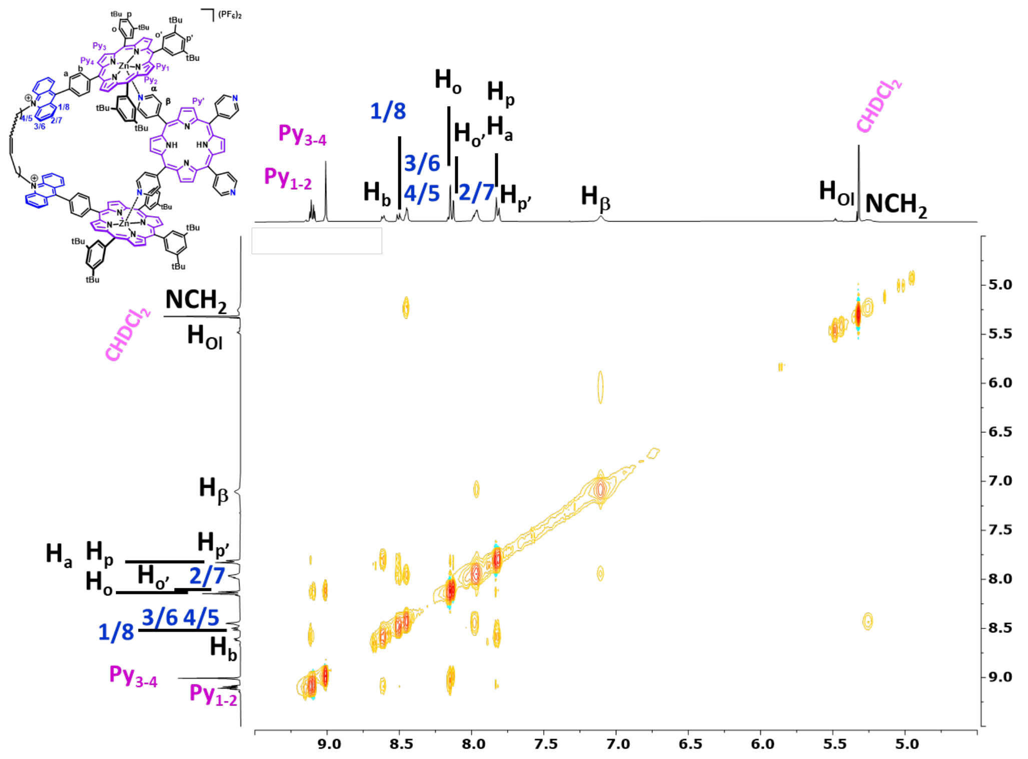

Fig. S32: ^{1}H–^{1}H NOESY 2D-spectrum (500 MHz, CD_2Cl_2, 298 K) of $\mathbf{1^{2+}{\cdot}TPyP}$ (zoom aromatic region).

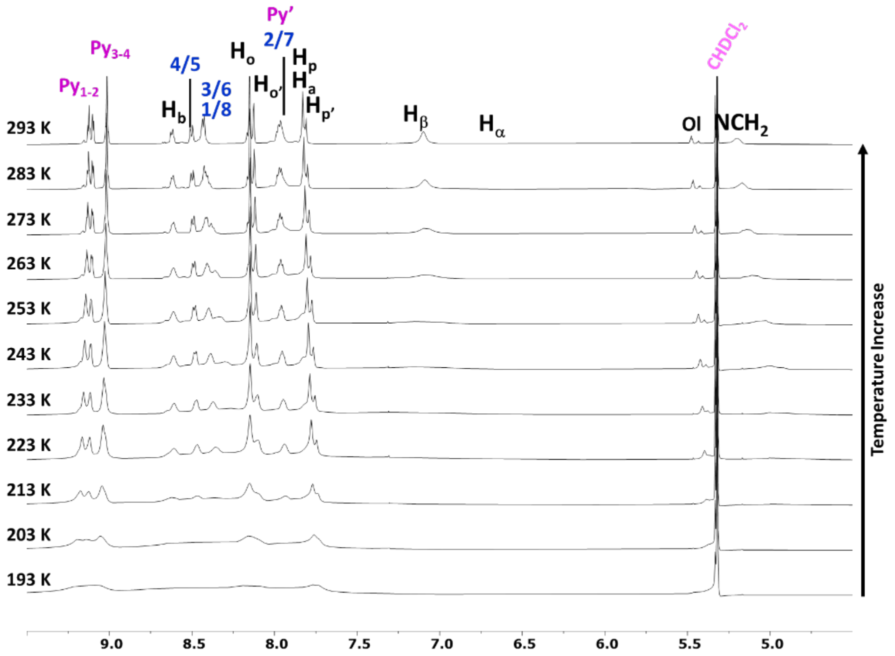

*Fig. S33: ^{1}H NMR (500 MHz, CD_2Cl_2) spectrum of **1^{2+}·TPyP** at variable temperatures (zoom between 9.5 and 4.5 ppm).*

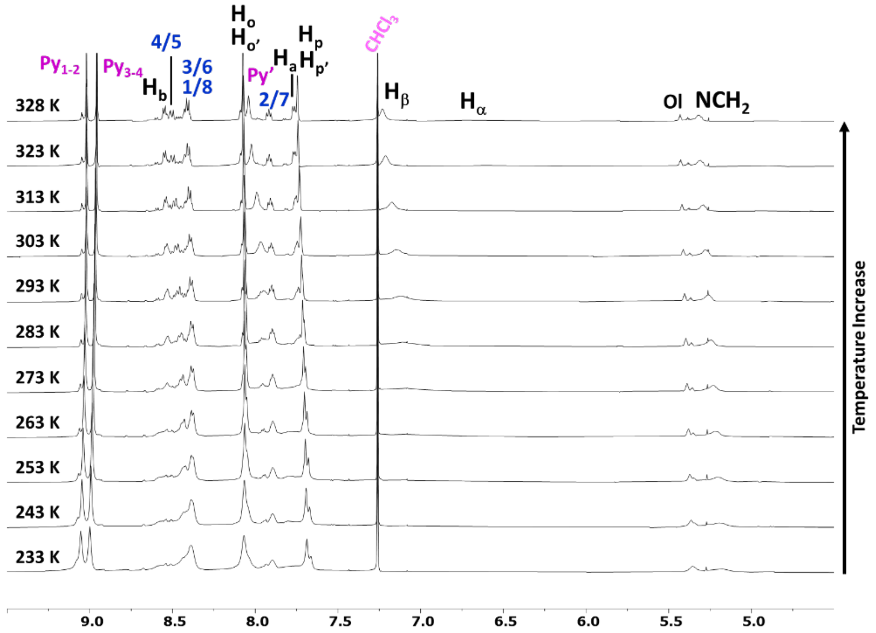

*Fig. S34: ^{1}H NMR (500 MHz, $CDCl_3$) spectrum of **1^{2+}·TPyP** at variable temperatures (zoom between 9.5 and 4.5 ppm).*

Table S3: Chemical shift values (in ppm) of the protons of the receptor ***1***$^{2+}$ *and the complex* ***1***$^{2+}$***·TPyP*** *and the chemical shift difference for each proton.*

	**1**$^{2+}$	**1**$^{2+}$**·TPyP**	$\Delta\delta$ (ppm)
Py_1	9.15	9.12	–0.03
Py_2	9.12	9.09	–0.03
Py_3	9.03	9.01	–0.02
Py_4	9.03	9.01	–0.02
$H_{1/8}$	8.59	8.51	–0.08
$H_{2/7}$	8.09	7.97	–0.12
$H_{3/6}$	8.54	8.45	–0.09
$H_{4/5}$	8.61	8.45	–0.16
H_o	8.13	8.15	+0.02
H_p	7.87	7.83	–0.04
$H_{o'}$	8.09	8.13	+0.04
$H_{p'}$	7.87	7.81	–0.06
H_a	7.92	7.82	–0.10
H_b	8.63	8.61	–0.02

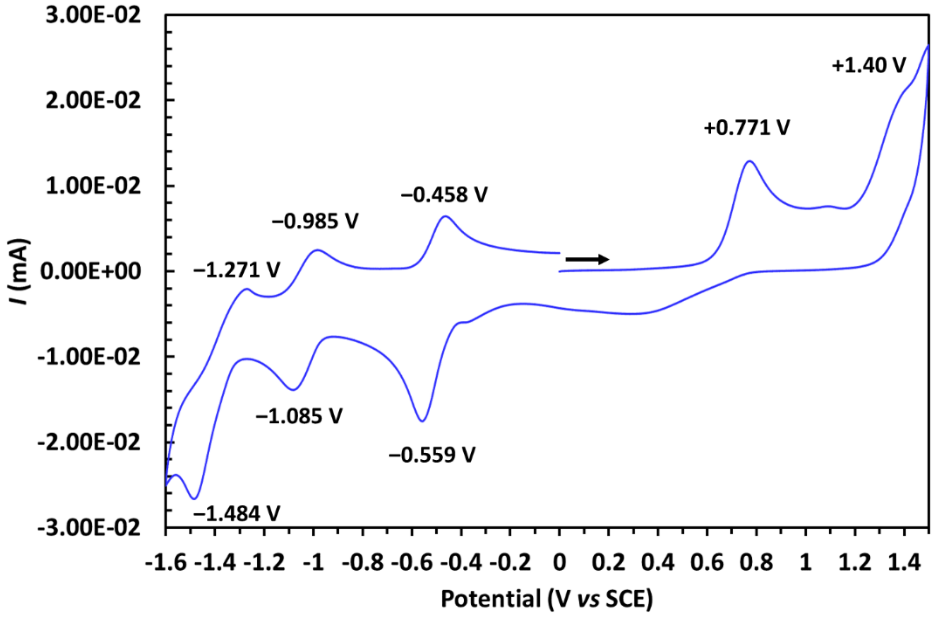

Fig. S35: Cyclic voltammogram ($C_2H_4Cl_2$, WE: Pt, CE: Pt, Ref: Hg/Hg_2Cl_2/saturated KCl, 0.1 mol L^{-1} of $TBAPF_6$) of a solution of ***1***$^{2+}$***·TPyP*** *($c = 1 \times 10^{-3}$ mol L^{-1}) at a scan rate of 100 mV s^{-1}. Potentials were measured by cyclic voltammetry and are referenced versus Hg/Hg_2Cl_2/saturated KCl.*

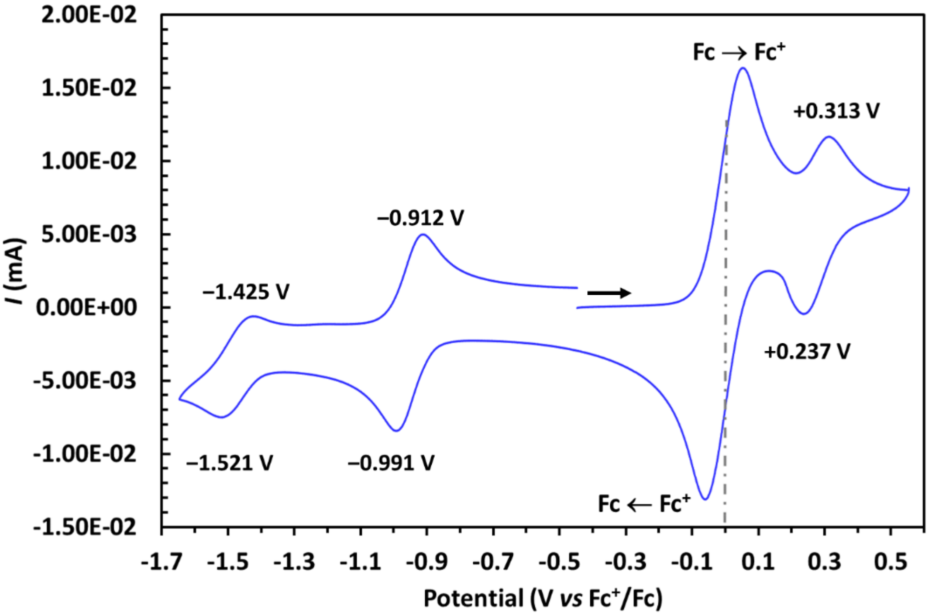

Fig. S36: Cyclic voltammogram ($C_2H_4Cl_2$, WE: Pt, CE: Pt, Ref: Hg/Hg_2Cl_2/saturated KCl, 0.1 mol L^{-1} of $TBAPF_6$) of a solution of ***1^{2+}•TPyP*** *(c = 5 × 10^{-4} mol L^{-1}) in the presence of ferrocene (Fc, c = 5 × 10^{-4} mol L^{-1}) at a scan rate of 100 mV s^{-1}. Potentials were measured by cyclic voltammetry and are referenced versus Fc^{+}/Fc couple.*

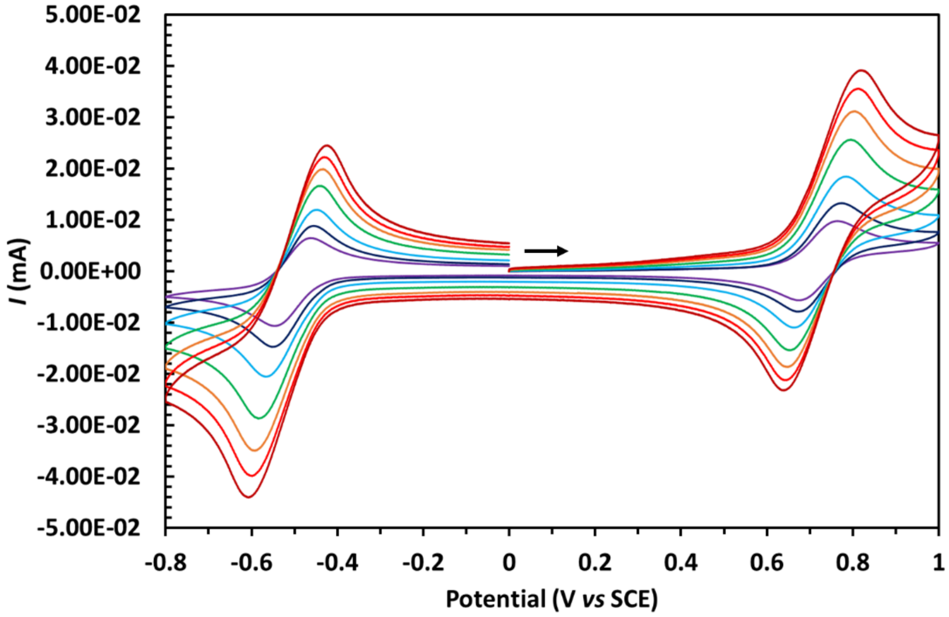

Fig. S37: Cyclic voltammogram ($C_2H_4Cl_2$, WE: Pt, CE: Pt, Ref: Hg/Hg_2Cl_2/saturated KCl, 0.1 mol L^{-1} of $TBAPF_6$) of a solution of ***1^{2+}•TPyP*** *(c = 1 × 10^{-3} mol L^{-1}) at a scan rate of 50 (purple), 100 (dark blue), 200 (light blue), 400 (green), 600 (orange), 800 (light red) and 1000 mV s^{-1} (dark red). Potentials are referenced versus Hg/Hg_2Cl_2/saturated KCl.*

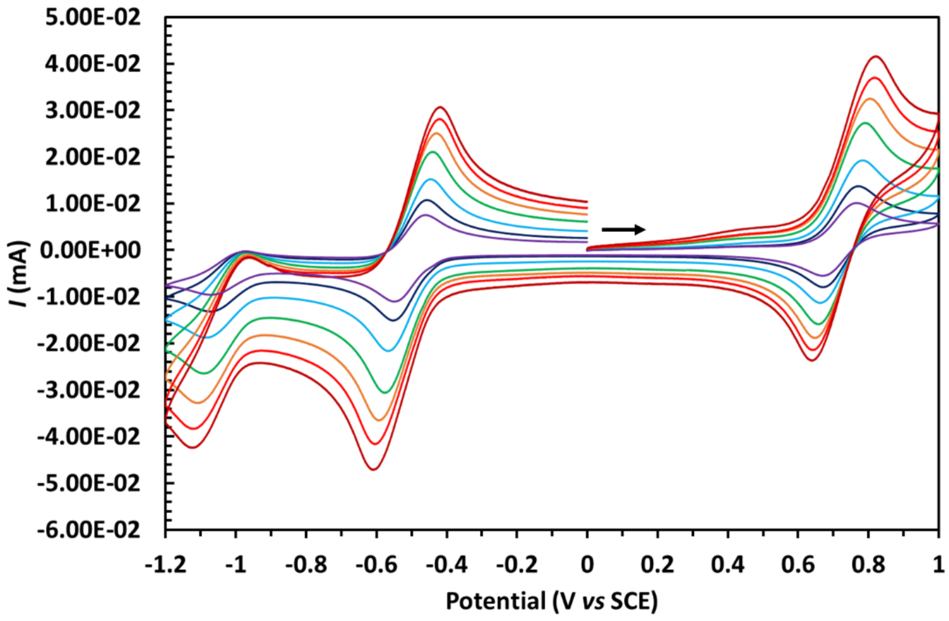

Fig. S38: Cyclic voltammogram ($C_2H_4Cl_2$, WE: Pt, CE: Pt, Ref: Hg/Hg_2Cl_2/saturated KCl, 0.1 mol L^{-1} of $TBAPF_6$) of a solution of ***1^{2+}·TPyP*** *(c = 1 × 10^{-3} mol L^{-1}) at a scan rate of 50 (purple), 100 (dark blue), 200 (light blue), 400 (green), 600 (orange), 800 (light red) and 1000 mV s^{-1} (dark red). Potentials are referenced versus Hg/Hg_2Cl_2/saturated KCl.*

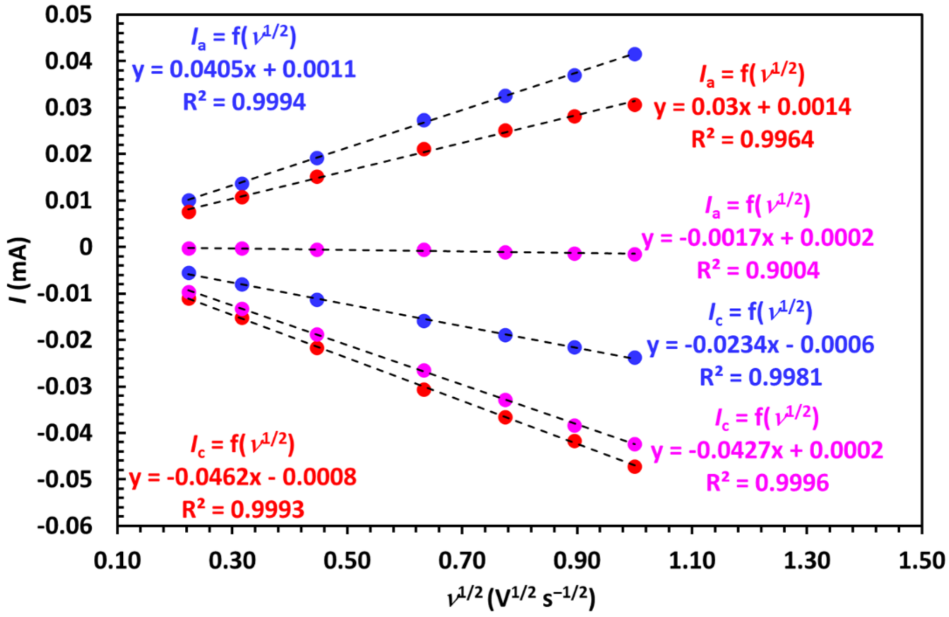

Fig. S39: Current as a function of the square root of the scan rate of the cathodic (I_c) and anodic current (I_a) of the first oxidation (blue), first reduction (red) and second reduction (pink) process of ***1^{2+}·TPyP*** *in $C_2H_4Cl_2$.*

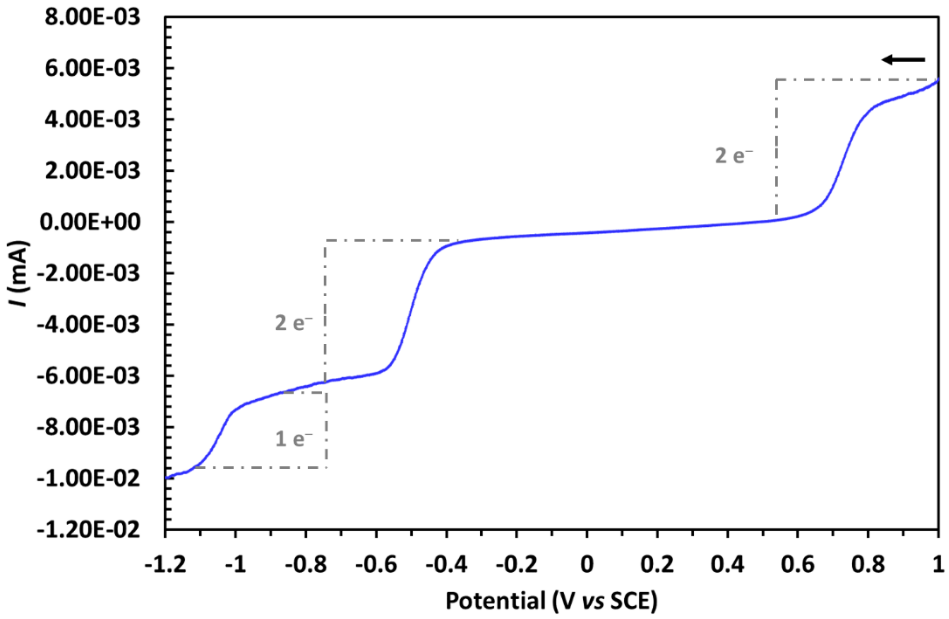

*Fig. S40: RDE experiment ($C_2H_4Cl_2$, WE: Pt, CE: Pt, Ref: Hg/Hg_2Cl_2/saturated KCl, 0.1 mol L^{-1} of $TBAPF_6$) of a solution of **1^{2+}·TPyP** ($c = 5 \times 10^{-4}$ mol L^{-1}) recorded at scan rate of 100 mV s^{-1} and rotation speed of 1000 rpm. Potentials are referenced versus Hg/Hg_2Cl_2/saturated KCl.*

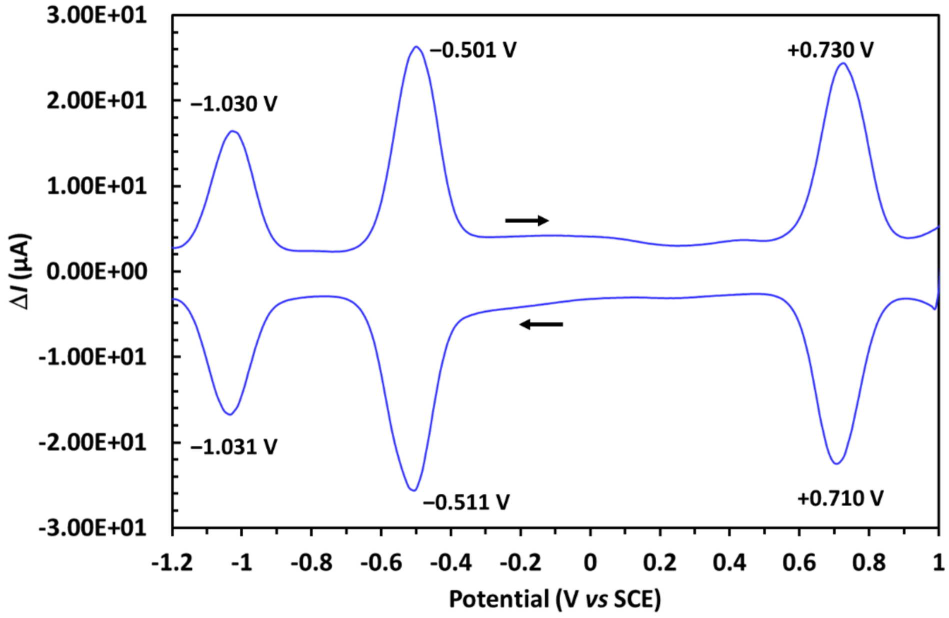

*Fig. S41: Square wave voltammogram ($C_2H_4Cl_2$, WE: Pt, CE: Pt, Ref: Hg/Hg_2Cl_2/saturated KCl, 0.1 mol L^{-1} of $TBAPF_6$) of a solution of **1^{2+}·TPyP** ($c = 5 \times 10^{-4}$ mol L^{-1}) in the presence of ferrocene (1 eq.). Potentials are referenced versus Fc^+/Fc.*

*Table S4: Cathodic (E_c), anodic (E_a), half potentials ($E_{1/2}$) and peak-to-peak difference (DE) of **1^{2+}·TPyP** in $C_2H_4Cl_2$.*

		E_c (V *vs* Fc^+/Fc)	E_a (V *vs* Fc^+/Fc)	$E_{1/2}$ (V *vs* Fc^+/Fc)	ΔE (mV)
**1^{2+}·TPyP**	**2nd Oxidation**	+0.942	-	-	-
	**1st Oxidation**	+0.313	+0.237	+0.275	76
	**1st Reduction**	–0.991	–0.912	–0.952	79
	**2nd Reduction**	–1.521	–1.425	–1.473	99
	**3rd Reduction**	–1.942	-	-	-
**1^{2+}**	**1st Oxidation**	+0.325[a]	+0.249[a]	+0.287[a]	87[a]
	**1st Reduction**	−1.032[a]	–0.953[a]	–0.993[a]	67[a]

[a] *Taken from Ref [6].*

Modelling

*Reduction of **TPyP** and analysis of the UV-VIS absorption properties*

The reduced form of **TPyP** and the absorption spectral features of both neutral and reduced **TPyP** were investigated by DFT and TD-DFT calculations.

The one-electron reduction of neutral **TPyP** does not significantly alter its structure, as shown in Fig. S42 with a root mean square displacement (RMSD) close to zero (0.060). The frontier molecular orbitals for both neutral and reduced **TPyP** are illustrated in Fig. S43. In neutral **TPyP**, the HOMO is located in the N p-orbital and in the π-orbital ring, while the LUMO is in the π* orbital and in the non-protonated N p-orbital. The reduced form shows the SOMO in the π-orbital and in the protonated N p-orbital, while the LUMO displays the same orbital observed previously. In reduced **TPyP** the spin density distribution (Fig. S43c) is primarily localized over the central conjugated core of the porphyrin ring and partially extends onto the peripheral substituents, suggesting delocalization of the unpaired electron(s) across the π-system. The calculated E_{red} potentials in dichloromethane and toluene are 1.03 V and 1.42 V, respectively, in line with the results of the electrochemical characterization (see main text) and literature data.[26]

In Table S5 are reported the absorption properties of neutral **TPyP**, including calculated absorption wavelengths, oscillator strengths (*f*), transition molecular orbitals and their coefficients in CH_2Cl_2. The calculated absorption spectrum, shown in Fig. S44, differs from the experimental one by around 30 nm, consistent with results from global hybrid functionals.[27] The Q bands, with a low absorption coefficient, appears between 550 and 600 nm, while the Soret band, with a higher absorption coefficient, is observed in the range 400–410 nm. These transitions are in line with Gouterman's Four Orbital Theory,[28] involving the HOMO, HOMO-1, LUMO, and LUMO-1 orbitals (Table S5). As shown in Table S5, during the $S_0 \rightarrow S_1$ transition, the HOMO-1 is localized in the ring's π orbital, while the HOMO extends to the N atoms' p-orbital. The LUMO is in the ring's π* orbital and the non-protonated N atoms' p-orbital, while LUMO+1 is localized in the π* orbital and protonated N atoms' p-orbital. In the $S_0 \rightarrow S_2$ transition, the electron density shifts between LUMO and LUMO+1, while in $S_0 \rightarrow S_3$ and $S_0 \rightarrow S_4$, the change occurs between HOMO-1 and HOMO.

The simulated absorption spectrum of the reduced form shows new bands at 1016 nm and 2180 nm, in the infrared region. The Q bands are red shifted (746-666 nm), and the Soret band shows multiple transitions between 455 and 536 nm (Fig. S45). The ground state of reduced **TPyP** is a doublet state

and high spin contaminations are observed, complicating the band assignment. Fig. S46 shows the electron density difference map between exited state (S_1 or S_2) and ground state (S_0). For the first excited state (S_1–S_0), the electron distribution is delocalized extensively over the porphyrin macrocycle, indicative of a $\pi\rightarrow\pi^*$ transition typical of lower-energy Q-band excitations. Conversely, the second excited state (S_2–S_0) shows a more localized electron redistribution mainly centered on the porphyrin ring core, suggesting excitation into higher-lying molecular orbitals and reflecting a distinct electronic nature compared to S_1.

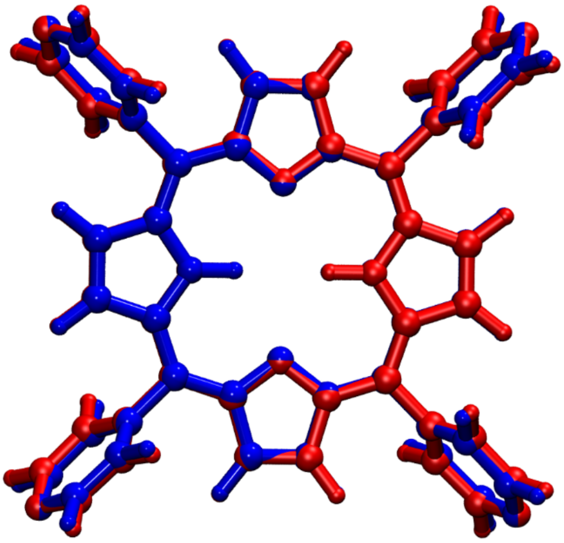

*Fig. S42: Overlap of optimized structures for **TPyP** (blue) and its reduced form (red). The only significant change observed is in the dihedral angle of the pyridine units, which shifts from 73° in neutral **TPyP** to around 67° in the reduced form.*

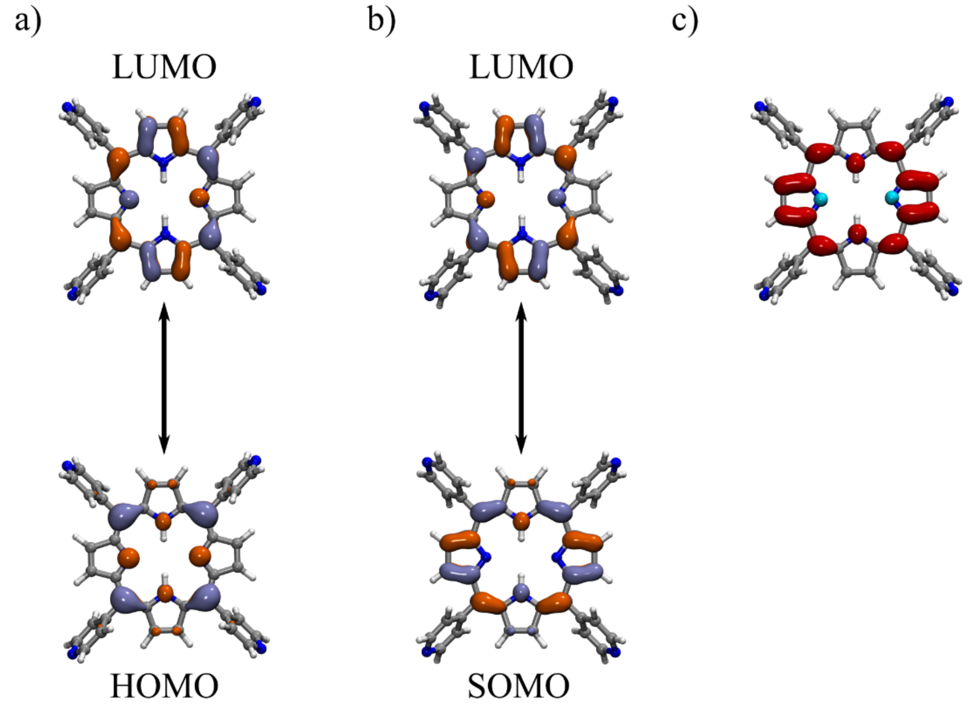

*Fig. S43: a) HOMO and LUMO orbitals of **TPyP**; b) SOMO and LUMO orbitals of reduced **TPyP** in gas phase (isovalues 0.04 a.u.); c) spin density distribution of reduced **TPyP**. The red isosurfaces represent regions of positive spin density, while the cyan isosurfaces indicate regions of negative spin density (isovalues 0.002 a.u).*

*Table S5: Absorption peaks, calculated wavelengths (λ), f values (oscillator strength of each transition) and natural transition orbitals (NTOs) for **TPyP**. Isovalue 0.04 $e^{-1/2}$ $bohr^{-3/2}$.*

Absorption peak		λ (nm)	f	MO/character
Q	$S_0 \rightarrow S_1$	604	0.005	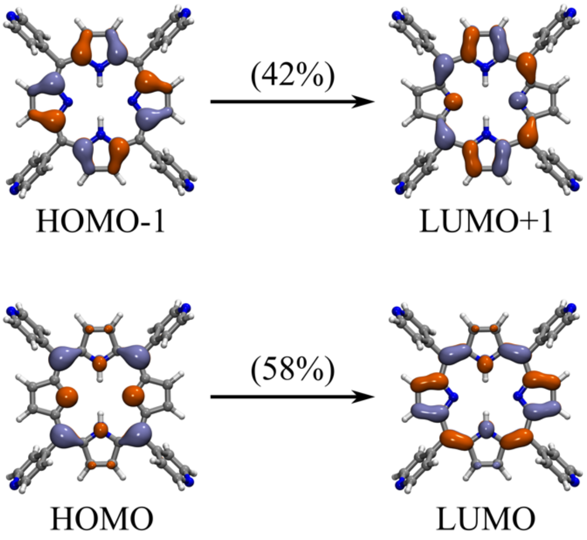

<table>
<tr><td rowspan="6">Soret</td><td rowspan="2">$S_0 \rightarrow S_2$</td><td rowspan="2">554</td><td rowspan="2">0.09</td><td>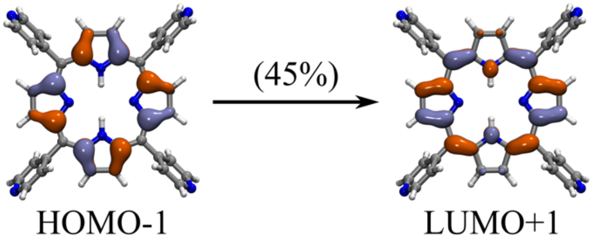
</td></tr>
<tr><td>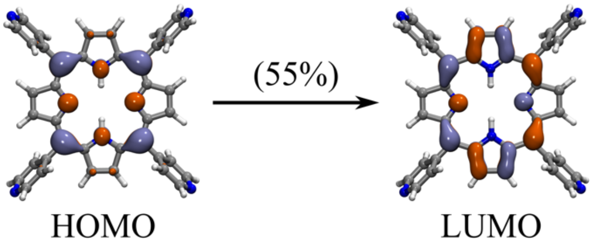
</td></tr>
<tr><td rowspan="2">$S_0 \rightarrow S_3$</td><td rowspan="2">411</td><td rowspan="2">2.075</td><td>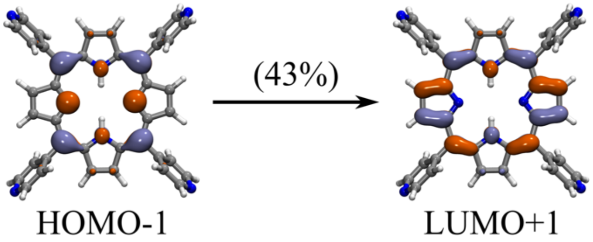
</td></tr>
<tr><td>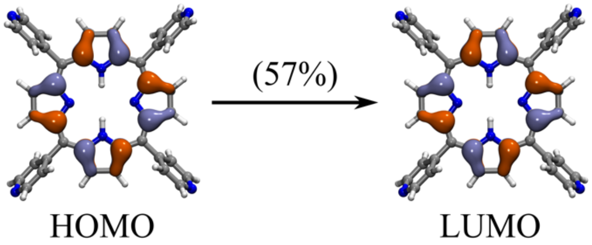
</td></tr>
<tr><td rowspan="2">$S_0 \rightarrow S_4$</td><td rowspan="2">407</td><td rowspan="2">2.212</td><td>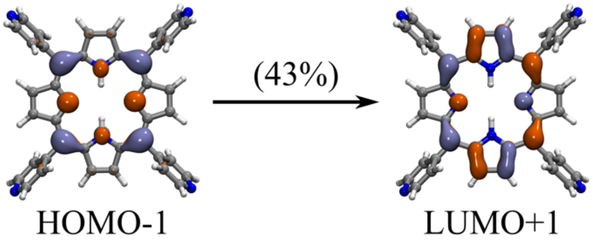
</td></tr>
<tr><td>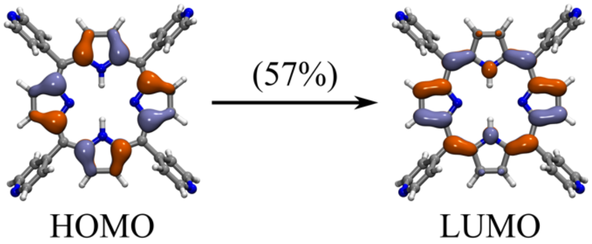
</td></tr>
</table>

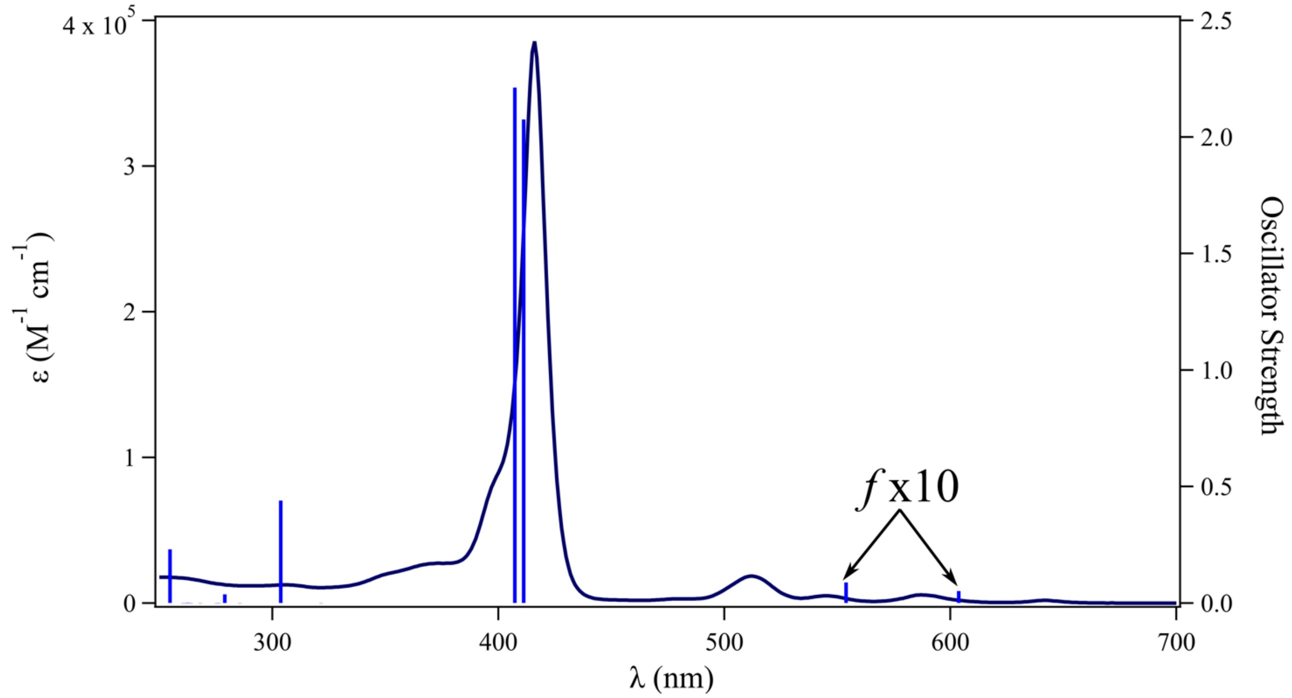

*Fig. S44: Overlap between the experimental absorption spectrum of **TPyP** (dark curve) and the calculated vertical excitation transitions (blue lines) at WB97X-D/def-TZVP /CH_2Cl_2. The Q bands f values were multiplied by 10.*

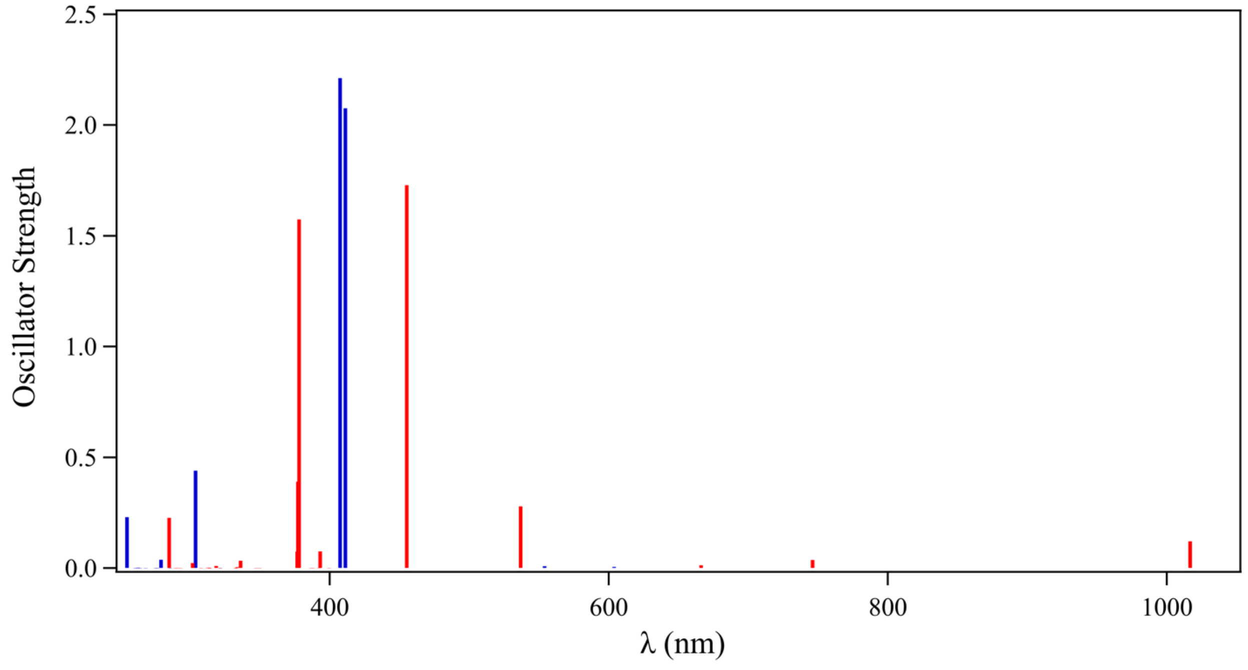

*Fig. S45: Overlap between the calculated vertical excitation transitions of **TPyP** (blue bars) and its reduced form (red bars) calculated at WB97X-D/def-TZVP /CH_2Cl_2.The first vertical excitation transition at 2180 nm for reduced **TPyP** shows f = 0.000 and it's not reported in the plot.*

a) b)

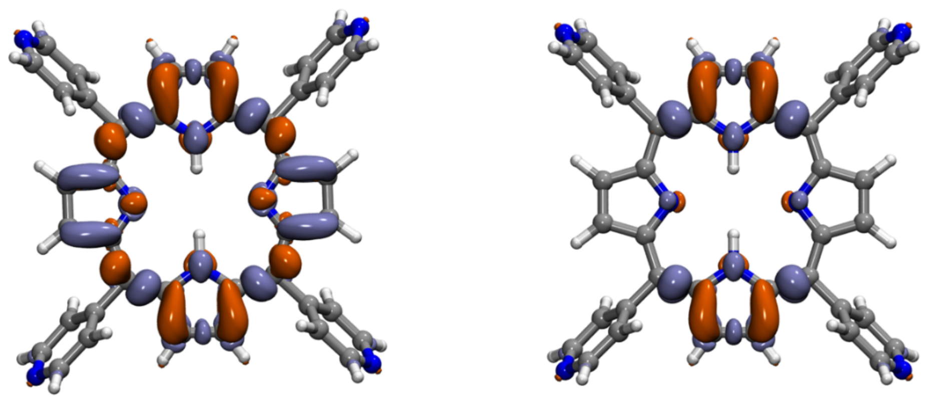

*Fig. S46: electron density difference map between exited state (S_1 a) or S_2 b)) and ground state (S0) computed for reduced **TPyP** (isovalues 0.002 a.u). Orange and ice-blue regions represent gain and loss of electron density, respectively, upon excitation.*

Dynamic properties

Using the GNF2-xTB method, the dynamic properties of compound $\mathbf{1}^{2+}$ were evaluated. After the equilibration phase, $\mathbf{1}^{2+}$ adopts an open conformation (structure 1 in Fig. S47a). However, the root mean square deviation (RMSD) plot *vs.* time (Fig. S47a, yellow curve) shows significant fluctuations during the first 0.5 ns of the simulation, corresponding to the progressive folding of the central alkyl chain (structures 2 and 3 in Fig. S47a), leading to the formation of a π-π interaction between one ZnTPP moiety and Acr (Fig. S47b). The RMSD plot was further split into individual contributions, revealing that the largest conformational modifications are related to the ZnTPP involved in the folding process (light blue curve) and to the alkyl chain (red curve). Once this movement is concluded, bringing the system to a so-called "closed" state (structures 4-6 in Figure S26a), the RMSD plot shows minimal variations, remaining below 5 Å, indicating that this conformation is stable until the end of the simulation. A more effective way to analyze this structure is by using the combined distribution function (CDF), which can provide more detailed information with respect to the commonly used radial (g(r)) or angular distribution functions alone. CDFs offer insights into distance–angle correlations, which can be crucial for isolating the spatial contributions of different parts of a radial distribution function.[29] To this end, a CDF combining the Zn–N_{Acr} g(r) and the N–Zn–N_{Acr} angular distributions was calculated (Fig. S47c). The high-probability region (red zone) is located at a distance close to 3.5 Å and an angle of about 80°, consistent with the presence of π-π interactions between one ZnTPP and Acr. This behavior is further confirmed by the distance *vs.* time plots, where the considered distance is between Zn atoms and N or C atoms of the Acr moiety (Zn-N_{Acr}, Zn-C_{Acr}, Fig. S47d): after an initial decrease from approximately 25-30 Å, the distances stabilize at an average value of 4.5 Å and 4.1 Å, respectively.

Comparison of the SASA plot (Fig. S47e) for $\mathbf{1}^{2+}$ in the closed conformation (yellow line after 0.3 ns) with that of *cis*-$\mathbf{1}^{2+}$•**TPyP** (blue line) and *trans*-$\mathbf{1}^{2+}$•**TPyP** (red line) reveals an increase in the accessible surface area, slightly higher for *trans*-$\mathbf{1}^{2+}$•**TPyP**, due to the partial opening of $\mathbf{1}^{2+}$ caused by complexation. On the other hand, similar SASA values were observed when comparing the plots for the open form of $\mathbf{1}^{2+}$ (Figure S26e yellow line, 0.0–0.3 ns) with the *cis*- and *trans*-$\mathbf{1}^{2+}$•**TPyP** models. Conversely, a sharp decrease in SASA for **TPyP** (Fig. S48b) is observed after complexation.

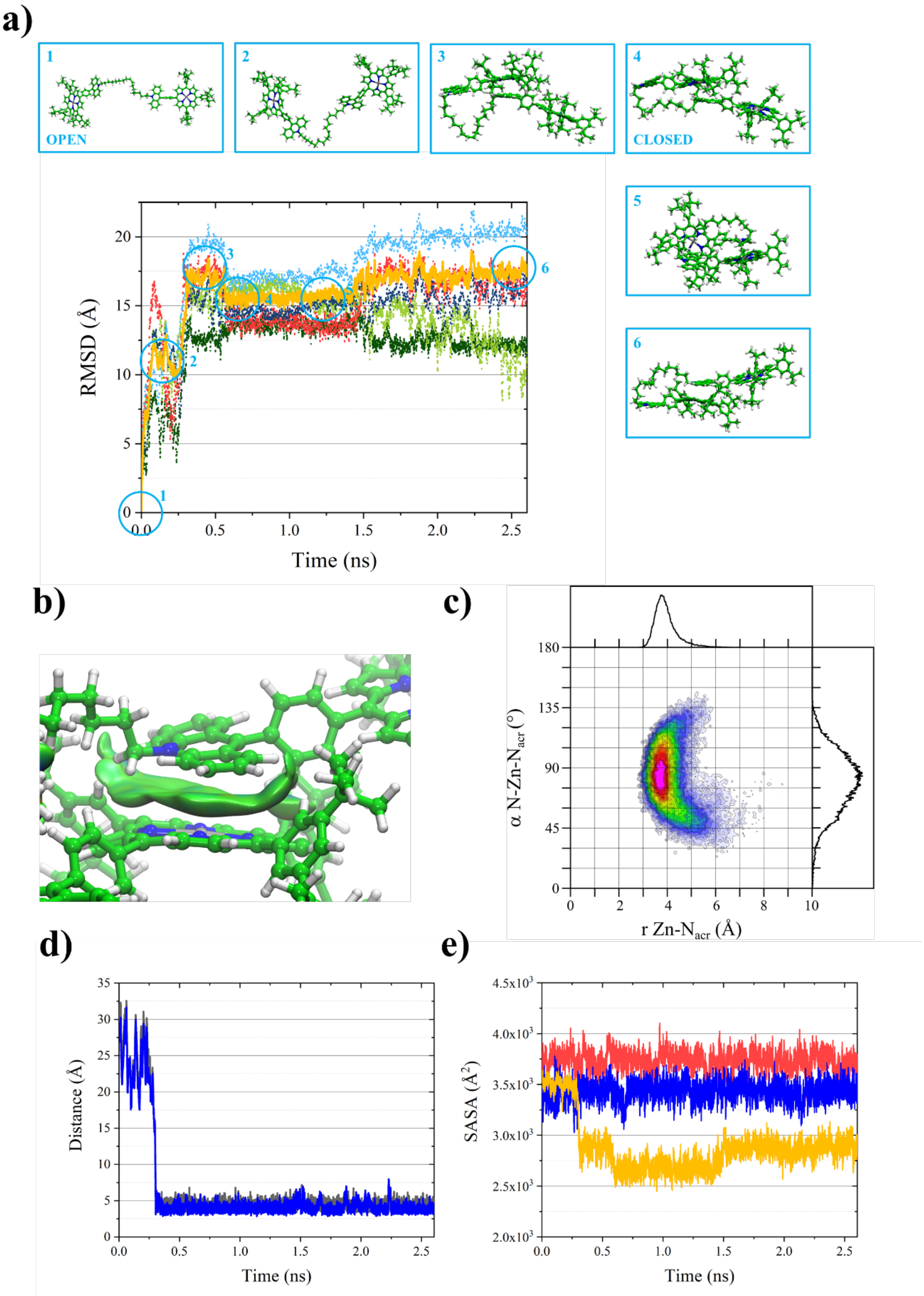

*Fig. S47: Root mean square displacement (RMSD) vs. time for: $\mathbf{1}^{2+}$ (yellow curve), Acr-1 (dark green), Acr-2 (light green), ZnTPP-1 (dark blue), ZnTPP-2 (light blue), alkyl chain (red) with most representative structures (the two Acr and ZnTPP moieties have been considered as independent units). b) Closed structures with the 3D IGM surfaces are mapped using the product of the electron density and the second eigenvalue of the electron-density Hessian matrix. Green colored regions indicate non-covalent interactions as π–π interaction. c) Combined distribution function (CDF) between radial distributions function of Zn–N_{Acr} distances and angles distributions function of N-Zn-N_{Acr} angles are calculated from the MD simulation. d) Distance vs. time between: Zn atom of ZnTPP and N atom of Acr (Zn-N_{Acr}, blue line); Zn atom of ZnTPP and C atom of Acr (Zn-C_{Acr}, grey line). e) Solvent accessible surface area (SASA) for: $\mathbf{1}^{2+}$ (yellow line), cis-$\mathbf{1}^{2+}$•**TPyP** (blue line) and trans-$\mathbf{1}^{2+}$•**TPyP** (red line).*

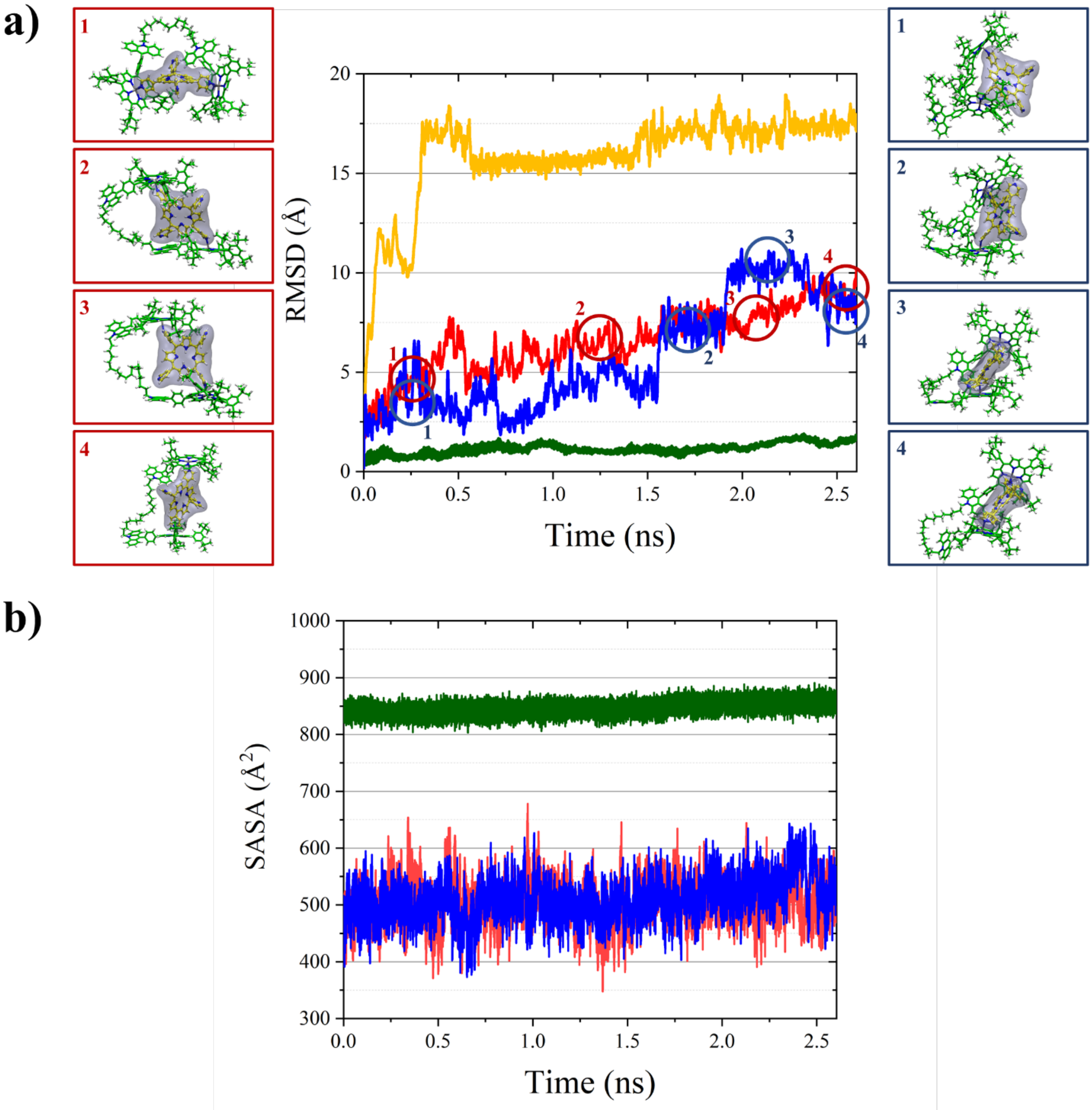

Fig. S48: a) Root mean square displacement (RMSD) vs. time for: ***1***$^{2+}$ *(yellow line),* ***TPyP*** *(dark green line), cis-****1***$^{2+}$***•TPyP*** *(blue line) and trans-****1***$^{2+}$***•TPyP*** *(red line). b) Solvent accessible surface area (SASA) for: isolated* ***TPyP*** *(dark green line),* ***TPyP*** *in cis-****1***$^{2+}$***•TPyP*** *(blue line) and trans-****1***$^{2+}$***•TPyP*** *(red line).*

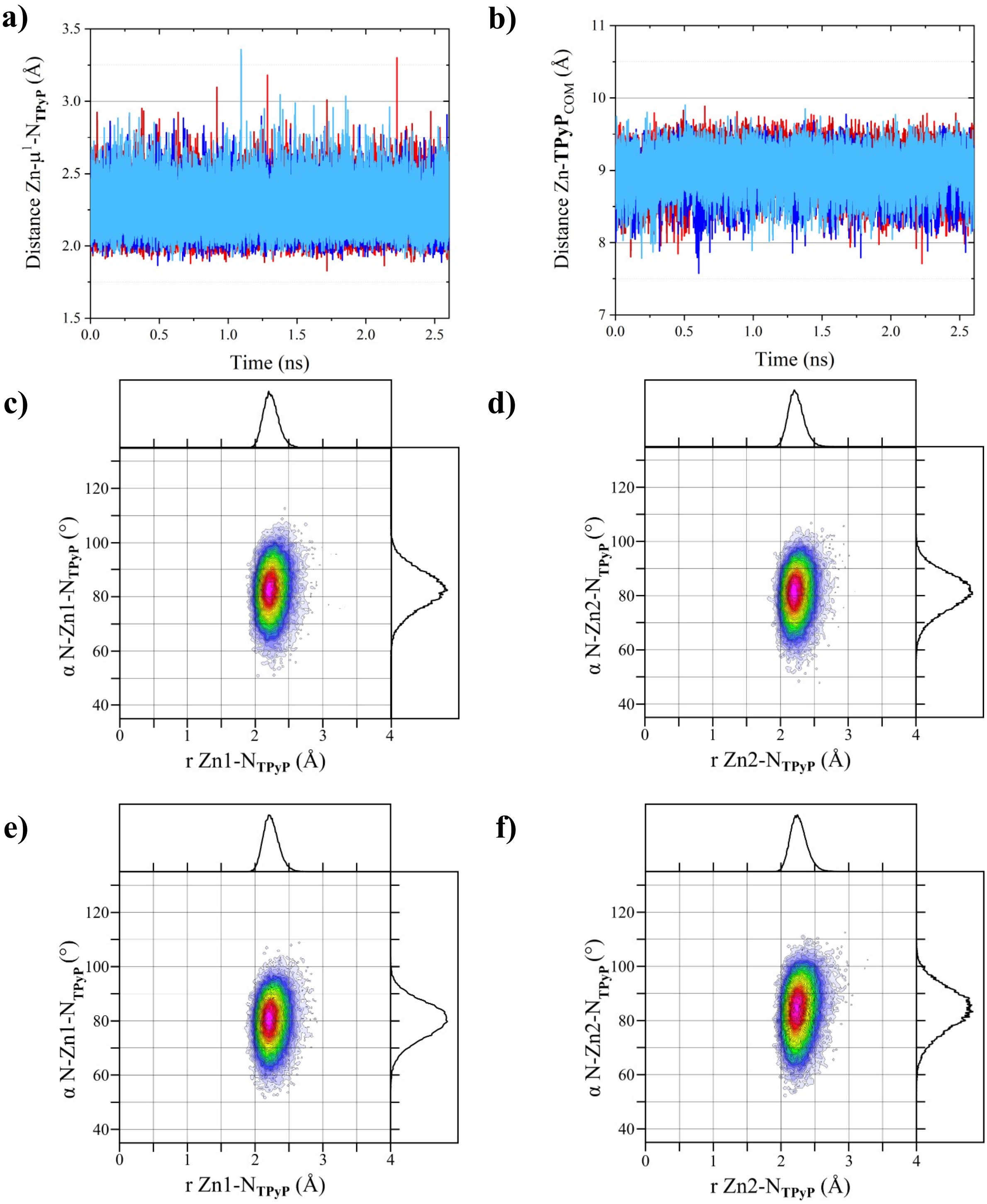

*Fig. S49: a) Distance vs. time between: Zn1 atom of ZnTPP and N atom of Acr for cis-**1**$^{2+}$•**TPyP** (blue line), Zn2 atom of ZnTPP and N atom of Acr for cis-**1**$^{2+}$•**TPyP** (light blue line), Zn1 atom of ZnTPP and N atom of Acr for trans-**1**$^{2+}$•**TPyP** (red line), Zn2 atom of ZnTPP and N atom of Acr for trans-**1**$^{2+}$•**TPyP** (light red line). b) Distance vs. time between: Zn1 atom of ZnTPP and centre of mass (COM) of Acr for cis-**1**$^{2+}$•**TPyP** (blue line), Zn2 atom of ZnTPP and COM of Acr for cis-**1**$^{2+}$•**TPyP** (light blue line), Zn1 atom of ZnTPP and COM of Acr for trans-**1**$^{2+}$•**TPyP** (red line), Zn2 atom of ZnTPP and COM of Acr for trans-**1**$^{2+}$•**TPyP** (light red line). Combined distribution function (CDF) between radial distributions function of Zn1 or Zn2–N$_{TPyP}$ distances and angles distributions function of N-Zn1 or Zn2-N$_{TPyP}$ angles calculated from the MD simulation for: c) and d) trans-**1**$^{2+}$•**TPyP**; e) and f) cis-**1**$^{2+}$•**TPyP**. The two Zn atoms of the ZnTPP moieties have been considered as independent centers.*

Spectroelectrochemical measurements

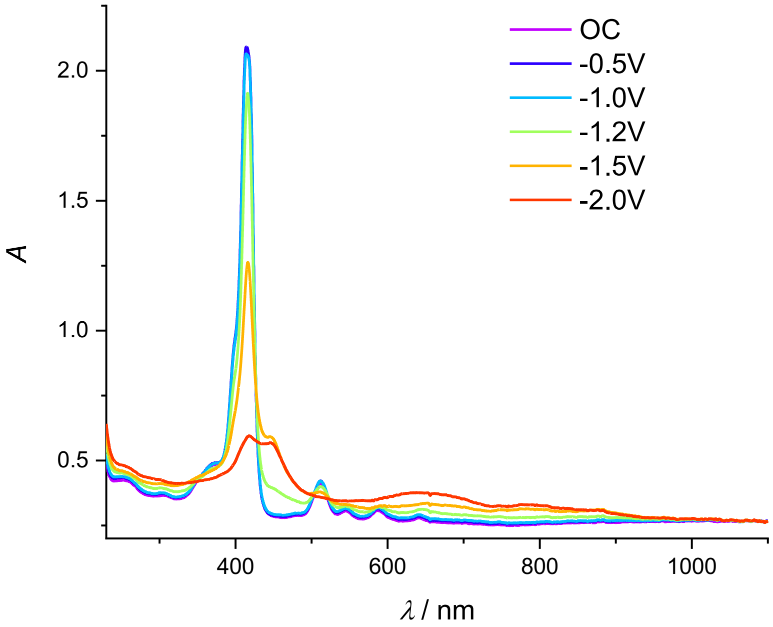

Fig. S50: Spectral variations of a solution 7 × 10^{-5} M of ***TPyP*** *in CH_2Cl_2 upon application of different potentials (OC: open circuit). Working electrode: platinum gauze; reference electrode: Ag/$AgNO_3$ 0.01M in CH_3CN; counter electrode: Pt wire.*

Additional photophysical data for complex 1^{2+}·TPyP in CH_2Cl_2

As previously observed,[6] in the TA spectrum of **1**$^{2+}$ (Fig. S52) the features of model **ZnTPP** (Fig. S51) are nearly absent, except for the characteristic band of the singlet at 1270 nm here detectable,[30] which decays in about 0.6 ps. On the other hand, the formation of the charge separated species **Acr$^•$-ZnP$^{•+}$** is also clearly observable**.** The typical features of **Acr$^•$** are found at 480 and 520 nm,[6,31] whereas the fingerprints of **ZnP$^{•+}$** are detected between 600 and 700 nm[6,31c,31d,32] and around 900 nm.[32] These bands form in 0.6 ps and decay in ca. 3 ps, as already discussed (Fig. S52b).[6] This very short lifetime in CH_2Cl_2 is compatible with the lack of an emission signal for **1**$^{2+}$ in the NIR region.

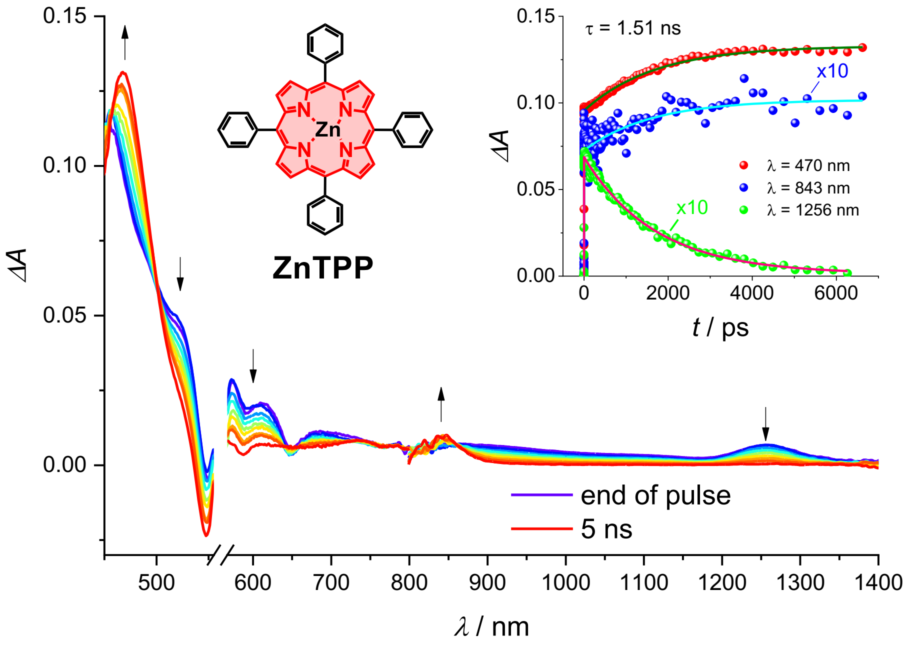

*Fig. S51: Transient absorption (TA) spectra of **ZnTPP** model in CH_2Cl_2. Inset: TA decays at selected wavelengths with the relative fittings (lines). λ_{exc} = 565 nm, E = 8 μJ/pulse.*

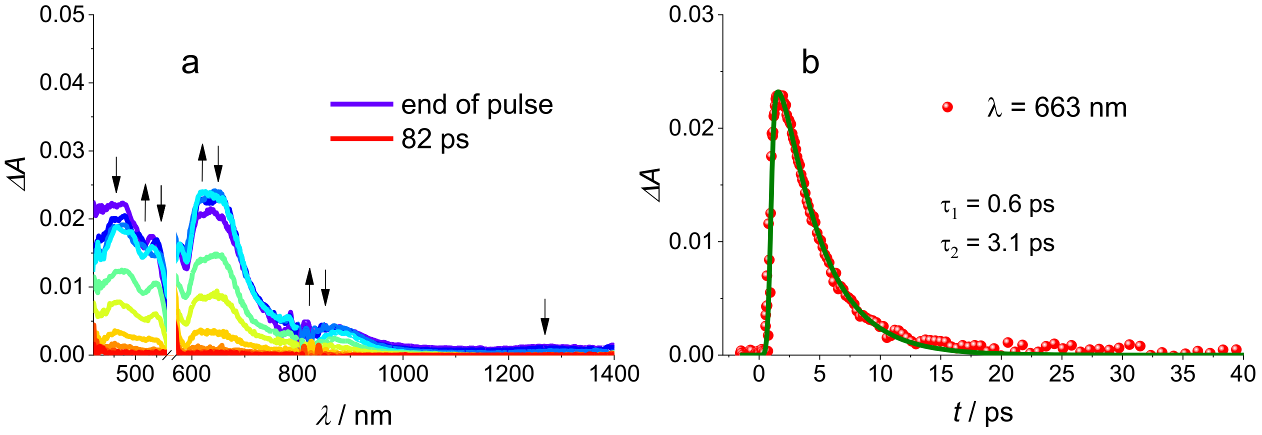

*Fig. S52: a) TA spectra of **1**$^{2+}$ in CH_2Cl_2 (λ_{exc} = 565 nm, E = 8 μJ/pulse). b) TA kinetics at selected wavelengths with the relative fittings (lines).*

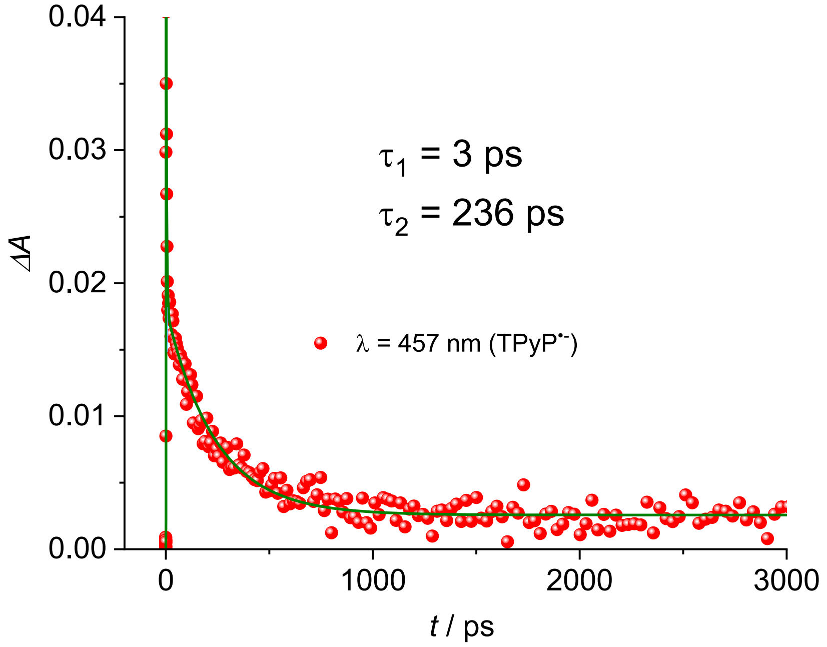

*Fig. S53: TA decay of **1^2+^•TPyP** at 457 nm in CH_2Cl_2 with the relative fitting (line). λ_{exc} = 565 nm (prevalent on ZnP of **1**), E = 8 μJ/pulse. On this time-scale the rise of 0.6 ps is not detectable.*

The transient absorption spectrum of **TPyP** in CH_2Cl_2 is reported in Fig. S54a. Several bands, consistent with positive absorption overlapped with ground state bleaching and stimulated emission, are found all across the visible till the NIR spectral region (450-1150 nm), decaying with a lifetime of over 7 ns (Fig. S54b). This signal can be ascribed to the singlet state, which is found to live 7.6 ns by emission measurements (Table S1).

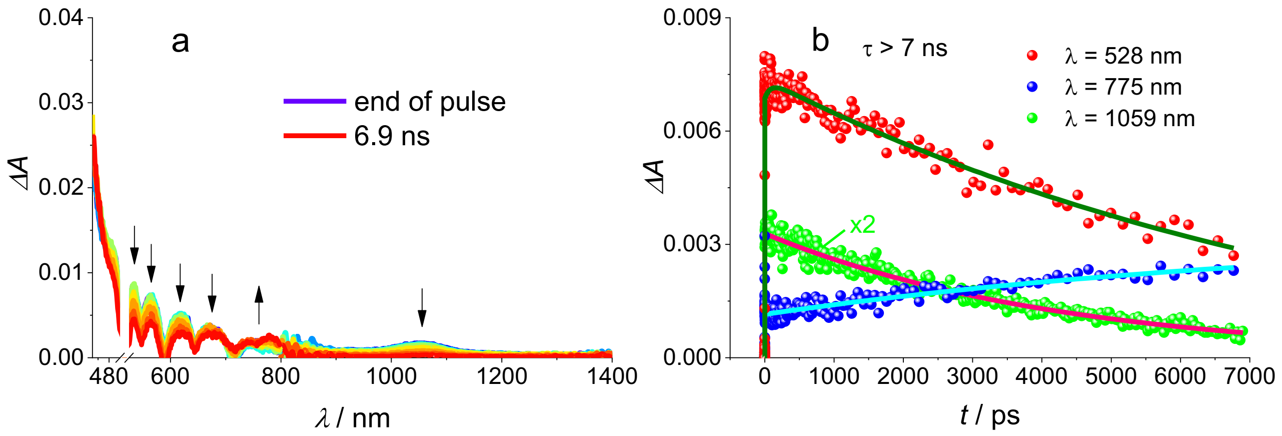

*Fig. S54: a) TA spectra of **TPyP** in CH_2Cl_2 (λ_{exc} = 510 nm, E = 2.8 μJ /pulse). b) TA kinetics at selected wavelengths with the relative fittings (lines).*

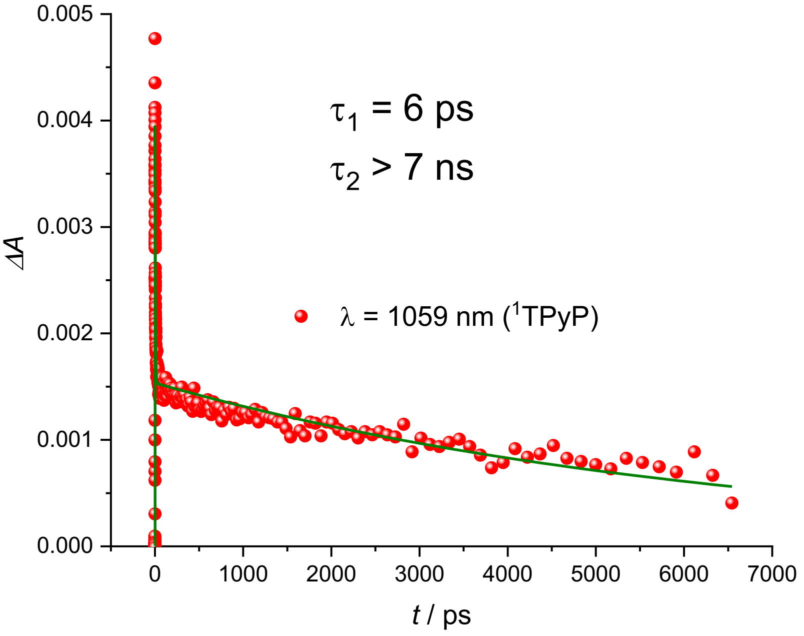

Fig. S55: TA decay of ***1^{2+}·TPyP*** *at 1059 nm in CH_2Cl_2 with the relative fitting (line). λ_{exc} = 510 nm (prevalent on* ***TPyP****), E = 8 μJ/pulse.*

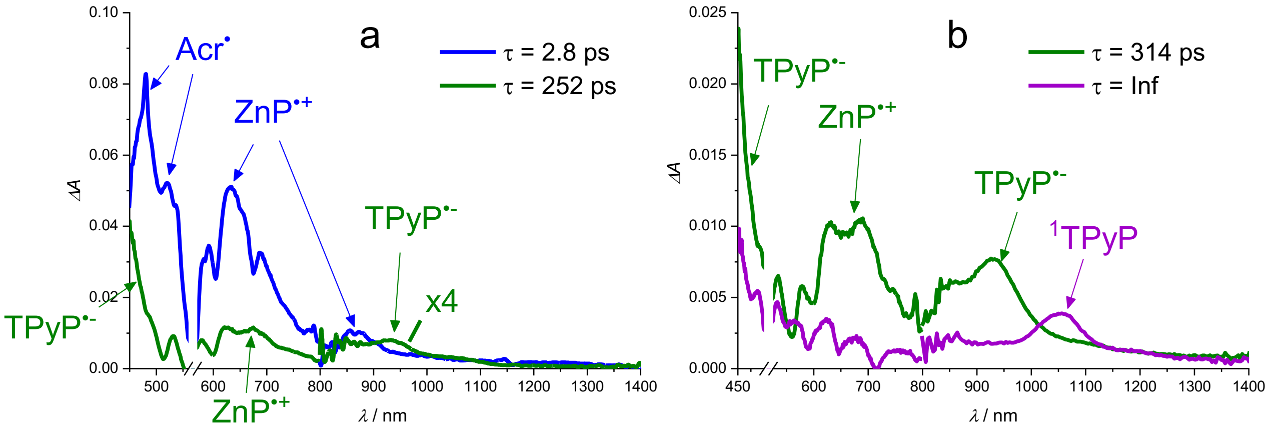

Fig. S56: Spectral distributions of the pre-exponential coefficients and the corresponding lifetimes from global fit analysis of the transient absorption maps of complex ***1^{2+}·TPyP*** *in CH_2Cl_2 upon excitation at a) 565 nm and b) 510 nm.*

Additional photophysical data for complex 1^{2+}·TPyP in toluene

As already reported[6] and in analogy with what observed in CH_2Cl_2, the main bands of $\mathbf{1}^{2+}$ in toluene (Fig. S58) are the singlet band at 1290 nm (that can be observed in the model **ZnTPP**, Fig. S57) and the bands assigned to **Acr**$^{\bullet}$ and **ZnP**$^{\bullet+}$ that form in 6 ps and decay in 200 ps.

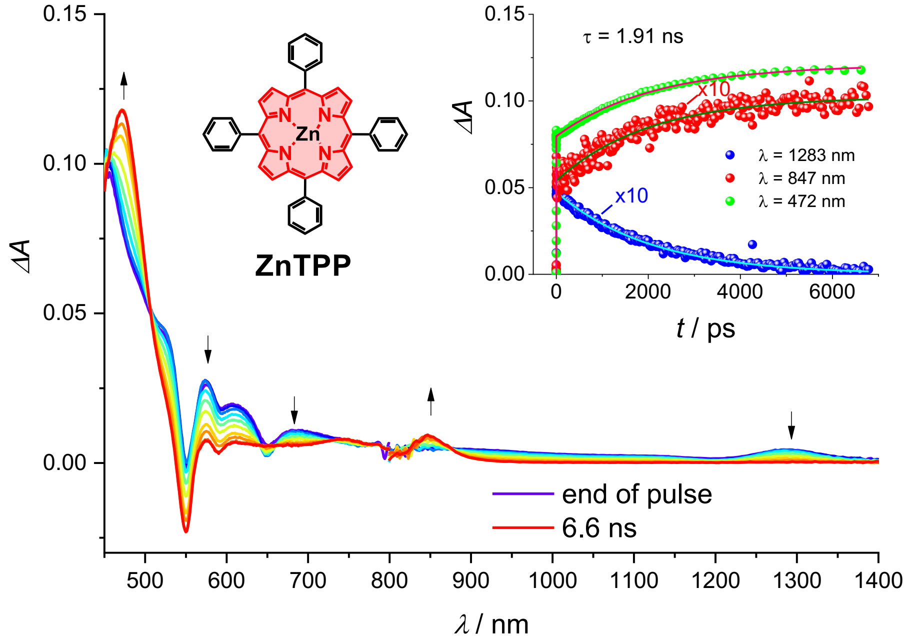

*Fig. S57: TA spectra of **ZnTPP** model in toluene. Inset: TA decays at selected wavelengths with the relative fittings (lines).* λ_{exc} *= 565 nm, E = 8 μJ/pulse.*

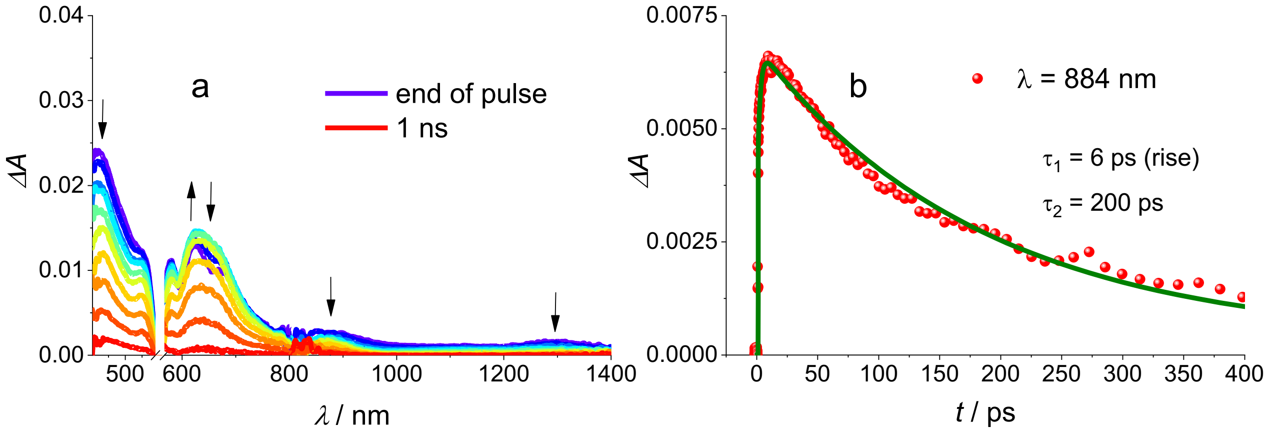

Fig. S58: a) TA spectra of $\mathbf{1}^{2+}$ *in in toluene (*λ_{exc} *= 565 nm, E = 8 μJ/pulse). b) TA kinetics at selected wavelengths with the relative fittings (lines).*

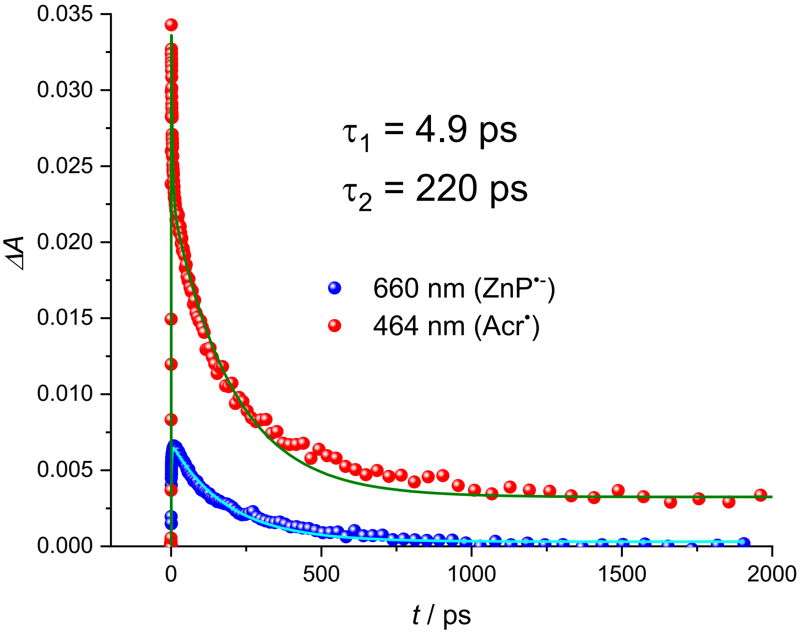

Fig. S59: TA decays of ***1^{2+}•TPyP*** *at 464 and 660 nm in toluene with the relative fittings (lines). λ_{exc} = 565 nm (prevalent on ZnP of* ***1^{2+}****). E = 8 μJ/pulse.*

Fig. S60 shows the transient absorption features of **TPyP** in toluene are. The TA spectrum reveals the same ^{1}**TPyP** bands recorded in CH_2Cl_2, which decay with very long kinetics (longer than the maximum instrumentation window), in line with the 10.6 ns fluorescence lifetime (Table S2). In this case a first fast process (ca. 20 ps) of formation of the signal is observed (Fig. S61) and can be ascribed to vibrational cooling or solvent-induced vibrational energy redistribution.[33]

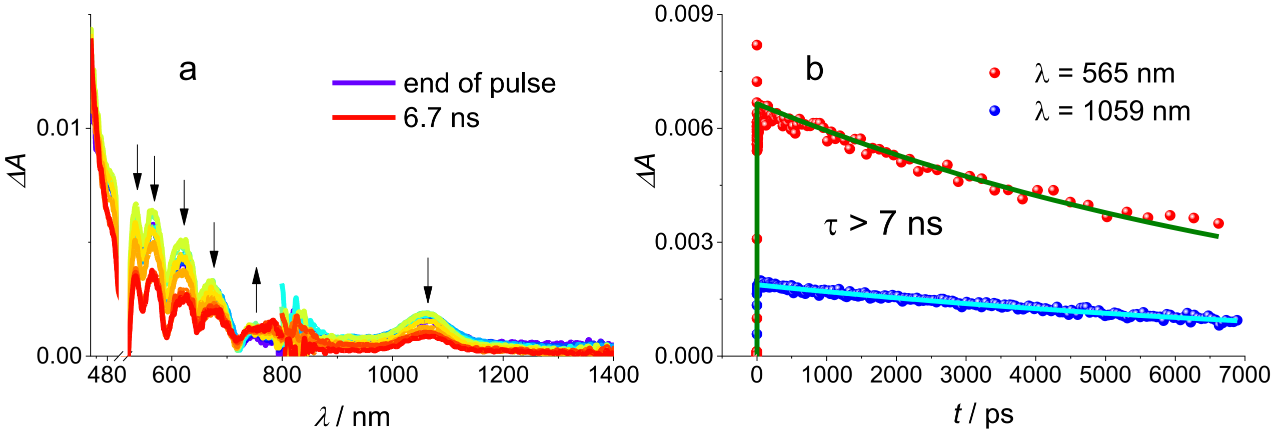

Fig. S60 a) TA spectra of ***TPyP*** *in toluene (λ_{exc} = 510 nm, E = 2.8 μJ/pulse). b) TA kinetics at selected wavelengths with the relative fittings (lines).*

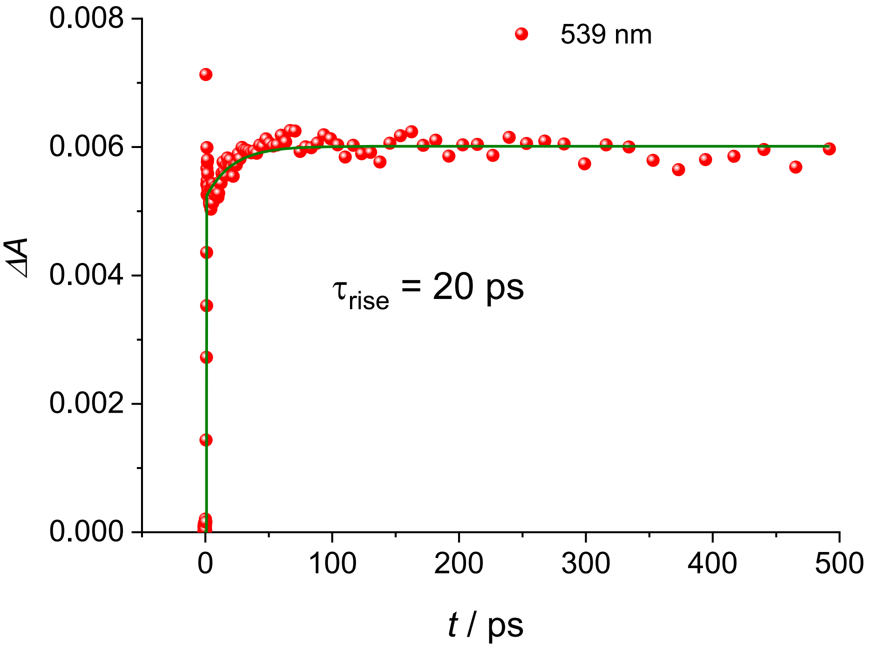

*Fig. S61: TA kinetics of **TPyP** at 539 nm in toluene with the relative fitting (line). λ_{exc} = 510 nm, E = 8 μJ/pulse.*

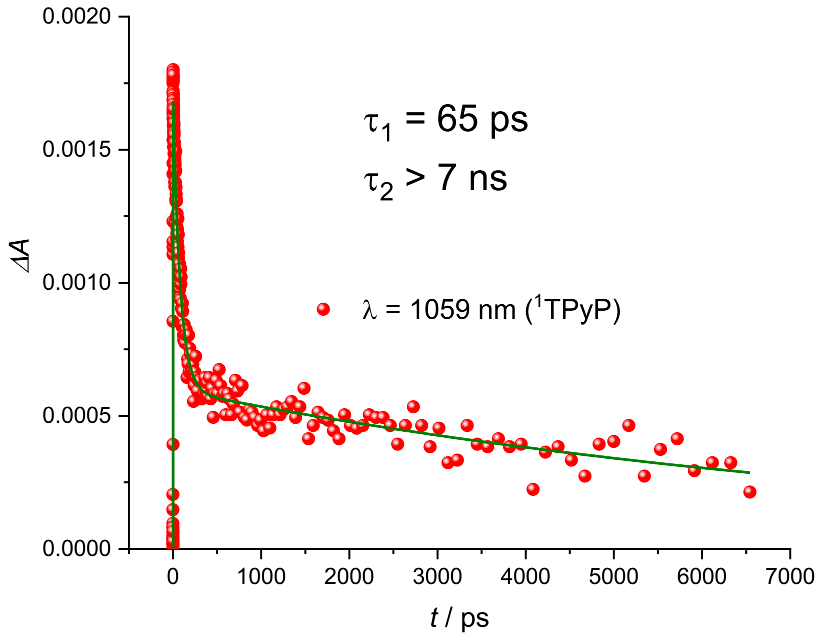

*Fig. S62: TA decay of **1^{2+}•TPyP** at 1059 nm in toluene with the relative fitting (line). λ_{exc} = 510 nm with (prevalent on **TPyP**), E = 8 μJ/pulse.*

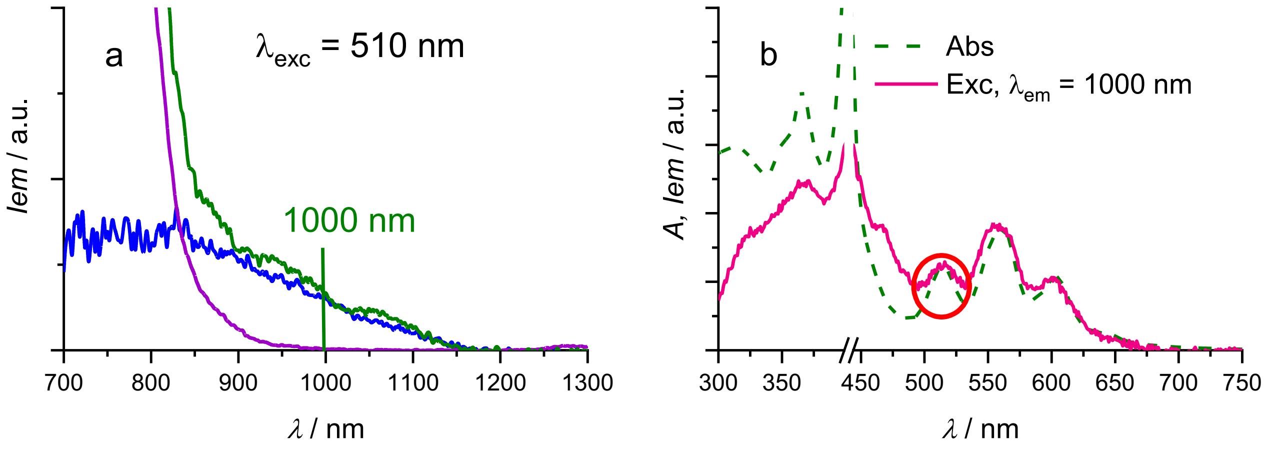

Fig. S63: a) Comparison of the NIR corrected emission spectra of complex ***1^{2+}·TPyP*** *(green) in toluene with the spectra of solutions of model* ***TPyP*** *(violet) at the same concentration as in the mixtures. λ_{exc} = 510 nm. b) Comparison between the excitation spectrum of the complex* ***1^{2+}·TPyP*** *in toluene (λ_{em} =1000 nm) and its arbitrarily scaled absorption features. The circle highlights the Q-band typical only of* ***TPyP****.*

References

[1] J. N. Demas, G. A. Crosby, *J. Phys. Chem.* **1971**, *75*, 991-1242.
[2] P. G. Seybold, M. Gouterman, *J. Mol. Spectrosc.* **1969**, *31*, 1-13.
[3] K. Rurack, M. Spieles, *Anal. Chem.* **2011**, *83*, 1232-1242.
[4] L. Flamigni, A. M. Talarico, B. Ventura, *J. Porphyr. Phthalocyanines* **2003**, *7*, 318-327.
[5] R. M. Williams, M. Koeberg, J. M. Lawson, Y. Z. An, Y. Rubin, M. N. PaddonRow, J. W. Verhoeven, *J. Org. Chem.* **1996**, *61*, 5055-5062.
[6] A. Edo-Osagie, D. Serillon, F. Ruani, X. Barril, C. Gourlaouen, N. Armaroli, B. Ventura, H.-P. Jacquot de Rouville, V. Heitz, *J. Am. Chem. Soc.* **2023**, *145*, 10691-10699.
[7] M. Beyler, L. Flamigni, V. Heitz, J.-P. Sauvage, B. Ventura, *Photochem. Photobiol.* **2014**, *90*, 275-286.
[8] M. J. Frisch, G. W. Trucks, H. B. Schlegel, G. E. Scuseria, M. A. Robb, J. R. Cheeseman, G. Scalmani, V. Barone, G. A. Petersson, H. Nakatsuji, X. Li, M. Caricato, A. V. Marenich, J. Bloino, B. G. Janesko, R. Gomperts, B. Mennucci, H. P. Hratchian, J. V. Ortiz, A. F. Izmaylov, J. L. Sonnenberg, Williams, F. Ding, F. Lipparini, F. Egidi, J. Goings, B. Peng, A. Petrone, T. Henderson, D. Ranasinghe, V. G. Zakrzewski, J. Gao, N. Rega, G. Zheng, W. Liang, M. Hada, M. Ehara, K. Toyota, R. Fukuda, J. Hasegawa, M. Ishida, T. Nakajima, Y. Honda, O. Kitao, H. Nakai, T. Vreven, K. Throssell, J. A. Montgomery Jr., J. E. Peralta, F. Ogliaro, M. J. Bearpark, J. J. Heyd, E. N. Brothers, K. N. Kudin, V. N. Staroverov, T. A. Keith, R. Kobayashi, J. Normand, K. Raghavachari, A. P. Rendell, J. C. Burant, S. S. Iyengar, J. Tomasi, M. Cossi, J. M. Millam, M. Klene, C. Adamo, R. Cammi, J. W. Ochterski, R. L. Martin, K. Morokuma, O. Farkas, J. B. Foresman, D. J. Fox, Wallingford, CT, **2016**.
[9] a) J.-D. Chai, M. Head-Gordon, *Phys. Chem. Chem. Phys.* **2008**, *10*, 6615-6620; b) J. D. Chai, M. Head-Gordon, *J. Chem. Phys.* **2008**, *128*, 084106.
[10] a) A. Schäfer, H. Horn, R. Ahlrichs, *J. Chem. Phys.* **1992**, *97*, 2571-2577; b) A. Schäfer, C. Huber, R. Ahlrichs, *J. Chem. Phys.* **1994**, *100*, 5829-5835.
[11] a) A. Duque-Prata, T. B. Pinto, C. Serpa, P. J. S. B. Caridade, *ChemistrySelect* **2023**, *8*, e202300205; b) P. Jayachandran, A. Angamuthu, P. Gopalan, *J. Chem. Sci.* **2022**, *134*, 29.
[12] M. Cossi, V. Barone, R. Cammi, J. Tomasi, *Chem. Phys. Lett.* **1996**, *255*, 327-335.
[13] E. Runge, E. K. U. Gross, *Phys. Rev. Lett.* **1984**, *52*, 997-1000.
[14] R. L. Martin, *J. Chem. Phys.* **2003**, *118*, 4775-4777.
[15] C. Bannwarth, S. Ehlert, S. Grimme, *J. Chem. Theory Comput.* **2019**, *15*, 1652-1671.
[16] C. Bannwarth, E. Caldeweyher, S. Ehlert, A. Hansen, P. Pracht, J. Seibert, S. Spicher, S. Grimme, *Wiley Interdiscip. Rev. Comput. Mol. Sci.* **2021**, *11*, e1493.
[17] a) M. Elstner, *Theor. Chem. Acc.* **2006**, *116*, 316-325; b) D. Veclani, A. Melchior, A. Llobet, N. Armaroli, A. Venturini, *Comput. Mater. Sci.* **2023**, *219*, 111997.
[18] a) R. Ferrero, S. Pantaleone, M. Delle Piane, F. Caldera, M. Corno, F. Trotta, V. Brunella, **2021**, *26*, 5881; b) P. Wróbel, P. Kubisiak, A. Eilmes, *J. Phys. Chem. B* **2022**, *126*, 10922-10932; c) P. Wróbel, A. Eilmes, **2023**, *28*, 6736; d) J. A. Platts, **2020**, *7*, 191562; e) R. Schnegotzki, J. Koopman, S. Grimme, R. D. Süssmuth, *Chem. Eur.* **2022**, *28*, e202200318.
[19] S. Ehlert, M. Stahn, S. Spicher, S. Grimme, *J. Chem. Theory Comput.* **2021**, *17*, 4250-4261.
[20] A. S. Lemak, N. K. Balabaev, *Mol. Simul.* **1994**, *13*, 177-187.
[21] K. D. Hammonds, J.-P. Ryckaert, *Comput. Phys. Commun.* **1991**, *62*, 336-351.
[22] W. Humphrey, A. Dalke, K. Schulten, *J. Mol. Graph.* **1996**, *14*, 33-38.
[23] M. Brehm, M. Thomas, S. Gehrke, B. Kirchner, *J. Chem. Phys.* **2020**, *152*.
[24] C. Lefebvre, J. Klein, H. Khartabil, J.-C. Boisson, E. Hénon, *J. Comput. Chem.* **2023**, *44*, 1750-1766.
[25] B. Ventura, L. Flamigni, G. Marconi, F. Lodato, D. L. Officer, *New. J. Chem.* **2008**, *32*, 166-178.
[26] a) M. N. Manaia, A.-M. Chiorcea-Paquim, *J. Electroanal. Chem.* **2023**, *929*, 117123; b) T. A. Evans, G. S. Srivatsa, D. T. Sawyer, T. G. Traylor, *Inorg. Chem.* **1985**, *24*, 4733-4735.
[27] A. Banyasz, L. Martinez-Fernandez, T.-M. Ketola, A. Muñoz-Losa, L. Esposito, D. Markovitsi, R. Improta, *J. Phys. Chem. Lett.* **2016**, *7*, 2020-2023.
[28] C. C. Wamser, A. Ghosh, *J. Am. Chem. Soc.* **2022**, *2*, 1543-1560.
[29] M. Busato, G. Mannucci, L. A. Rocchi, M. E. Di Pietro, A. Capocefalo, E. Zorzi, P. Casu, D. Veclani, F. Castiglione, A. Mele, A. Martinelli, P. Postorino, P. D'Angelo, *ACS Sustain. Chem. Eng.* **2023**, *11*, 8988-8999.
[30] D. B. Moravec, B. M. Lovaasen, M. D. Hopkins, *J. Photoch. Photobio. A* **2013**, *254*, 20-24.

[31] a) S. Fukuzumi, K. Ohkubo, T. Suenobu, K. Kato, M. Fujitsuka, O. Ito, *J. Am. Chem. Soc.* **2001**, *123*, 8459-8467; b) M. Tanaka, K. Ohkubo, C. P. Gros, R. Guilard, S. Fukuzumi, *J. Am. Chem. Soc.* **2006**, *128*, 14625-14633; c) A. Edo-Osagie, D. Sanchez-Resa, D. Serillon, E. Bandini, C. Gourlaouen, H.-P. Jacquot de Rouville, B. Ventura, V. Heitz, *C. R. Chim.* **2021**, *24*, 47-55; d) F. Ruani, A. Edo-Osagie, H.-P. Jacquot de Rouville, V. Heitz, B. Ventura, N. Armaroli, *J. Porphyr. Phthalocyanines* **2023**, *27*, 569-575; e) A. C. Benniston, A. Harriman, P. Y. Li, J. P. Rostron, H. J. van Ramesdonk, M. M. Groeneveld, H. Zhang, J. W. Verhoeven, *J. Am. Chem. Soc.* **2005**, *127*, 16054-16064; f) K. Ohkubo, K. Suga, K. Morikawa, S. Fukuzumi, *J. Am. Chem. Soc.* **2003**, *125*, 12850-12859.

[32] E. A. Aleman, J. Manriquez Rocha, W. Wongwitwichote, L. A. Godinez Mora-Tovar, D. A. Modarelli, *J. Phys. Chem. A* **2011**, *115*, 6456-6471.

[33] a) Y. Venkatesh, M. Venkatesan, B. Ramakrishna, P. R. Bangal, *J. Phys. Chem. B.* **2016**, *120*, 9410-9421; b) J. S. Baskin, H.-Z. Yu, A. H. Zewail, *J. Phys. Chem. A* **2002**, *106*, 9837-9844.
